# Psychosocial needs among older perinatally infected adolescents living with HIV and transitioning to adult care in Kenya

**DOI:** 10.1371/journal.pone.0233451

**Published:** 2020-07-29

**Authors:** Nyawira Gitahi, Carol Camlin, Veronica Mwania, Kenneth Ngure, Colette Auerswald, Elizabeth Bukusi

**Affiliations:** 1 Institute of Tropical Medicine and Infectious Disease, Kenya Medical Research Institute, Nairobi, Kenya; 2 Department of Environmental Health and Disease Control, Jomo Kenyatta University of Agriculture and Technology, Nairobi, Kenya; 3 Department of Obstetrics, Gynaecology & Reproductive Sciences, University of California, San Francisco, California, United States of America; 4 Department of Community Health, Jomo Kenyatta University of Agriculture and Technology, Nairobi, Kenya; 5 Innovations for Youth (i4Y) and Community Health Sciences, University of California, Berkeley School of Public Health, Berkeley, California, United States of America; 6 Centre for Microbiology Research, Kenya Medical Research Institute, Nairobi, Kenya; University of South Florida, UNITED STATES

## Abstract

**Background:**

Little data is available on the long-term psychosocial effects of disclosure of HIV status that may occur in late adolescence, even when disclosure is timely. Moreover, few studies have described the post-disclosure psychosocial needs of older adolescents who experience delayed disclosure. This study sought to address existing knowledge gaps in the post-disclosure experiences and psychosocial needs of older adolescents living with HIV (ALWHIV).

**Methods:**

We conducted focus group discussions (FGDs) and in-depth interviews (IDIs) among older perinatally infected adolescents aged 16–19 years We collected socio-demographic data and baseline viral load (copies/ml) results for the preceding six months using interviewer-administered questionnaires and clinical notes abstraction. We analysed data inductively and deductively to identify themes related to the experiences and expectations of adolescents with the disclosure and post-disclosure period.

**Results:**

Adolescents who reported having received timely disclosure expressed that as they grew older, they began to comprehend the lifelong repercussions of an HIV diagnosis and experienced a re-emergence of the negative feelings similar to those experienced during the post-disclosure period. Those who received the knowledge of their HIV status during late adolescence experienced prolonged periods of negative self-perception and anger at not receiving their HIV status earlier. They also expressed a need for more information during the disclosure process on the prevention of onward transmission of the virus, safe conception practices resulting in HIV negative children, and information on how to disclose their HIV status to sexual partners or peers. Anticipated stigma was experienced universally by these older adolescents and was a major barrier towards adherence and coping with an HIV status. Caregivers or siblings with a similar HIV status were a source of social support. Adolescents felt that the support of peers (ALWHIV) helped them to accept their HIV status and to learn how to develop a positive outlook on life.

**Conclusion:**

Provision of psychosocial care in late adolescence during the transition to adult care is critical in ensuring the resolution of re-emergent negative emotions. Comprehensive information on HIV prevention and sexual reproductive health should be a crucial component of post-disclosure care for older adolescents. HIV Disclosure and adolescent transition guidelines should include these components to optimize psychosocial care for older adolescents.

## Introduction

There are 1.3 million adolescents aged 10 to 19 living with perinatally acquired HIV infection in the sub-Saharan African region [1–4]. They often have to balance coping with psychosocial challenges, maintaining adherence to ART and learning to negotiate sexual relationships [5–7]. Studies report that the highest morbidity and mortality rates among PLWHIV are among older adolescents (15–24 years old) and young adults of the age to be transitioning to adult care (i.e., care for individuals >25 years old) [8].

The transition of adolescents has been described as a purposeful, planned process that addresses the medical, psychosocial, educational, and vocational needs of adolescents and young adults with chronic medical conditions. to advance them from a pediatric and family-centred to an adult, individual-focused health care provider [9]. This process is often hindered by the sub-optimal psychological preparation of the adolescents, which begins with the disclosure of their HIV status [10].

The WHO guidelines on disclosure and post-disclosure psychosocial support for HIV infected children recommend timely incremental disclosure beginning at age seven, a process which should be completed by age 12 [11]. This guidance has not incorporated recommendations on post-disclosure support, where disclosure is delayed or on the content that should be addressed during the period of transition. A review of data from eleven countries, primarily from sub-Saharan Africa reported rates of disclosure by the age of 12 that ranged from 0.1% to 50% [12]. A study from Kenya reported that 36% of adolescents knew about their HIV status by the age of 12 [13], which indicates that the disclosure of HIV status for the majority of adolescents and young adults currently in care occurred during mid to late adolescence (14–19)years of age [12,13]. This is despite the existing evidence that timely disclosure improves adherence to ART [7,14,15] and facilitates the development of coping skills among adolescents living with HIV [16–19]. With the current data suggesting that disclosure timing is often delayed (after the age of twelve), post-disclosure experiences and psychosocial needs of older adolescents are largely undescribed. This knowledge could guide recommendations on how to strengthen current guidelines on disclosure to suit the needs of older adolescents and young adults transitioning to adult HIV care. The objective of this study was to explore the circumstances surrounding the phenomena disclosure process, post-disclosure emotional experiences, expectations and informational needs of older ALWHIV during the transition to adult care.

## Methodology

### Study design

We carried out a cross-sectional qualitative study. The findings in this paper are part of a larger mixed-methods study that explored the psychosocial needs of adolescents during the transition to adult care. In this paper, we present the results of our analysis of data from focus groups (FGDs) and individual semi-structured interviews (IDIs) and describe the disclosure process, the post-disclosure feelings and unvoiced or voiced expectations of our adolescent participants.

### Study setting

The study was conducted in two high-volume urban HIV clinics that were purposively selected: Mbagathi Hospital and Kenyatta National Hospital in Nairobi, the capital city of Kenya. We use the following selection criteria: 1) clinics that were well-established in paediatric HIV care over the previous 15 years, and 2) clinics that were currently providing HIV treatment to large cohorts of perinatally infected ALWHIV.

### Sampling and recruitment

During routine clinic visits, we screened ALWHIV for study eligibility. The study criteria included being 16 to 19 years of age, documented perinatal infection, and enrolment into HIV care and ART for at least three years. We explained the objectives of the study during the informed consent process.

As part of the larger mixed-methods study, during study enrolment we collected: 1) socio-demographic data, including the HIV status of the caregiver (primary caregiver indicated in the inpatient medical file) and of siblings; 2) medical history, time of disclosure (timely disclosure was defined as having occurred before the age of 12), duration of ART and viral load; and 3) psychosocial measures, such as the experience of stigma, ART self-efficacy, and self-esteem.

We collected these data with a combination of interviewer-administered questionnaires and chart abstraction. We confirmed perinatal infection through review of clinic records for a record of HIV DNA or antibody tests within the postnatal and infancy period or by using a composite proxy, i.e., clinic enrolment during early childhood and or duration of more than three years on ART and documented positive HIV status of the mother where available. These proxies were used to determine perinatal infection where there was no documentation of an infant PCR or HIV antibody testing in the clinical notes. Often in poor resource settings, paper-based medical records documenting an initial HIV diagnosis that occurred over a decade before may no longer be available, hence the need to include proxies.

We enrolled youth into FGDs based on sex and viral load. The rationale for stratification by sex was to decrease the inhibition of participating adolescents who often exhibit high self-awareness and self-consciousness, particularly in the presence of the opposite sex. We separated groups by viral load to provide a safe environment for adolescents to speak about any psychosocial issues or other factors related to poor adherence without fear of judgement from peers who may not have experienced these challenges.

We conducted FGDs in these four sub-categories. After a preliminary analysis of the data from the FGDs, we held IDI’s with participants who were enrolled by did not take part in the FGDs

### Data collection

We collected data in December 2017 and December 2018 during the long school holidays as the majority of the adolescents were in boarding schools. Using pre-tested structured topic guides the FGDs provided a forum for participants to share, compare, and contrast their experiences regarding their experiences with disclosure with their peers. In contrast, IDIs provided individual adolescents with an opportunity to reflect and provide rich, detailed descriptions of their disclosure experiences. We conducted 8 FGDs (n = 48) and after a preliminary analysis to determine saturation, subsequently conducted 10 IDIs with ten additional participants.

Four social science graduate research assistants (three females and one male) trained in qualitative research and experienced in conducting interviews with adolescents qualitative data collection), train. We employed non-clinically-trained research assistants who had not interacted with the adolescents to reduce the risk of social desirability bias. The study involved a single contact with each study participant, during which the interviewer provided an overview of the study objectives, and obtained informed consent from the participant before proceeding with the FGD or IDI. The study received ethical approval from the Kenyatta National Hospital /University of Nairobi Ethical and Research committee. We obtained written informed consent from all participants and their caregivers. No youth approached for participation refused to do so.

The FGD and IDI guides explored the adolescents’ experiences during the disclosure process, any expectations they had, whether they had any unvoiced concerns or questions, and what emotions they experienced post the disclosure process. Using pre-tested structured topic guides the FGDs provided a forum for participants to share, compare, and contrast their experiences regarding their experiences with disclosure with their peers. In contrast, IDIs provided individual adolescents with an opportunity to reflect and provide rich, detailed descriptions of their disclosure experiences. Interviews and focus groups were audio-recorded. Focus groups lasted 60 to 90 minutes, while interviews lasted 30 to 45 minutes. Participants were reimbursed for their transport to the clinic and provided with snacks.

### Data analysis

We conducted a thematic analysis of our data. Using a deductive and inductive approach, the process of transcription and reading of the transcripts provided an insight into emerging themes and was an essential part of preliminary data analysis. During the data collection period, the research team held biweekly meetings during which researchers compared notes and shared their understanding of the emerging themes. We used a codebook to define a priori codes generated from the FGD and IDI guides, which were later supplemented by codes emerging from the transcripts themselves. Once the codebook was completed, coding was carried out independently by two individuals with discussions held with the research team regarding any differences that emerged.

To complement the coding process, we reviewed socio-demographic profiles of the participants from the quantitative data to elicit the context of the participant's views. Transcripts and corresponding quantitative data were uploaded into Dedoose software version 8.2.27, to enable more systematic management, coding and retrieval of data. We considered questions of reflexivity throughout the study period, identifying and reflecting on our assumptions and preconceptions regarding the anticipated post-disclosure emotions. During the analysis, we explored comparisons by viral load, gender and time of disclosure. These comparisons did not come out clearly except for the time of disclosure, and therefore, we have not presented the findings according to these stratifications. This study used the Consolidated criteria for reporting qualitative research (COREQ) [20].

## Results

### Baseline data

Fifty-eight adolescents participated in either the FGDs (19 females and 29 males) or IDIs (6 females and 4 males), of whom 27 were virally suppressed, and 31 unsuppressed). Their median age at the time of disclosure was 13 years (IQR (13–16)). Only 9 participants reported having two living parents.

### Qualitative findings

We identified five major themes: the adolescents’ perception of the disclosure process, Antiretroviral adherence and disclosure, post-disclosure feelings, post-disclosure support needs for older adolescents and post-disclosure coping differences. Under the theme post-disclosure feelings, we identified sub-themes; the role of peer and family support, post-disclosure perceptions among adolescents who experienced delayed disclosure versus those with timely disclosure and barriers and facilitators of coping after disclosure of HIV status.

#### The disclosure process

A majority of the adolescents reported that their status had been disclosed to them between the ages of 13 and 16. They shared that disclosure was generally unplanned and often triggered by an event such as the occurrence of an opportunistic infection due to poor adherence, or because caregivers suspected that the adolescent was engaging in risky behaviour.

“I got sick and was taken to the hospital. I was almost 14 yrs. I don’t know what my mum was told, but I know we had to go to that hospital regularly. I was started on drugs. I didn’t take them. I think that’s when they knew they had to tell me” (male, 17 yrs.).

‘I knew about my status in form one (15 years). I fell ill and had sores on my back and all over my body… I turned out HIV positive while my sister was negative… I remember asking her what that meant, and she told me to ask the doctor’ (female, 17 yrs.).

“I was 15 years. How I found out is that one day I went out and came home late and my mum told me why are you disturbing me and yet you are HIV positive? Now at the time, I didn’t take it seriously…that’s how I got to know” (male, 16 yrs.).

The adolescents also described self-discovery of their HIV status, usually associated with reading HIV-related posters or medical records during clinic visits. Interestingly, self-disclosure was more common among male participants. They expressed that they did not discuss their HIV status knowledge with their caregivers who continued accompany them to the clinic for routine care.

“I was walking around the hospital, and I would look at the posters…That’s how I knew.

I didn’t tell them, though, that I knew. I was 13 years” (male,16 yrs.).

“When I was 15 years, my dad left me with my medical file to hold, and I snooped and found that I was HIV positive… I confirmed some of the terms on Google and confirmed my status. I never told him I knew my status. Sometimes when he yelled at me, I felt like telling him that I know I am HIV positive… but I keep it to myself because I didn’t want to look like I am snoop, so I kept it to myself” (male, 18yrs*)*.

#### Antiretroviral adherence and disclosure

There was universal agreement among participants that knowledge of one’s HIV status contributed to enhanced adherence to antiretroviral therapy. The adolescents reported that learning about their HIV status empowered them to understand the consequences of not adhering to medication. They also expressed that knowledge that a parent, relative, or peer died of HIV-related complications enhanced their resolve to adhere to medication and remain free of opportunistic infections.

“I prefer knowing my status because once you know your status, you have to work towards making sure you are okay, and you are healthy and so that you can be strong to move on.”

(female 18 yrs.).

“My dad told me ‘there is something I want to tell you, but don’t be so shocked,’ I asked ‘What?’ “The drugs that you take are for HIV.” I said, “Me, I have HIV, and the way I am healthy?” He told me yes. I asked him where it came from. He told me that my mom had it, and you know my mom had died. I started taking drugs, and I have never stopped” (male 19 yrs.).

“I was told that I have to adhere to my medication, and I took it seriously after seeing that guy I was talking about …who didn’t adhere to his medication, and he ended up dying. So the doctor told me that I should adhere to my drugs. I don’t want to end up like Dennis, that really scared me, and I decided to take my medication continually.” (female, 16yrs.)-).

The disclosure process was not accompanied by standardised information on the importance of ART adherence, including among older adolescents. Even among ALWHIV who learned about their HIV status at a young age, adherence was still a challenge. They were only able to fully comprehend the importance of adherence in ensuring good health outcomes as they grew older.

I: Were you told about adherence on the day they disclosed your status to you?

R1: Yes. I remember when I was young, my mum used to remind me constantly to take them on time. Initially, I didn’t understand why, but with time I got used to it.

I: Do you think such information should have been shared to you on the same day you were told about your status?

R1: Not all it depends on the doctors you find on duty.

I: Anyone else?

R2: I was not told the same day; I was told later on.

I: Were you told immediately?

R2: Yes, I was told everything immediately.

(FGD, Females)

#### Post disclosure feelings

*The role of peer and family support*. Where present, family or caregiver support played a significant role in facilitating the transition from first disclosure of HIV status to developing the internal drive to stay healthy by adhering to medication. ALWHIV, who had a supportive adult in their home or school environment was better equipped to cope with their HIV status. Additionally, the presence of a caregiver or sibling with a similar HIV status resulted in increased openness in sharing their challenges.

“Since I have been getting the support, I am okay. I have accepted my status, and it’s okay. My aunty gives me support, and another matron in school also supports me. I love my parents and my dad and my sister, and they also give me support by making me strong” (female, 18yrs.).

“I don’t speak to my brothers about my status, I am close with my mum, and we talk about my status since we are both HIV positive” (male, 17yrs.).

*Barriers and facilitators of coping after disclosure of HIV status*. A significant barrier to coping with the knowledge of one’s HIV status was a lack of candid conversations about the circumstances of the adolescents’ HIV acquisition and that of their parents. Participants who did not have siblings who were living with HIV also struggled with accepting their HIV status.

Participants also shared that they were able to cope thanks to the support and acceptance offered by their friends and other ALWHIV within support groups. Interestingly, participants who practised or believed in spirituality also felt that this had contributed to enabling them to cope with the knowledge of their HIV status. (Table 1).

**Table 1:** Barriers and facilitators of coping after disclosure of HIV status.

BARRIERS/ FACILITATORS TO ACCEPTANCE	PARTICIPANT QUOTES
FACILITATORS:	
Spirituality and religion	“No, I believe that in this world, many things happen and this is what God thought I could handle, and that is my reasoning for this” (female, 17 yrs.).
Perception of being in control of one owns health	“I prefer knowing my status because once you know your status, you have to work towards making sure you are okay, and you are healthy and so that you can be strong to move on” (female, 17 yrs.).
Friends, peer support groups and acceptance by other ALWHIV	“I learnt from the support group that I have been attending, and I listened to the information that was shared during those sessions by youth like me, and I realised that I have nothing to worry about and I can still live a healthy life regardless of my status” (female, 16 yrs.).
BARRIERS:	“I used to get stressed, in the past, but these days I am comfortable with my status. I was stressed just thinking why I was the only child in my family that was infected yet all my siblings are negative” (male, 17 yrs.).
Being the only HIV positive sibling	
Lack of information on how parents were infected and how HIV was transmitted to themselves	“Actually, I have always wanted to ask my dad what happened and how come my mum died as a result of HIV. It makes me sad we have never talked about” (female, 17 yrs.).

*Re-emergence of negative post-disclosure feelings by adolescents who received timely disclosure*. Adolescents who received up-to-date knowledge of their HIV status (between ages 7 and 12) reported having coped well immediately after disclosure. However, they described feelings of anger and hopelessness arising in later years. These adolescents said that they often faced internal struggles with self-stigma and worried about the impact of HIV on their future relationships.

“I didn’t feel bad because I didn’t really understand much… but gradually, after understanding what it meant to have HIV, I felt bad… I didn’t tell anyone my feelings” (male, 18 yrs.).

“I was young; I just listened and took my meds; now I understand this thing. I wonder how I got it—was it my dad or my mum who brought it, and how other people will see, what if other people know that I am HIV positive? What will be their reaction? Other youths or teens who do not know about my HIV status?” (female,17 yrs.).

*Post-disclosure feelings among adolescents who experienced delayed disclosure*. Participants who experienced delayed disclosure (after 12 years), reported immediately feeling anger and disappointment about their status. These strong feelings took more than a year to resolve. Among those who reported delayed disclosure (after age 12), participants agreed that the process of self-acceptance was prolonged and that their ideal time of disclosure would have been when they were younger, as they felt it would have been easier to accept. Participants also expressed that acceptance of their HIV status was a significant barrier to adherence.

“I would want to be told when I was like in class 3 (9 years) because that is when I would have accepted well, then she would have taken me through a process where I would have accepted myself how I am” (female, 17yrs.).

“If they told me early on about my status, it would not have taken me that long to accept my situation. I was told when I was in class 6 (12 years), and I took up to 2 years to accept myself, and I started feeling that my mother didn’t love me because if she did, then she would have told me earlier on” (male, 16 yrs.).

Participants expressed concerns about the future, in particular regarding marriage, onward transmission and acceptance from peers and in their future relationships.

“What if my wife were to find out that I am HIV positive?” (male, 18 yrs.).

“I would like to have been told more about marriage. How you can live with your wife and the children not get infected and how you can get a negative family” (male, 17 yrs.).

Disclosure was often delayed due to anticipated stigma by the caregivers or parents towards the child. The adolescents agreed that this constant fear of stigma further fuelled their feeling of low self-worth and anticipated lack of acceptance by peers (Table 2). Participants eventual acceptance was facilitated by access to knowledge about HIV and its life-long management. Often, respondents chose to keep their feelings and questions to themselves upon discovering their HIV status. One typical unvoiced question was the genesis of their infection. Participants hesitated to ask out of a desire to protect their caregivers emotionally. These questions largely remained unresolved until late adolescence. Participants often also hid their despair even from immediate family or caregivers.

**Table 2:** Post-disclosure negative feelings among adolescents with delayed disclosure (illustrative notes).

**Anticipated stigma**
“After my status was disclosed to me, I wanted to lock myself in the house, I didn’t feel like coming out of the house, and at that time there was a lot of stigma that I didn’t want to hear about and that most people did not have the information. But now I feel better, and that is due to talking to several people over time” (female, 16yrs).
“No, because when you are young, you can talk and disclose your status, so they lied to me that I was taking drugs to cure a cold” (female, 17yrs.).
“I felt that I was in another world, so when I look at my brother, I feel like they are in another world and I am in another world. So there are two worlds” (male,16 yrs.)”
**A prolonged period of a negative perception of self**
“It was not good, and I kept thinking about this life of having to take medicine the rest of my life, but after counselling, I went back home pretending to be happy but deep inside I was not happy. Before I was told I was social, and I had no fear, but after being told my status, I became less social and quieter. It took me like three years to cope and be able to take medicine without feeling anything” (male, 18 yrs.).
**Concerns about the future**
“Before I didn’t think about it. But now, it bothers me. What if my wife-to-be found out that I am HIV positive? … I will tell her when the time is right, but It is a concern. It’s just that one question” (male, 16 yrs.).
**Suppression of post-disclosure feelings**
“I have never asked her, there are many questions that I cannot just confront her about it, I look at her, and I don’t know what to ask her. I don’t want to confront her with how I contracted this disease, what did she do or didn’t do? I don’t know maybe she was innocent, and it’s my dad who had it and infected her, so there are many things that I am not sure I want to confront her. So I decided to keep quiet” (male, 17 yrs.).

#### Post-disclosure coping differences

Participants reported distinctly different reactions to how they coped and expressed their emotional responses to the post-disclosure experience. A majority of participants expressed initial feelings of despair and hopelessness in the immediate post-disclosure period. One female respondent said: “Truthfully…. it was painful. It was bad!”. However, some exceptional participants reported that they coped well and appeared to accept the information. These participants reported that they did not experience strong emotional responses after learning about their HIV status.

“I knew it in 2014 when a certain doctor here told me not to be afraid, but he didn’t tell me anything else. Then when we got in, he talked to me and then told my mom to go outside, so when he told me I was not shocked because we had been taught that HIV is not the dangerous disease. After all, cancer is worse than HIV. So I didn’t panic” (male, 17 yrs.).

#### Disclosure and post-disclosure support needs for older adolescents

Respondents who experienced delayed disclosure (above 12 years) expressed the need to have a direct approach to discussions around their HIV status. During disclosure, adolescents reported a preference in shorter uninterrupted sessions that provided concise information on the chronic nature of HIV and the need to take lifelong medication. They also felt that they should have been affirmed and educated on the relatively healthy life one could live with regards to their dreams and aspirations despite being HIV positive.

Additionally, participants across time of disclosure expressed the need to continue receiving information updates and counselling sessions during routine clinic appointments. They felt that this would provide an opportunity for them to voice their concerns, questions and dynamic challenges as they emerge. Information requested included HIV prevention methods.

“I think when we come for our results, they should not make the counselling session too long. It tortures me more. They should make it short and to the point. Like she was told, it was chest problems while it was not. It’s better you tell me straight up, then I figure out how to cope with it” (female, 17 yrs.).

“My future… When you were told that you are positive, you feel as if your future is coming to an end. I wish they told me that my future is going to be bright, and I will succeed. I wish they told us more about the drug. And how long I would have to be on treatment” (female, 16 yrs.).

## Discussion

This article presents new findings regarding the disclosure experiences, perceptions of the disclosure process, and post-disclosure psychosocial needs among perinatally HIV-infected adolescents. To the best of our knowledge, this is the first paper that has attempted to document the views of older adolescents transitioning to adulthood. Additionally, this study brings new perspectives on the disclosure process by comparing the opinions of adolescents who had optimally-timed disclosure versus those with delayed disclosure. The psychosocial issues among ALWHIV as a result of delayed HIV disclosure, have been described in the literature [21–23] This paper provides a unique opportunity to document arising issues from the perspective of older adolescents when a delay in HIV disclosure occurs and recommendations towards improvements in the post-disclosure psychosocial care package during the transition of adolescents to adult care.

Overall, this study found that most participating adolescents had received disclosure between the ages of 13 and16 years. Similar to the sentiments expressed by other adolescents in other African countries, including South Africa, Zambia and Zimbabwe, Kenyan adolescents described initial distress, then shock after disclosure. Similarly, they also desired accurate information about their illness [23–26]. In these studies, adolescents described having ‘coped’ with the knowledge of their status and having adjusted well clinically and emotionally.

A new finding from our research was that even in cases when disclosure was timely, adolescents had an initial apparent positive coping response, followed by a re-emergence of negative post-disclosure reactions during late adolescence. In contrast, studies among adolescents in sub-Saharan Africa [27,23,25] reported post-disclosure initial distress followed positive coping in the immediate post-disclosure period among younger adolescents ages (11–13). These studies primarily focussed on younger adolescents, in this study, as the adolescents grew older, they experienced a re-emergence of negative emotions and grave concerns about the impact of their HIV positive status on relationships with sexual partners and peers [24,23]. The findings of our study are further supported by studies reporting the mental health and psychosocial issues experienced by adolescents living with HIV who are aware of their status. These psychosocial issues include post-traumatic stress disorder, depression, and severe anxiety [2,3].

While the psychosocial issues related to delayed disclosure are an expected finding, the re-emergence of post-disclosure negative emotions among those whose disclosure timing was optimal (before age 12), as recommended by current guidelines, was unexpected. A probable explanation for this is that most studies have focused on populations that had larger numbers of their study participants in the early adolescence (10 to14 years of age). Available literature indicates this difference in age may explain the findings of a re-emergence of post-disclosure feelings in this study, for developmental reasons. Often, there is also a higher level of self -awareness, coupled with the need to be accepted by peers in late adolescence [25,26,28]. In our study, this finding was corroborated by the requests of older adolescents in the clinical setting for additional psychosocial support as they began to comprehend the implications of their HIV status fully, particularly concerning their sexuality and relationships with peers and sexual partners [28]. Another possible contributing reason for this phenomenon may be the lack of clear stepwise guidance for the provision of long-term post-disclosure support, and the lack of knowledge regarding how to modify existing guidance to address the specific needs of adolescents who receive disclosure during mid-or late adolescence.

In this study, adolescents described experiencing re-emergent negative post-disclosure feelings during late adolescence that interfered with their adherence. The occurrence seemed to dilute the expected effect of disclosure on adherence and positive living. This finding is consistent with the literature and indicates psychosocial stressors contribute to poor adherence in older adolescents [28,13]

If not addressed, these psychosocial stressors result in poor outcomes after the transition to adult care despite the disclosure of HIV status [12].

Peer support was identified as key in the acceptance of adolescents’ HIV status. Existing literature indicates that perceived social support was a facilitator of coping in the period after disclosure of HIV status to adolescents [11]. Our findings suggest that peer influence in late adolescence was a strong facilitator in positive coping with the knowledge of one’s HIV status. Available evidence describes the crucial importance of peer influence in this period as the drive for self- autonomy and independence emerges [28,29,30] and the decisive role by peer-led interventions in providing adolescent responsive psychosocial support [30].

These findings present a strong argument for the need to strengthen the current WHO disclosure guidelines with clear, concise guidance to health workers on the content of continuous post-disclosure psychosocial care. There is also a need to address specific psychosocial care content for adolescents who experience delayed disclosure by providing comprehensive information on HIV prevention, sexual reproductive health and life skills on coping with the HIV status. This finding is in keeping with the current guidelines, which recognises that disclosure is not a one-time event [11]. We recommend that any guidance developed for health workers regarding continued psychosocial support for adolescents transitioning to adult care include a strong peer-support component.

A strength of this study is that it did not restrict itself to adolescents who had either reduced or optimal clinical outcomes as described by their viral load status. This inclusion is essential, as self-management with adherence to ART is a crucial goal of adolescent transition to adult care. A limitation of this study is that our sample only selected adolescents with perinatal infection who would benefit the most from a robust psychosocial support system during the transition to adult care due to the often long duration of ART they have already experienced as children. The experiences of perinatally infected adolescents are distinct from those youth who acquired HIV from sexual transmission. However, with a majority of the HIV infections occurring among adolescents and young people, the experiences of those with the sexual acquisition of HIV would be useful. They should comprise an important research question in future. Another limitation was that our study only focused on adolescents within urban settings and may not be generalizable to those in rural settings. The perceptions and experiences of the caregivers would also have been useful in collaborating and enhancing our understanding of the re-emerging psychological issues of the adolescents and what environmental factors were present. Due to limited resources, we were not able to include caregivers in our study.

## Conclusion

In this study, we found that post-disclosure support is a psychosocial need that extends long after the actual disclosure has occurred. As most disclosure takes place beyond the recommended age, there is a need for the current guidelines to address knowledge and disclosure of HIV status during late adolescence. The content should include comprehensive HIV knowledge, life skills training that emphasizes self-acceptance, and a focus on empowering the adolescents to undertake disclosure within relationships. Interventions focussing on adolescents’ transition to adult care should address re-emergent negative emotional responses and include a strong peer component.

## Supporting information

S1 Checklist (DOCX)

S1 File (ZIP)
