# Supplementary material for: Psychosocial needs among older perinatally infected adolescents living with HIV and transitioning to adult care in Kenya
Source: PLoS One. 2020 Jul 29;15(7):e0233451. doi: 10.1371/journal.pone.0233451 (PMC7390380; doi:10.1371/journal.pone.0233451)
Supplement: S1 Checklist — (DOCX) [file pone.0233451.s001.docx]

**COREQ (COnsolidated criteria for REporting Qualitative research) Checklist**

A checklist of items that should be included in reports of qualitative research. You must report the page number in your manuscript where you consider each of the items listed in this checklist. If you have not included this information, either revise your manuscript accordingly before submitting or note N/A.

| **Topic** | **Item No.** | **Guide Questions/Description** | **Reported on Page No.** |
| --- | --- | --- | --- |
| **Domain 1: Research team and reflexivity** |  |  |  |
| *Personal characteristics* |  |  |  |
| Interviewer/facilitator | 1 | Which author/s conducted the interview or focus group? Authors -VM, NGK, (other facilitators BK and RM) | Page 18 |
| Credentials | 2 | What were the researcher’s credentials? Authors- VM-BA Sociology, , NGK-MD.,MPH ( Other facilitators BK- BA sociology, RM-BA sociology) | Page 5 |
| Occupation | 3 | What was their occupation at the time of the study? Authors - VM-research assistant qualitative research in research organisation, NGK- PhD student (other facilitators RM –research assistant in NGO, BK- social scientist) | Page 5 |
| Gender | 4 | Was the researcher male or female? Authors -VM-Female, NGK-female (other facilitators- BK-female, RM-female) | Page 5 |
| Experience and training | 5 | What experience or training did the researcher have? Authors-VM-research methods training and qualitative research (6years), NGK- research methods, qualitative research postgraduate training, 6 years of research experience (other facilitators -B.K- research methods training two-year research experience, RM –research methods qualitative research). Additionally both the authors and additional facilitators had prior experience in conducting interviews for adolescents . | Page 5 |
| *Relationship with participants* |  |  |  |
| Relationship established | 6 | Was a relationship established before study commencement? No | Page 5 |
| Participant knowledge of the interviewer | 7 | What did the participants know about the researcher? There was a single contact with the study participants where the research objectives were shared , informed consent was obtained and the FGD/IDI conducted | Page 5 |
| Interviewer characteristics | 8 | What characteristics were reported about the interviewer/facilitator? none | n/a |
| **Domain 2: Study design** |  |  |  |
| *Theoretical framework* |  |  |  |
| Methodological orientation and Theory | 9 | What methodological orientation was stated to underpin the study?  Phenomenology- We sought to understand the experiences of the perinatally infected adolescents in the post disclosure period, their perception of the information they received and their feelings during the process and in the period that followed. | Page 3 |
| *Participant selection* |  |  |  |
| Sampling | 10 | How were participants selected? Purposively from large urban high volume high volume HIV clinics that had large numbers of adolescents with perinatally acquired HIV infection. | Page 3 |
| Method of approach | 11 | How were participants approached? Participants attending routine clinical visits were approached for their willingness to participate in the study | Page 4 |
| Sample size | 12 | How many participants were in the study? 58 | Page 5 |
| Non-participation | 13 | How many people refused to participate or dropped out?  None refused Reasons? N/A | Page 5 |
| *Setting* |  |  |  |
| Setting of data collection | 14 | Where was the data collected? At the HIV care and treatment clinic in private rooms | Page 5 |
| Presence of nonparticipants | 15 | Was anyone else present besides the participants and researchers? No | Page 5 |
| Description of sample | 16 | What are the important characteristics of the sample? Age,age at disclosure, sex and viral load outcomes | Page 7 |
| *Data collection* |  |  |  |
| Interview guide | 17 | Were questions, prompts, guides provided by the authors? Yes, interview guides were used. Was it pilot tested? Yes they were piloted | Page 5 |
| Repeat interviews | 18 | Were repeat inter views carried out? No | n/a |
| Audio/visual recording | 19 | Did the research use audio or visual recording to collect the data? Yes we did | Page 5 |
| Field notes | 20 | Were field notes made during and/or after the interview or focus group? Yes. Field notes were made during and after the interviews and focus groups | Page 5 |
| Duration | 21 | What was the duration of the interviews or focus group? FGDS-Atleast 60-90min while –IDIs lasted 30-45 min | Page 8 |
| Data saturation | 22 | Was data saturation discussed? Yes | Page 5 |
| Transcripts returned | 23 | Were transcripts returned to participants for comment? |  |
| **Topic** | **Item No.** | **Guide Questions/Description** | **Reported on Page No.** |
|  |  | correction? No | n/a |
| **Domain 3: analysis and findings** |  |  |  |
| *Data analysis* |  |  |  |
| Number of data coders | 24 | How many data coders coded the data? 2 | Page 6 |
| Description of the coding tree | 25 | Did authors provide a description of the coding tree? no | n/a |
| Derivation of themes | 26 | Were themes identified in advance or derived from the data? Both- we derived themes in advance and added themes that emerged from the data. | Page 6 |
| Software | 27 | What software, if applicable, was used to manage the data? Deedose 8.2.27 | Page 6 |
| Participant checking | 28 | Did participants provide feedback on the findings? No | Page 6 |
| *Reporting* |  |  |  |
| Quotations presented | 29 | Were participant quotations presented to illustrate the themes/findings? Yes they were.  Was each quotation identified? Yes through participants age and sex. | Page 7-14  Pages 7-14 |
| Data and findings consistent | 30 | Was there consistency between the data presented and the findings? Yes | Page 7-14 |
| Clarity of major themes | 31 | Were major themes clearly presented in the findings? Yes they were | Pages 7-14 |
| Clarity of minor themes | 32 | Is there a description of diverse cases or discussion of minor themes? Yes there is . | Pages 7-14 |

Developed from: Tong A, Sainsbury P, Craig J. Consolidated criteria for reporting qualitative research (COREQ): a 32-item checklist for interviews and focus groups. *International Journal for Quality in Health Care*. 2007. Volume 19, Number 6: pp. 349 – 357

**Once you have completed this checklist, please save a copy and upload it as part of your submission. DO NOT** **include this checklist as part of the main manuscript document. It must be uploaded as a separate file.**
