# Supplementary material for: Psychosocial needs among older perinatally infected adolescents living with HIV and transitioning to adult care in Kenya
Source: PLoS One. 2020 Jul 29;15(7):e0233451. doi: 10.1371/journal.pone.0233451 (PMC7390380; doi:10.1371/journal.pone.0233451)
Supplement: S1 File — (ZIP) [file pone.0233451.s002.zip › uploaded final PLOS/Reviewed Transcripts/FGD10.docx]

**M: Okay the first question is that I want us to talk about how you are copping emotionally, when you found out about your status, how did you cope emotionally? Did I start too deep? (laughter)**

R: Okay for me I found out about my status when I was 7, that’s when I started taking the medicine, that was in 2010

**M: 2010.**

R: Yeah.

**M: At that age were you able to understand your status?**

R: I was told by the doctors.

**M: What did they tell you?**

R: About my status.

**M: Mm, so how were you coping emotionally, how were you feeling?**

R: Okay for me it was very awkward because I was the only one who was taking the drugs in the house, my brother and sister are fine.

**M: Mm.**

R: So at first I stopped and then it reached last year I stopped like for 6 months, that’s why I was sick, I was in oxygen, I was in the hospital in and out.

**M: What made you stop? Sorry I am focusing on you only.**

R: Okay, issues home, I don’t like it when people fight at home.

**M: Mm-hmm, people were fighting.**

R: Mm.

**M: Okay, as we continue we shall return there, okay someone else? How did you know and how did you cope when you found out about your status?**

R: I was so shocked I almost fainted. (laughter) I never knew that I would have HIV.

**M: When did you find out and how did you know?**

R: A few months.

**M: A few months ago, how did you know?**

R: My doctor is the one that told me.

**M: You were not told at home, you were just taking medication, no one was telling you what the medicine is for. So how did you cope when you felt like fainting?**

R: I had to just be brave.

**M: On your own?**

R: Yeah.

**M: Okay.**

R: I found out when I was in class 3, in 2010, I was told by my dad, I was told that “there is something I want to tell you, but don’t be so shocked” I asked what? “the drugs that you take are for HIV” I said “me, I have HIV and the way I am healthy?” he told me “yes” I asked him “where did it come from?| he told me I will tell you. I said “tell me that” he told me that my mom had it, and you know my mom had died. I started taking drugs and I have never stopped.

**M: So your dad just told you.**

R: Mm.

**M: Before you took the medication?**

R: Yes.

**M: So when you were that young how did you cope, what did you think HIV was?**

R: I thought that I would die at night.

**M: At night? Sorry, you were afraid?**

R: Yes.

**M: Someone else?**

R: I found out when I was young, I can’t remember, but my mom is the one that told me, so when she told me I was not that shocked, I knew that it’s something that is there. She told me that since I have it, I have to take the drugs because they are my life. So I had to cope.

**M: How old were you?**

R: I can’t remember.

**M: Someone else?**

R: I was in class 8 that time, 2013, I was sick at that time, I went to the hospital and I was told I had no problem, so I came here, when I came here and I was admitted for one term, and I asked my mom what is the problem that I was staying in the hospital for so long. So in the end they told me my status. I didn’t accept it, I didn’t believe it, it was hurtful, I did not believe it by the way. And the way I was healthy.

**M: You asked in the hospital or you had left?**

R: I was told when I was in the hospital.

**M: And before that you were taking medication or not?**

R: No, I started when I was in class 8 in 2013.

**M: So all those years you were like that?**

R: Mm.

**M: So when you find out about status, how do you handle it, I want to feel like I was there, like I went through it, how do you feel emotionally?**

R: I felt that I was in another world, so when I look at my brother, I feel like they are in another world and I am in another world. So there are two worlds.

**M: Existing together.**

R: Yeah.

**M: Mm.**

R: But me in another world and them in another world.

**M: So the world that you are living and the one that they are living, what is the difference?**

R: The difference is that they can sleep outside anytime, and do what they feel, you see if they go to a bash, you have to carry the medication, because they can go and sleep there, they don’t have any stress.

**M: That when they will take the medication.**

R: Yeah, so sometimes I can be called to the bash, so sometimes you accept and some you refuse, like the ones you spend overnight, you have to refuse. So you are in a different world.

**M: Mm, someone else? Thanks for sharing that, how are you coping emotionally?**

R: For me.

**M: Mm-hmm**

R: For me I found out when I was in class 6, I was told by my parent.

**M: I want you to tell us, make us feel how you felt.**

R: I asked myself questions, I asked if it was me or who it was, I came to just accepted it.

**M: On your own, or there is someone who helped you to reach that place of accepting yourself or? How was it?**

R: I was told when I was in class 6, I talked to the doctor, it was painful till I cried, but I was told to push on, and my parent helped me.

**M: How was she helping you, I want to know the support that they gave you.**

R: She was telling me that I would just be fine,

**M: Mm, you took them and found out that you were fine, or you don’t feel fine?**

R: I am fine.

**M: The rest of you, how did you feel?**

R: I felt very bad, you see my brothers, and sister, I am the last born and I am the only one with HIV.

**M: And they are older than you.**

R: Mm.

**M: Someone else?**

R: For me I was told in 2013 by my mother, I felt heartbroken, I asked where I got it from but I have never been told.

**M: Eh?**

R: I was never told where it came from.

**M: You asked where it came from.**

R: Yes, I started taking when I was a baby and I have never stopped.

**M: So there are some questions that you have?**

R: Mm.

**M: That you don’t have answers to, like where it came from.**

R: Why me.

**M: Why me?**

R: And the first born doesn’t have.

**M: Okay, what other question?**

R: What did I do to deserve it?

**M: You feel like it’s your fault.**

R: Yeah.

**M: Other questions you might have?**

R: There is no cure, where can it came from?

**M: Mm, so when you are asking yourself these questions, do you ask yourself or there is someone you ask?**

R: I ask myself.

**M: You ask yourself, no one you are close to can answer those questions?**

R: They won’t answer, they will tell you next time.

**M: Next time, who can you ask apart from yourselves?**

R: Parent, doctor.

R: Parents

**M: Parent.**

R: Doctor.

**M: Doctors.**

R: Counselors.

**M: Who else? So when you are discouraged, just think of a tie=me you were very discouraged, what were you thinking and who helped you and encouraged you? Because I am sure there are those moments of discouragement, or those times have passed?**

R: They are there.

**M: Who encourages you?**

R: My dad.

**M: Your dad, you are free with your dad, how does he encourage you?**

R: He tells me that it’s for my own good.

**M: So they encourage you to drink the drugs.**

R: Yes.

**M: So what else discourages you other than taking the drugs, I know it can be hectic, or it’s for him only?**

R: It’s for everyone.

**M: For everyone right? So what encourages you to continue taking the drugs?**

R: Reducing the viral load so that it is not a lot.

**M: That is the reason you are taking, but you don’t feel like taking them. What makes you take the drugs?**

R: That was long ago, right now I take them, it’s like a duty every day.

**M: You are on duty/**

R: Yes it’s like a duty.

**M: So the discouragement is about taking the drugs every day, or there is something else that is discouraging you?**

R: It’s just that.

**M: That is what is discouraging you, for everyone?**

R: Yes.

**M: So the drugs are a challenge.**

R: Mm.

**M: So when you think about the day you found out about your status, is there a way you would want the scene to be different, say maybe I should have been told this way, I would have received better, or if you were told by someone else it would have been better.**

R: It’s just fine the way it is.

R: It’s fine.

**M: Why are you saying its fine like that?**

R: I felt it was fine because my mom is the one who told me, I was not expecting someone else to tell me, and I didn’t want anyone else to know I had it.

**M: So for you it was good that your mom is the one who told you.**

R: Yeah.

**M: Someone else?**

R: It was okay with my dad telling me, because he is the one who told me no matter what there is still a future,

**M: Okay, someone else? You haven’t talked to me.**

R: By the way it was not good for me because I started taking the drugs when I was a baby so it reached a stage where I asked why am I the only one who was taking the drugs.

**M: Mm.**

R: So the way my mom told me I didn’t like it, so I back slid a bit.

**M: How did she tell you?**

R: She told me that I got the virus from my mom.

**M: Who told you?**

R: My aunt, I am calling her my mom.

**M: Oh your mom passed?**

R: Mm.

**M: Oh she just told you?**

R: Yeah.

**M: When you were how old?**

R: I was 14.

**M: Wow, so you decided to stop using the drugs, because she discouraged you?**

R: Mm.

**M: So if you were to change the situation, how would you want it to be? If you had the power to change the situation?**

R: I would want to be told when I was like in class 3 because that is when I would have accepted well, then she would have taken me through a process where I would have accepted myself how I am.

**M: But she was not patient.**

R: Mm.

**M: Someone else? So when you found out about your status, when you come to the hospital, do you get information about medication and the importance of taking drugs constituently, at the right time? Or you are just given drugs and next appointment? Don’t worry I don’t work for KNH, I won’t tell anyone out there. When you come here for counseling, for treatment, how is it? Tell me how it is.**

R: It is good, you are advised to take the drugs, stay in the first line because if you go to the second line, it will be worse, you will get these other viruses, like typhoid. And then we are told about timing because the medicine works for 12 hours, the one that we are using, the more you keep time, the more you will be better in the future.

**M: Which other Information do you get?**

R: We are told that when we are taking these drugs, we should not take other drugs, like alcohol, smoke cigarettes because it can cause your viral load to go down. If you use those things you will be weak.

**M: What other information do you get? So all this information, are you given by the counselors or the doctors or who gives you this information?**

R: The doctors.

R: Both .

**M: Is it the doctors or counselors?**

R: Especially counselors.

R: Yeah.

**M: Especially counselors, what other information are you given?**

R: We should take the medication on time.

**M: Mm.**

R: I have a question, when you have taken drugs at 7 and you forget or you just ignore, you take 11am in the morning, what can happen?

**M: So you remember what I said, if we have questions I will write it down and then they will be answered, so that one I have written down. What will happen when you change time of the medication, what other information do you get? Just that? And is there any information that you feel that you should be told but there is no one telling you about that information?**

R: Where does the original drugs come from?

**M: The ones you are taking are fake?**

R: This is for reducing.

**M: Oh the drug that is going to heal where it is going to come from, what else would?**

R: I wonder about the old man in Tanzania, the was a time I was looking for the cure, I was staying with my aunt, she is from TZ, she told me “Mzee Babu from TZ, there is some medication that he is giving out and at the moment its HIV” so I told her “why don’t we go auntie?”. So we went, we woke up early and went there.

**M: You went to TZ?**

R: Yes, there were so many people, we made a line, the medicine was herbal, it was full in a cup, I drunk it. So drunk it and I went back home, after two years I was at home with my friends and there are guys who come to encourage us when we were in class 8. They said if we want to know our status, we should get tested. So I decided to get tested and see whether the herb that I took, I got healed. I was told that I was positive, I just left, I felt bad. So when I went back home, I went and told my aunt, why am I not healed? She said “you are healed” I said not yet, she asked “who told you that you are not healed?” I told her that we were counseled at school and when I was tested I was told that I had it. She said “that doctor is fake he has lied to you” after that we just quarreled and I just told to just stop, because I already have it and it is never going to end. She told me “I am already healed but just stay there if you think you are not” she had TB but she got healed. So after that a friend of hers who had HIV, we heard after 3 months that she had died.

**M: She had also gone also to Babu?**

R: Mm.

**M: So after you went to him, you stopped taking the drugs for the hospital?**

R: Yes, my aunt told me to throw them in the toilet, and I did.

**M: Wow.**

R: So I went to class 8 without taking them, so I started taking them in form 1 at second term.

**M: So does your aunt know that you are taking them, are you still living with her?**

R: No I am staying with my dad, I won’t go back to TZ< she told me my faith is very little, I told her no. she is a good aunt because she would have told everyone that I had it. I like her because she doesn’t want people to know.

**M: And when she was taking you there, she had faith that she wanted you to heal.**

R: Yes, she said we would be healed, but her Tb healed.

**M: But TB heals, so she stopped taking the TB medication?**

R: She stopped, she threw all of them in the toilet.

**M: Wow, okay, that is a challenge, so when you are being given this information, are told about sexual and reproductive health information?**

R: Yes.

**M: And you haven’t told me that you are told, so you are getting that information, from counselor or doctors?**

R: Both.

**M: What information do you get about sexual and reproductive health?**

R: Stay protected.

**M: What does that mean?**

R: Use protection during sex.

**M: Use condoms, what else? Contraception, are you given such information?**

R: No.

**M: About marriage and children?**

R: You are the one who is going to choose whether you are going to get married or not. I won’t,

**M: Why?**

R: Because you might transmit it to your children.

**M: So you will decide if you will stay single?**

R: Mm.

**M: You will stay single all your life?**

R: Yes.

R: I asked the doctor when I came to the clinic, I asked him because I am positive I will give birth to children who are positive, he told me that I won’t get children who are positive, that is what he told me.

**M: But you had to ask, it is not information that you were given.**

R: Yeah I asked, because that was bothering me.

**M: Okay, those are things that you think about? marriage and kids?**

R: Of course

R: What if you are positive and your boyfriend is negative? What will happen?

**M: What do you think will happen?**

R: As long as you have protection, there is nothing that is going to happen.

**M: Okay, we will answer all those questions.**

R: I ask myself, now that I am positive and I have a girl, so and I want to propose to her and you have to tell her your secret, so when you tell her that I am HIV, what do you think she is going to react? She will leave you.

**M: She will leave you?**

R: She will leave you.

**M: So you give up before you think about that story.**

R: Yes.

R: I will pray for people.

**M: Your job will be praying for people?**

R: Yes.

**M: You don’t think you deserve to have a family?**

R: Yes.

**M: Okay, what were you saying?**

R: I have a girlfriend, I approached her, and I told her everything, I dropped her some lines, and after that I told her that I was positive. So you know if we were meant to be together we were meant. She had no problem, she said she would die for me

**M: Wow.**

R: So I felt that I was lucky, so this thing about my status doesn’t bother me because I have someone who is caring, she has no problem with it.

**M: Okay.**

R: I told her early, the mistake would be not to tell her early. If you tell her late she will be heartbroken, she will just leave you there.

**M: Do people have courage to disclose their status?**

R: For some people.

R: You have to examine

R: Friends, boyfriend no.

**M: Boyfriend no.**

R: Yeah.

**M: It’s not everyone.**

R: Yeah.

**M: Who do you disclose?**

R: Uncles, aunts.

**M: So he is the only one who has disclosed to his girlfriend.**

R: No.

**M: Why?**

R: I can tell her and she will go and tell everyone, I will feel bad when everyone will know, that is not good.

R: Your self-esteem will go down and all

**M: So you have to protect yourself early, but you are the brave.**

R: I also have her secret and I would not tell, so if she breaks mine I will break hers, but that won’t happen she won’t tell others.

**M: So the people we are comfortable to disclose to is the family members?**

R: Not all of them.

**M: Not all.**

R: Parents.

**M: So is there a time you said there are times you don’t feel like taking the drugs, what makes someone not want to take the drugs?**

R: Getting bored.

**M: Getting bored, what bores you, isn’t it just taking drugs?**

R: There are some which are, especially the ones for night.

R: So big.

**M: So size.**

R: Not the size, there are those when you take them you become weak, you can’t even walk.

**M: They are strong.**

R: You sleep and you see things that are not there.

**M: Eh.**

R: You can’t even walk.

**M: You remember that you have to take tomorrow, you are bored.**

R: Yes.

**M: Are there those people who are taking twice a day?**

R: Yes.

**M: How does it feel when you take twice a day?**

R: Eish, wee, you wake up in the morning and take the drugs, you are told to eat and then at night you take them, after you eat/

**M: Eating is the issue.**

R: In the morning when you are sleepy you are told to wake up and eat.

R: And you are told to eat.

**M: You don’t like to eat?**

R: I would eat very well at 8am, but 6am, you are told to eat, maybe school days.

**M: Okay, and how is your experience?**

R: You are waken up at 6 in the morning to take drugs with cold water.

R: Water is not even passing through you mouth.

**M: You take the drugs with?**

R: And at that time it is not one, they are like three.

**M: Mm.**

R: you just throw them in your mouth.

**M: You drink with water?**

R: Water? No juice.

**M: And when you are in school, where do you get the juice?**

R: You ask someone to give you.

**M: Is there a difference, for those who are in boarding schools, is there a difference?**

R: There is a difference.

**M: What is the difference?**

R: You have to hide while you are taking the medicine.

**M: You hide.**

R: All the time you are hiding.

**M: When you are in school.**

R: Yes, like when you are in class and the teacher is there, who is going to let you go?

**M: No one, so you have to carry. Other challenges when you are in school? Many of you are in boarding or day, which is better?**

R: Boarding

**M: Boarding is better, why?**

R: Boarding is better.

**M: Why?**

R: The principal is good, he treats us like a father, and the matron knows.

**M: They know?**

R: Yes.

**M: Are all schools the same?**

R: It depends on the matron that is there.

**M: So when you come, how will the Principal know that you are positive?**

R: The parents will tell him.

**M: It’s the same for everyone or there is a different situation>**

R: I was in day school

**M: How was the experience?**

R: I would drink at home in the morning and evening.

**M: So you were close to home?**

R: Yes.

**M: So you did not have to worry about the jam or anything like that, or a game and you get late.**

R: I did not like it, you might be taking the drugs and someone gets in, maybe its 6pm you are back home and then you are trying to take the drugs, what will you do?

R: To tell them to mind their business.

R: You tell them you are not feeling well.

R: Yes.

**M: So apart from how the drugs bore you and it makes you weak and its big, are there other challenges that you get, so for instance that you have gone to visit your auntie or friend, and then you get late, does that happen?**

R: When you are go to your aunt, you can’t spend the night there, it’s hard, but if there is a bash, you keep the drugs in the pocket, or somewhere that is safe, when time comes, you go and take.

R: Sometimes you can’t stay in a party, you have to leave when the event is starting, because its 6pm and you haven’t taken the drugs, so you leave the party.

R: I drink the next day.

**M: There is no one who skips taking them, you skip like 3 days?**

R: That is like asking for death.

**M: You never skip?**

R: Maybe just in the morning, if you get sleepy you sleep.

R: You set an alarm.

R: You don’t hear it sometimes.

**M: Is there someone who has skipped for some time? Even there are some who throw in the toilet. Has it ever happened to you? You told us there is a time you stopped using, someone else?**

R: I stopped using for two weeks.

**M: Did someone know?**

R: Yes.,

**M: Who?**

R: My dad

**M: How did he know?**

R: He found me throwing.

**M: He asked you?**

R: Yeah.

**M: What made you stop?**

R: Just bored with the drinking, I wake up in the morning and take them, I got bored.

**M: Mm, there is no one who stopped because you wanted to be like other persons?**

R: Yeah.

**M: So boarding and day which is better?**

R: Boarding.

**M: Why is boarding better than day? I want like two three reasons.**

R: Those who were in boarding should answer.

R: There are those of us who are not in boarding.

**M: Mm, those who said boarding are who?**

R: There are those who are in day school and there are those who are in college.

**M: By the way, how is the experience of college?**

R: Okay taking drugs is not hard because all my friends know, so sometimes they are the ones who wake me.

**M: To take them?**

R: Yes.

**M: Why did you tell them?**

R: They are people that I went to school since I was young.

**M: So they have known you from far.**

R: Yes.

**M: Mm, and you trust them like family.**

R: Yes.

**M: Does it help you when you have people who can support you, those who won’t judge you and encourage you to take your medication or? Or you would rather be private.**

R: You should just die alone.

**M: You die alone, you don’t want to involve someone else?**

R: Yes, there is no good person.

**M: What is the worst thing they can do?**

R: You can’t know.

**M: In your imagination?**

R: Yes.

**M: You can’t trust people**

R: Mm.

**M: What are they going to do?**

R: They will tell others.

**M: So talking to others, that affects you.**

R: Yes.

**M: Another reason you can’t trust people?**

R: They will spread.

**M: And for those who have disclosed, if you have disclosed to your friends, how is the experience?**

R: It has been good, because there was one time, I was not feeling good, I was in a party, and when I went to bed I was not breathing well, so she had to call my mother, so I was rushed to the hospital and mother came later on.

**M: So them knowing your status helped you/**

R: Yes.

**M: Okay someone else? I want different opinions, disclosing or not, what do you prefer?**

R: Not disclosing.

**M: Why?**

R: Because you might disclose and then they leave you, some may tell others that you have HIV, so I would rather keep it.

**M: And you?**

R: What he said.

**M: You agree.**

R: Mm.

**M: You too?**

R: Yes.

**M: What is your opinion?**

R: You disclose to people that you know, the few that you trust.

**M: But not everyone, friends can you tell them>**

R: Yes.

**M: There are those that know?**

R: Yeah.

**M: How do they support you?**

R: They know how I take my drugs, so if I have not woken up, they call my mother, or they call me and remind me.

**M: So it seems the girls talk more the boys.**

R: We are don’t cares., being reminded it’s like winning a million, you are not reminded.

**M: You have to remind yourself, you should know when you are supposed to take the medication.**

R: Yes.

**M: Okay, so say do you have parents or any relatives that are HIV positive?**

R: Yes.

**M: Do you feel that they support you or how do you interact with them?**

R: They support us.

**M: How are you related? If you are free to say, how do they support you?**

R: We remind each other the appointment, sometimes my mom reminds to take drugs.

**M: Okay what else? What is your experience, or there is nothing else, or you don’t want to say we pass?**

R: Next question.

**M: We skip it or there is someone who wants to add?**

R: No.

**M: Okay, so when you are home, do you feel supported and encouraged by anyone?**

R: Not all the time.

**M: Tell me more.**

R: Not everyone in the family knows, only my sister knows, my brother is in boarding, he knows a bit but not so much, but my sister knows.

**M: A little is? Does he know you are HIV positive?**

R: No, he doesn’t know he just knows I go to the hospital , so when it is time to go to the hospital, I call my sister to go with me.

**M: Because there is no one else that will take you/**

R: Mm.

**M: So how does that make you feel?**

R: Bored.

**M: So your brother doesn’t know, he just knows you go to hospital.**

R: Yes

**M: You said not so much, let’s talk about this, so that we can bring change especially in support.**

R: They support financially also.

**M: They give you.**

R: Yes.

**M: There is no time you feel you are treated differently because of your status, or you are pampered because of your status?**

R: Yes.

**M: It’s there, how does that make you feel, good or bad?**

R: Good.

**M: Good.**

R: But it reaches a point where your brother or sister starts to complain.

**M: Why you are treated better.**

R: Yeah.

**M: Mm, so when it gets there what do you do?**

R: That is life.

**M: Will your sibling have a grudge with you because of it?**

R: My mother favors me a lot and my brother feels bad, my dad loves my sister more, and so when my dad is not there, my mom will go out and buys chips and brings me.

**M: What do they say? They think you are loved more?**

R: When they are brought, they won’t give you.

**M: How are treated different?**

R: I am treated well.

**M: How what are you given others are not?**

R: Financially.

**M: You are given more money than the others?**

R: Yeah, pocket money.

**M: Do they know you are given more than them?**

R: Yes.

**M: And they don’t complain?**

R: They cant.

**M: Why not?**

R: They know.

**M: They all know your status?**

R: Yeah.

**M: So they also support.**

R: Yes.

**M: Okay, someone else, there is no time you are treated different because of your status in the family?**

R: No.

**M: Okay, so we are about to finish, are you bored? So if you think about your life, there are people here in college, and high school, when you think of your life beyond college, and beyond high school, what do you think? Do you feel after you finish high school and college, do you think you will succeed with your dreams and hopes or what do you think?**

R: I think I will succeed, I just believe and I know god will not let me down.

**M: Someone else?**

R: I think it will be okay, if we push on, we will succeed.

**M: Mm.**

R: I have nothing to say.

**M: You have nothing to say or you don’t want to tell me, or you haven’t thought beyond college?**

R: It will be good.

**M: What does good mean?**

R: I am loading.

**M: In the future what do you think?**

R: I think my future is bright, as long as I work hard on my studies.

**M: Mm-hmm**

R: I think it will be fine as long as I put god first.

**M: That will be good, how long do you think you will live?**

R: The ones that god has planned.

**M: The ones god has planned.**

R: You can die any day.

R: God is the one that has planned.

**M: But when you are planning, do you see yourself with grandkids?**

R: Yeah.

R: Mm.

R: I have a nephew.

**M: Do you see yourself with grandchildren?**

R: No I don’t see.

**M: Do you see your future children, or you don’t want to look that far?**

R: Mm.

**M: It’s a hard question? Do I leave it?**

R: Yeah.

**M: Okay, so what challenges do you think you will face, you are finished with school, you are looking for work, a mate, what challenges do you face?**

R: Looking for work, looking for the course that you want.

**M: The course that you want after graduation.**

R: You have to plan with your life.

**M: Those are what I want.**

R: You decide your career.

**M: I want to hear your hopes for the future, what do you aspire to do in the next 10 years?**

R: I hope the more I am taking my drugs right, I can be even a role model, some generations I come here and tell people how my life is.

**M: What other hopes?**

R: I want to be a scientist, I want to look for a cure for this thing so it can stop bothering us.

**M: Right! Someone else, what are your hopes that we have?**

R: Reduce the spread.

**M: Okay reduce spread, hopes for the future? Aspirations? There are none? So I want to ask a question, have you ever experienced stigma, where someone has treated you differently because of your status? Is there anyone who has ever experienced or there isn’t?**

R: There isn’t.

**M: The two of them have told me there isn’t, the rest of you?**

R: None.

**M: Okay, the way you have looked at me I am just asking myself questions, is there anything that you would like to ask, something that we have not covered?**

R: These drugs that are new, they are called Pepes, what are they?

**M: Okay we will answer that. Another comment?**

R: I have a question.

**M: Mm-hmm.**

R: Will I take those drugs till I die?

**M: Mm. someone else?**

R: I heard that when you are HIV positive and your viral load is low and then you have sex without a condom or anything, you can’t transmit HIV, is that true or not?

**M: Mm-hmm someone else?**

R: When your viral load is low, and you go and check your status, will you be positive or negative?

**M: Someone else?**

R: I have a comment.

**M: Mm.**

R: This hospital doesn’t take us anywhere far, we don’t go somewhere we can be trained by someone, like going to a camp or seminar.

**M: It would be good?**

R: Yeah, having entertainment, dancing, socializing

**M: Right?**

R: Yes.

**M: Mm-hmm is there another comment? Let us reach there, thank you so much.**
