# Supplementary material for: Psychosocial needs among older perinatally infected adolescents living with HIV and transitioning to adult care in Kenya
Source: PLoS One. 2020 Jul 29;15(7):e0233451. doi: 10.1371/journal.pone.0233451 (PMC7390380; doi:10.1371/journal.pone.0233451)
Supplement: S1 File — (ZIP) [file pone.0233451.s002.zip › uploaded final PLOS/Reviewed Transcripts/FGD2.docx]

**FGD 2**

**M: By the way, which language are we comfortable with?**

R: All of them.

**M: All of them, Kikuyu?**

R: Swahili and English.

**M: Swahili and English, we can mix.**

R: Yes.

**M: Okay, so I want us as we are starting our discussion, I want you to tell me how you cope emotionally with the knowledge of your HIV status, every day when you wake up, how is your life, how do you cope? You don’t have to be the one to answer, I am just smiling and pointing, who wants to start? When did you know your status and how did you find out?**

R: I found out when I came here, I was taken to a certain room and I was asked some questions and I was told that I have HIV.

**M: When was this?**

R: 2014.

**M: Were you on your own?**

R: No I was with my mom.

**M: She is the one who brought you?**

R: Yes.

**M: She is not the one who told you about your status?**

R: She is not the one.

**M: Who told you?**

R: The doctor.

**M: How did you feel being given news like that, that you have HIV?**

R: I did not feel bad, I accepted.

**M: Mm-hmm, someone else?**

R: I was told by my step mother, she told me in 2013.

**M: 2013**

R: I had questions but she answered me.

**M: You had questions like?**

R: How? She answered but I had to accept.

**M: What made her tell you?**

R: Because I got late coming home one day and that is the day that she told me.

**M: You got late coming home?**

R: Yes, she told me.

**M: She was mad or?**

R: No.

**M: I want to know the relationship between being late and telling you.**

R: I got late taking the medication.

**M: So she explained to you why had to take them.**

R: Yes.

**M: Okay, someone else?**

R: Me?

**M: Mm.**

R: When I was in school boarding, I used to get sick a lot and my dad was told to come for me, we went to a hospital in Rounda, I was tested, that day he didn’t tell me, we stayed a while and then about two years, he told me.

**M: So when you were tested, were you given any medication or?**

R: Yes I was given.

**M: You were not told what the drugs were for?**

R: No.

**M: So what made them tell you?**

R: He is the one that started.

**M: How did he start, did he say “sit here young man, there is something that I want to tell you” or how did it happen?**

R: No, I went to the countryside, so when I was there, he left me there, and then I was told by my grandfather. He asked me if I knew why I take drugs. I said I don’t know, and then he told me.

**M: How did you feel?**

R: I did not feel bad,

**M: You were normal?**

R: I had to accept it.

**M: Okay thanks for sharing, someone else anyone.**

R: I knew it in 2014, when a certain doctor here told me not to be afraid, but he didn’t tell me anything else. Then when we got in he talked to me and then told my mom to go outside, so when he told me I was not shocked, because we had been taught that HIV is not the dangerous disease, because cancer is worse than HIV. So I didn’t panic.

**M: So you were on medication before that?**

R: Yes.

**M: Oh already you were on medication.**

R: Yes.

**M: Mm-hmm, is there anyone else who wants to share?**

R: I started taking the medication when I was a baby, I tool them till I was in class 5, I used to come late and then my aunt told me that I should not get late because of the medication that I need to take to go on. I asked her why, what are they for, and she told me that she is not the one who is going to tell me. When we went to the clinic, we went to a certain doctor, Dr. Christine. We were tested and I was told I had it, I did not take it hard because I was taking the medication from long ago. I continued to take them.

**M: So you were with your auntie at the time or until now?**

R: My auntie till now.

**M: Okay.**

R: I had been drinking for a long time and then one day while we were walking my dad told me. I didn’t panic because he encouraged me and told me that if I took the medication, nothing bad will happen to me. So I just continued with the medication.

**M: What age were you when he told you?**

R: I was 13 years.

**M: Mm-hmm.**

R: I found out in 2014, and my mom is the one that told me because I was bothering her asking what the medication was for, she told me that it was for HIV, and I didn’t take it too hard, I just continued taking them.

**M: Mm.**

R: I found out 2015, I was coughing a lot in school and then my mom brought me here, and when I was tested they found out that I had it. I took it normal, I didn’t panic.

**M: You guys are strong; here there is no one who panicked.**

R: I was shocked at first, and then I was encouraged that it’s not bad.

**M: So you were a bit shocked.**

R: Yes.

**M: So after knowing your status, every day you wake up, you take medication, you are telling me that life is still normal, there are no challenges that you face, at some point because everyone its normal, even for someone without HIV, sometimes you think about something and you feel discouraged, sometimes you are feeling encouraged. So I want to hear about those moments where you feel discouraged. And what do you do? You can’t tell you are strong all the time, there is no one who is encouraging you, you are the only one encouraging yourself. I know you are men, you are taught you have to be strong, but everyone has that moment when they are discouraged, I want to understand that experience, at your age you are going to school and you are taking medication every day, who has the courage to tell us? And when you are discouraged, what do you do, I just want to understand. Talk to me.**

R: Taking medication all the time, I wonder why I have to take drugs all the time, in the morning I wake up to go to school, I have to take medication, sometimes I am weak.

**M: The drugs make you weak?**

R: No, I am just weak, and my auntie tells me to continue drinking them, that one day they will give one drug.

**M: She encourages you.**

R: Mm.

**M: So at least she encourages you, so taking the drugs is a challenge.**

R: Yes, in the morning, and at night, it is boring, before you sleep you have to take them, in the morning you have to take them.

**M: So what encourages to take the drugs every day or who is there someone who encourages you?**

R: My mother is the one who encourages me, she tells me that, it gave me back life, so I should just take it.

**M: It brought you back life, there was a time you were sick?**

R: Yes..

**M: Mm.**

R: So she was telling to just take them.

**M: Mm, but there are times when you feel you don’t want to take them.**

R: Yes.

R: Of course.

R: It is discouraging because it takes my time which I have planned and I have to take them, so I have to postpone the plans and it makes me mad doing the same thing every day. But my mom encourages me.

**M: It takes your time for what say when you have planned for what?**

R: If I was to go somewhere, and time is almost there I can’t go.

**M: Because you might be late, you can’t carry them?**

R: No.

**M: Why?**

R: Because of the bottles.

**M: So you would rather take and then leave, so it ruins your schedules.**

R: Maybe sometimes my friends keep asking me these medications that I keep taking, what they are for, and you wonder what you are going to say, and then my mom tells me to continue with them, they can make my immune be strong, so she advices me to continue using it.

**M: So how do your friends know that you are taking medication?**

R: They come home.

**M: So they find you talking the medication?**

R: Yes.

**M: So its time, you have to take the medication, you have to take in front of them?**

R: Yes.

**M: Okay, that can be a challenge to explain.**

R: Sometimes I tell my friends that it is panadol.

**M: Mm-hmm.**

R: I tell them it is for my chest.

**M: They just let it be.**

R: There are some who say that the medication looks too big and looks like it is for HIV and I just shrug it off.

**M: So you are discouraged when you see your friends looking at your life?**

R: Yeah.

**M: So what do you do, do you cut down your social life or there is someone who you speak to who encourages you?**

R: Yes

**M: What do you do?**

R: I just console myself.

**M: How do you console yourself?**

R: This is the life, so I have to continue.

**M: Mm, so you tell yourself this is the life.**

R: Yes.

**M: Okay, someone else? There is no one who is usually there to encourage you when you are discouraged? Maybe your aunt talk to you or your father talks to you? Is there something else they do to encourage you? Maybe they give you like 200 to buy chips? Or they don’t do that?**

R: They don’t do that, they can’t do that.

**M: What do they do when you are discouraged?**

R: They just give me time and then give me the drugs to take.

**M: They just give you time to calm down.**

R: Yes.

**M: Okay someone else?**

R: Maybe they call me, and talk to me and then they ask me what gift I want for my birthday, so they buy for me and they know that you are happy.

**M: Mm-hmm.**

R: She takes me somewhere to visit and we sit and talk.

**M: It helps you lift your spirit.**

R: Yes.

**M: Okay so most of you told me that you found out about your status in the hospital apart from you, you told me that your grandpa told you. So when you went to hospital and you were told that story, what information did they give you about the drugs? Is there any information you were giving, or you were just told you were HIV positive, how was the process? Or you don’t want to revisit that process, because I know it is not easy, how did it go, when you came to the clinic, what information were you given?**

R: They talk to me about nutrition, they tell me the foods that I am supposed to eat, the fruits, greens.

**M: So you are given information about nutrition.**

R: Mm.

**M: Someone else?**

R: You are told not to stop taking the medication.

**M: Why? Imagine you are the doctor, and I tell you “these drugs are boring me”**

R: They will tell you to just continue because they are the ones that add strength to you.

**M: Okay, what if I miss the appointment, maybe my appointment was the 29^th^ and now it is 3^rd^ of the next month.**

R: You are encouraged, that if you don’t take then this and this will happen.

**M: What will happen, why don’t you tell me?**

R: You will become weak and reduce your life, and so you are encouraged and be told what you are going to do.

**M: Okay, how does it go, is there information you wish you were told when you come to the clinic? Are there questions that you wish you could answer, questions that no one answers? Or there is nothing you can answer because you don’t know if you will be answered? So you are just told to take medication and nutrition, that is all?**

R: I am told not to lose hope, I will be cured.

**M: You are told you will be cured?**

R: Yes.

**M: What else?**

R: I ask them if they can take all that medicine and put them somewhere and make them one drug.

**M: Mm, you take them once?**

R: Maybe if its morning and evening, just once.

**M: How many do you take?**

R: 4 at night and 5 in the morning.

**M: Wow, those are many, and you manage to take all of them?**

R: Yes.

**M: So it’s better if it’s one.**

R: Mm.

**M: I hope it reaches that point where you can take one per day, because there are some people who take one per day right?**

R: Mm.

**M: Other questions that you might have? There are none? You just come and what you are given you take and leave. I am sure there are those questions that you want to ask, when you are alone you ask yourself some questions, this is a good important opportunity to ask. And this is not a counseling session it’s a discussion. What other information do you get? Are told anything about sexual and reproductive health?**

R: Yes.

**M: You are told, why have you not remembered? Everyone or its just you?**

R: I am told so I don’t know if they are told.

**M: Are you told?**

R: Mm.

**M: What are told?**

R: I am not told.

**M: Why do you think you have not been told?**

R: I don’t know.

**M: Okay, the rest? Contraception, are you told about this?**

R: Yes.

**M: What do they tell you?**

R: They tell me if I want to have sex I should use a CD.

**M: Mm, someone else? Why are you looking at him like that? What are you told?**

R: They tell us that we should not have sex when you are not married.

**M: If you are not married.**

R: Yes.

**M: Is that possible?**

R: It’s possible.

**M: Okay, what else do they tell you, they don’t tell you about other infections like STIs, you are not told about this?**

R: We are told.

R: I have been told.

**M: You have.**

R: We have been told that when we have sex with someone who has HIV and you have HIV, the virus is not the same, you can get what is bigger.

**M: Some other information? And is there information that you wish you were told?**

R: Yes.

**M: Like?**

R: Like when you are an adult and you get married, can you get a child that is not HIV positive.

**M: Do you get such information?**

R: Yes I am told.

**M: You are, what do they say?**

R: You can get a child who is not positive if you don’t breastfeed them.

**M: Mm.**

R: But if you breastfeed them, they will get it.

**M: Is that the information that all of you have been given?**

R: Yes.

**M: All of you?**

R: Mm.

**M: There is nothing else you are told, that is all? And marriage, are you given any information about married and having a family?**

R: I haven’t heard of it yet.

**M: You haven’t been told.**

R: Yes.

**M: And is this information that you would like to get?**

R: Yes.

R: Yes.

**M: Okay so what would you like to know about married and children?**

R: How you can live with your wife and the children not get infected.

**M: Okay, and what else?**

R: How you can get a family that is negative.

**M: What else? Do you reach a time you don’t want to take those pills, that you are tired of them?**

R: Yes.

**M: That’s normal.**

R: Yes it’s normal.

**M: What situation would make you want to feel that you are tired of taking the pills?**

R: Because they are big.

**M: When else? Would you quarrel with your parents or someone and then not take the pills?**

R: Yes, there was a time I quarreled with my mom and I refused to take them, but then she talked to me with kindness and then I took them.

**M: Okay, and someone else? When else have you felt that you don’t want those drugs.**

R: When friends have visited and I need to take the drugs, when you take them, they start asking questions, and those questions are what I am avoiding.

**M: So you avoid those questions.**

R: Yes.

**M: So you prefer not to take them at that time?**

R: No I hide and take them.

**M: When else do you feel you don’t want to take the pills?**

R: Sometimes I don’t want to take them with water, I want to take them with tea and drinks, so if it is not there, drinking with water is hard, but I just drink it.

**M: So taking with drink is better.**

R: Yes.

**M: How does that help?**

R: I don’t like water that much.

**M: Especially in the morning.**

R: Yes.

**M: Okay someone else, when else do you feel, is there someone can say that they don’t want to take them because of the side effects that they get? How the medication makes them feel, would that make you not want to take them?**

R: It can.

**M: And if you are in school, how many of you are in school here? High school, primary, collage.**

R: I am going to high school

**M: Are you in boarding or day?**

R: Boarding.

**M: Most of you are in boarding?**

R: Yes.

**M: Is there someone in day?**

R: Yes.

**M: What challenges do you get when trying to take your medicine?**

R: Maybe you have reached home late when the time has passed, I try to leave early so that I can reach home early.

**M: What can make someone get late coming from school?**

R: Maybe in school there was ball in school, so you have to.

**M: You have to hang around till it’s over.**

R: Yes/

**M: Traffic jam or you just walk going to school?**

R: Yes.

**M: Okay and boarding what challenges do you get there?**

R: Maybe time has reached and the teacher refuses to let you go take your medication, they tell you to wait for the lesson to end.

**M: Mm.**

R: So you have to wait, when I tell him that I am sick he tells me to wait.

**M: So when you are going to class, you have to carry your drugs when you are going to class or?**

R: Sometimes I forget to carry.

**M: And when you forget, are they in the dome or?**

R: They are in the dome.

**M: And is the dome open at all times?**

R: No.

**M: So what happens when you forget to carry?**

R: You ask for permission and then you go and take them.

**M: Always will he allow you to go and take them.**

R: Yes, but then you have to go and take water.

**M: Okay and what other challenge is there in boarding?**

R: When there is an activity and you have to ask permission from the teacher to take medication.

**M: And when you ask the teacher for permission for taking pills, do they ask what the pills were for?**

R: They ask.

**M: What do you say?**

R: I say it’s for the weather.

**M: How?**

R: So that weather does not affect me.

**M: And does the teacher believe?**

R: You know they have to doubt sometimes.

**M: So everyone who is in boarding you keep the medication yourself or how is our experience? Or there is someone that is…**

R: I keep for myself.

**M: There is no one who has a matron who keeps for them or a school nurse?**

R: Matron.

R: Nurse.

**M: And have you had a situation where you have changed medication for the time that you are drinking because from what I hear is that when you are taking medication maybe that time of school, have you ever considered changing, that you drink the time for class and drink time when it is not class time?**

R: But I look at a time when I am off, and the time I won’t be off, so when I decide it’s like 6am, I can be drinking at that time.

**M: Okay so for those who are in boarding or day, when the schools are closed and when they are closed, is there a difference in the way that you drink pills.**

R: Because I drink at 9am, sometimes I find that I have woken up at 9:30.

**M: When the schools are closed.**

R: Yes, so I delay taking the pills.

**M: Does that happen often?**

R: Not all the time.

**M: Okay, someone else?**

R: For me sleep is what makes me wake up at 10am and I have to take the pills even if I have passed.

**M: What is your time?**

R: 7am.

**M: And you sleep until 10am?**

R: Yeah.

**M: People sleep when the schools are closed.**

R: Yes.

**M: So when do you get late taking your pills the most, when the schools are closed or when they are open?**

R: When the schools are closed.

**M: Because of sleeping, or there are other reasons?**

R: Maybe you have gone with friends and you forget to come back home to take the pills.

**M: You forget, what other reason? So school days are better, of the schools are closed, especially December, the activities are many right?**

R: Yeah.

**M: So how do you deal with those challenges? Is there someone who reminds you? Or it’s purely your responsibility to remember?**

R: At times we are reminded.

**M: By who?**

R: By my mom.

**M: Okay someone else?**

R: Alarm.

**M: Alarm.**

R: Brother.

**M: Okay.**

R: I am just reminded.

**M: By?**

R: My mom.

**M: Okay.**

R: I put an alarm.

**M: You use an alarm, so sometimes if you are not reminded you don’t remember, the time flies by.**

R: Yeah.

**M: Okay, and do you feel that, do you have family members that live with HIV, or someone who has HIV but you interact with them closely?**

R: Yeah, my auntie and my cousin.

**M: Are you close with them?**

R: No.

**M: You are not, you just know?**

R: They come here, I meet with them.

**M: And at home, you don’t support each other?**

R: You know their home is far, they live in Mathare, and our home is Kawangware.

**M: Someone else?**

R: My dad.

**M: At home**

R: Mm.

**M: Someone else?**

R: My mom.

**M: So when you compare your relationship with them, are you more comfortable talking to this person or there is another person who you are more comfortable to talk about your status with?**

R: My dad.

**M: Why?**

R: Because he is the one who understands me more than my mom.

**M: Okay, someone else?**

R: For me it’s my mom, she is the one that takes care of it.

**M: Someone else, who are you comfortable talking with about your status, or any other thing? There isn’t?**

R: Yeah.

**M: You just keep it to yourself?**

R: Yeah.

R: My dad.

**M: Why him?**

R: He understands.

**M: Okay**

R: None.

R: My mom.

**M: Why?**

R: She understands me.

**M: Okay.**

R: My mom because she has it also.

**M: Have you ever told someone else about your status apart from your mom who you are close to?**

R: No I have never.

**M: Is there someone who has ever said?**

R: No. .

**M: Why?**

R: It’s hard.

**M: It’s hard.**

R: You don’t know how someone will take it.

**M: Another reason is?**

R: Because you can tell someone and they go telling others and when you meet with them, they just start talking about you.

**M: Mm.**

R: So you keep it to yourself.

**M: Okay another reason that makes you keep it a secret?**

R: If you disclose everyone will know.

**M: Another reason? Okay so this story of disclosing is not easy, and in a relationship, say you have a girlfriend, can you tell them?**

R: I can’t.

**M: Why? What will happen?**

R: They will start telling people.

**M: But that is your girlfriend.**

R: But they won’t understand or when you break up, she will revenge by going telling your friends.

**M: Mm, so when you are together she might keep, but when you break up they will go telling others. Another reason you can’t tell your girlfriend?**

R: Because I can’t trust people, I just trust myself.

**M: Okay, girlfriend why not?**

R: I can’t.

**M: Why?**

R: I can’t tell her because she might leave.

**M: So disclosure that is your story even if you are in a relationship, that is for you only?**

R: Yeah that is my story.

**M: Okay, fine, so do you when you look at your life, would you say you are ready. I want you to think about your life after school, do you think you are ready to face the world with your status and succeed in your life?**

R: No.

**M: Why?**

R: I am not ready to tell anyone my secret, I don’t know who I will tell.

**M: You can’t tell anyone.**

R: Yes.

**M: So when you think about your future, how do you feel your future is like? Where do you see yourself in 10 years, 20 years?**

R: I don’t think this disease will stop me from living my life.

**M: Okay someone else, where do you see yourself 20 years from now?**

R: I feel my life will be okay.

**M: How will it be, what job will you have?**

R: I would want to be a doctor to look for a cure for this disease, we can’t live just taking ARVs.

**M: How do you view family life?**

R: I haven’t thought about it.

**M: Where do you see yourself 20 years from now?**

R: I will have finished school, I will be an engineer.

**M: Career will be good, family life how will it be?**

R: Kids, wife.

**M: Okay and you, how do you see yourself in 20 years from now?**

R: I will be an architect and happy with my family.

**M: Mm-hmm.**

R: I will be an electro engineer with my family.

**M: Where do you see yourself?**

R: Being a lawyer.

**M: Being a lawyer and what else?**

R: I don’t know.

**M: Okay.**

R: I will be a doctor and live a normal life.

**M: What is a normal life?**

R: Just living with the virus.

**M: Mm.**

R: I think I will go far, even if I have the virus, I think my opportunity is still there.

**M: Mm-hmm**

R: I will be working.

**M: What do you think of family life?**

R: When I get there is when I will think of it.

**M: When you get there? You haven’t thought of it.**

R: Mm.

**M: Okay, so would you want to get training to help you feel that you are ready to face life and succeed?**

R: Yes.

R: Yes.

**M: When you hear of such training, how would you aspire it to be like, what would you like to be taught there? Is it a hard question?**

R: I don’t get it.

**M: What component should be there, what information or skills would you want to acquire?**

R: How to face life\s challenges.

**M: Be taught to face life challenges.**

R: Mm.

**M: Now in all these aspirations that we have, you want to be lawyers, a doctor, what challenges would you see in the future that can hinder you from being a doctor or lawyer? Because even in school there are challenges that you face? Right?**

R: Mm.

**M: so in order to achieve your goals, what challenges do you see in future, what can stop you from becoming a lawyer?**

R: Having other people who are negative, and I am positive, I would want to be like them.

**M: Would that stop you from becoming a lawyer, that you are positive?**

R: No.

**M: It can’t right? What challenge would stop you from becoming a lawyer?**

R: Nothing.

**M: School fees, grades? No challenges? Not being accepted to a school because of grades, those challenges you don’t see? Or it will be smooth all the way?**

R: That will depend on your hard work.

**M: What else? And when it comes to dating and marriage, what challenges do you foresee?**

R: We will know when we get there.

**M: There are no challenges that you foresee, you know when you foresee challenges you are able to know how to deal with them.**

R: How you will tell your wife.

**M: How you will tell her of your status. What other challenges? You find someone, you like them, they like them and you see this is a potential wife.**

R: It will be a problem, telling them you are positive, they will think you are playing with them.

**M: You think they won’t take you serious?**

R: Yeah.

**M: What will make them not take you serious?**

R: How you will face them and tell them.

**M: That is the challenge?**

R: Yes.

**M: They will think you are joking?**

R: Mm.

**M:**

**M: Mm, it’s just normal.**

R: When you decide to tell each other your secrets, and then you cannot tell her about your secret, so you feel guilty that you cannot tell them about your status. So she can see how you behave and say there is something that you are not telling her, so when she keeps telling you that, it affects you.

**M: And if she keeps asking, do you decide to leave her or what do you decide to do?**

R: You just ignore that story and talk about something else.

**M: So the girls have that potential of knowing you are hiding.**

R: Mm.

R: My girlfriend has never known.

**M: She has never known that you are hiding something.**

R: Yeah.

**M: Mm-hmm what other challenge, how does it affect relationships? You are not there yet?**

R: No, I haven’t started that.

**M: Okay, do you have another question or another comment we can add? Something you feel that is important and you don’t want to leave without saying it? Is it there?**

R: No.

**M: Okay, and a question, do you have a question? Silence means it is there but you won’t tell me, or you are hungry and you want to leave?**

R: No.

**M: It means?**

R: There are no questions.

**M: Okay.**

End.
