# Supplementary material for: Psychosocial needs among older perinatally infected adolescents living with HIV and transitioning to adult care in Kenya
Source: PLoS One. 2020 Jul 29;15(7):e0233451. doi: 10.1371/journal.pone.0233451 (PMC7390380; doi:10.1371/journal.pone.0233451)
Supplement: S1 File — (ZIP) [file pone.0233451.s002.zip › uploaded final PLOS/Reviewed Transcripts/FGD4.docx]

**M: Okay, so when we start your discussion, rather, our discussion, I would want us to talk about how we were able to cope when we found out about our HIV status. How did you cope? I know it’s a deep question to start with**

R: It’s not that deep for me because I was told I was really young. I was in class five. I was just told to always take medicine, after all I grew already knowing so I don’t see it as a big issue.

**M: who was supporting you at that time?**

R: My mum

**M: your mum?**

R: yeah

**M: so how did she support you?**

R: let’s say, she reminded me to keep time when taking medicine, she told me why it is important to have a proper diet. She does this until now.

**M: this doesn’t have to be systematic, anyone can speak. How did you cope when you knew your HIV status?**

R: It was a bit hard for me at first because you are knowing that you have the virus in your body affected me a lot and I had a lot of peer pressure that eventually…

**M: peer pressure from?**

R: from the drug, taking the drug publicly. At that time, I was taking two times a day.

**M: twice a day?**

R: yeah so, I sometimes I used to take them to school with me and it was that period when I was still young.

**M: how old were you when you found out?**

R: I was in class six. So, I was like ah! sometimes I used to go and play a lot and forget. Some people say you are taking medicine and you’re not sick, why are you taking the drugs? So, I was like…

**M: explaining to people was difficult? (Silence) so, what did you tell people when the asked you such questions?**

R: at first, I used to stay quiet but then I become cleaver and malaria.

**M: Mm… so people stopped asking many questions?**

R: yeah

**M: and who encouraged you the most?**

R: my mum

**M: what method did she use to encourage you?**

R: that period when I was young, we used to come to the clinic together.

**M: (silence)eventually we all have to talk (laughs) if you stay quiet I’ll start pin pointing you.**

R: you know some people were not encouraged that much. Like for me, you know my mum also took these RVs so when it was time she used to just tell me its time. And you would tell that its also her time to take the medicine.

**M: so that shows you that you are in this together?**

R: yeah, we’re in this together.

**M: how do you think you would feel if your mother didn’t take them at that time?**

R: you know I got it when I was just a kid, class five, you just follow what others are doing. So, I was like if there taking these drugs then let me take them too.

**M: because you didn’t understand a lot of things?**

R: yeah, I didn’t understand.

**M: what about you?**

R: (silence) for me at first, I started getting sick that’s when I was taken to the hospital…

**M: I would like you ask you to speak with a little more volume because we are recording this. We want to pick you up properly.**

R: at first, I started with getting sick then I was taken to hospital. I don’t know how my mum was told but I know that we were told that we should be reporting to Kiambu. Then I was given the drugs to start taking. I thought I would take it for maybe one week then I’m done but I continued taking it.

**M: and at that time when you started taking the drugs, what did they tell you they are for?**

R: they didn’t tell me.

**M: when did they eventually tell you?**

R: when I was in class six?

**M: how did you cope when they told you at the time? (silence) it hit you hard?**

R: yeah

**M: how did you feel?**

R: you know I had already suspected at first.

**M: you had started suspecting?**

R: yeah

**M: what made you suspect?**

R: when I went to Kiambu and I was walking around the hospital and I would look at the posters.

**M: they gave you hints?**

R: yeah

**M: so, did you get discouraged when you realized you have HIV?**

R: yeah

**M: who encouraged you after finding out?**

R: my mum

**M: your mum?**

R: yeah

**M: how did she encourage you?**

R: she made sure I took the drugs on time

R: I would like to tell you this, I started when I was so young, I can’t even remember. I was so young when I got sick.

**M: about how old were you about that time?**

R: I was around like three years.

**M: so, you were just a baby?**

R: yeah, I was just a baby. That’s when I kept getting sick. Even when I continued growing I saw it s just any normal sickness. So just grew up taking my medicine and my mum never told me anything they just told me to take my medicine. If I ask what they are for the say your life depends on that. And I thought if my life depends on this then I continued taking it all through to 2015. That’s when I was called.

**M: that’s just a couple of years now, that’s like four years ago, three years ago?**

R: yeah then my uncle went out. In 2010 my dad passed, and we came with my uncle he went out and then I was told how it was. Personally, I wasn’t shocked because if that is how it was I didn’t get it when I was this big I have had it since I was a child.

**M: so, then you understood?**

R: yeah, I understood but then it made me have stressful thoughts.

**M: what kind of thoughts did you have?**

R: let’s say, they would rather have told me along time ago so that I would have known from a young age. Now I started getting stressed out, sometimes I even say to myself that I should stop taking them because its not like they are helping me and I stopped.

**M: at some point you stopped?**

R: yeah, I stopped then when I was coming to the clinic I don’t take the drugs all I did was lying to them that I still take the drugs.

**M: so, all this time you were still coming to the clinic?**

R: yeah but I wasn’t taking the drugs because I was stressed. But then it came to a time that this thing become serious. I was told my viral load went up to sixteen thousand. (9.34) and I had never heard such things before. So, as we wait for the rest to come they told us that this one has a viral load of ten thousand and I even wondered because I had sixteen and if you look at him, he looks really sick. That’s when I decided to start taking the drugs again.

**M: so, when you felt discouraged is there anyone at home who supported you or tried to encourage you to take them?**

R: at home they just knew I was taking the medicine, but they never knew why.

**M: so, who told you?**

R: my brother. He was the only one who knew I had it.

R: I started when I was small. I was always told to take the medicine. When I got to class seven, that when my mum called and told me at first, I didn’t believe it. I wondered where it came from.

**M: did you ask her?**

R: no, I didn’t ask her, I just kept quiet. So, I left there and went outside, I even ran away from home.

**M: for how long?**

R: about a week

**M: so, where did you go?**

R: I went to my aunt’s place. Stayed there a bit until I was told to go back and when I was back I was talked to and taken to a councilor where I was given encouragement then I accepted.

**M: so that time when your mum told you, what was running through your mind?**

R: I won’t live for long

**M: you felt like it was all over?**

R: yeah

**M: so, do you feel like it would have been better if you would have stayed without being told? Like you would be taking medicine without being told?**

R: it better being told because even if you think of stopping you are already told that it helps with something, so I can’t stop. If am not told, then I might think that this thing doesn’t help, and I can just stop.

**M:** **You can just ignore and decide not to take them?**

R: yeah without knowing its easy to decide to stop taking them because you can even tell yourself that I don’t even know what am taking. If you don’t know then you do not understand that you’re not sick and you are still taking the drug.

**M What about you?**

R: I started when I was still small

**M: please be a little louder.**

R: I started when I was still small. I was being given medicine and when I asked I was told that it for a cold.

**M: A cold?**

R: after some time, I asked again then I was told it for the chest. When I got to class six that’s when I came here, I was told to step outside a bit they talked to my parent a bit then I was called back in and that’s when I was told.

**M: so, when did you start using those drugs?**

R: when I was young

**M: when you were young?**

R: yeah

**M: so, class six is when you were called here and told?**

R: yeah

**M: so how did you feel when you were told?**

**M: (silence) what ran through your mind when you were told? Like he thought he would die so, what did you think?**

R: I started to think how other people would think if they found out and I feel bad.

**M: and have you told any of your friends about it?**

R: no

**M: and who helped you feel encouraged? Does it really affect you right now when you think about it?**

R: My brother

**M: your brother?**

R: yeah

**M: what did he tell you**

R: he just told me to take the drugs, that they will help me

**M: and now when you are just at home do those thoughts still come back?**

R: Mm

R: my story was that when I was young I didn’t understand what these drugs are for. I took them to about class four and that’s when I went to the clinic with my parent and that’s when he was told to leave, and I was left there then I was told. At some point it was really challenging after getting out of there thinking about what my friends would say, felt like my life was over, it was very discouraging, but my dad supported me. He gave me support when he told me stories. He is a councillor. So, he told me that other people are still living because they take these drugs and they still survive so that is what motivated me to take the drugs till now.

**M: so, you find out while you were in class four?**

R: yeah

**M: and what class are you now?**

R: form three

**M: so how did you cope emotionally after you found out?**

R: it was somehow hard but later after being encouraged, I felt much better

**M: and you continued taking the drugs?**

R: yeah

**M: what things do we feel that should have been addressed during or after disclosure at the counselling by clinics or councillors? (silence) what time would you want to be told at that time?**

R: I wish I was told earlier that am sick.

**M: you wish you were told earlier?**

R: for me how I found out was different, it wasn’t like in the hospital or anything like that. I found out just recently when I was in form two. I was stressing out my mum. She always knew that when Ian is going out, he’s going to drink and to use drugs. No one ever sat me down to tell me. How I found out is that one day I went out and came home late and my mum told me why are you disturbing me walking out there and you have HIV? Now at the time I didn’t take it seriously.

**M: so, you just got in the house and…**

R: that’s how I got to know. I didn’t even ask any questions the next day questions like how and why because I didn’t know if it was stressing her out. She thought that I wasn’t okay, but I was fine.

**M: so, you just found out when you were in form two?**

R: yeah, I don’t think that there is something that someone has to be given to be told.

**M: do you feel like if they gave you more information about adherence to drugs and that kind of stuff?**

R: yeah

**M: so, when you were told that night when you went back to your room did you think about it that much?**

R: I actually didn’t even think about it that much. I got there, and I smoked some weed, I got there and yes there was weed so, I didn’t even take it seriously. I just went on with my life as normal. I was thinking of where I was just from and I was on my phone texting was just busy. I didn’t take it seriously at all. When I started coming here I actually didn’t participate in the things that people were coming here to do. I would be called to participate but I never went all I did is to come to the clinic and sit next to my mum.

**M: Why though?**

R: I dint like to get involved with people. I didn’t want to be social. I always just sat next to my mum. No wonder I never even knew. So, when people were called in. those people were given tea with bread and I knew they were just going to eat and I used to tell my mum that am fine because we actually thought that maybe if you are hungry you go when they call. So I recently started joining them and listening to them talk is when I saw how serious it is.

**M: do you wish you had joined the group earlier?**

R: yeah, I wish I would have joined earlier

**M: how do you think joining earlier would have helped you?**

R: these issues of not taking y medicine wouldn’t have been there. Because there were sometimes I didn’t take them at all and I didn’t know how taking them would have helped me.

**M: so now you understand?**

R: yeah now I have started to understand

**M: and for the rest of us what are some of thee things you feel should have been addresses at the time you were told?**

R: to me actually I think everything had been said. However, it disturbed me so much, I was even doing research on it myself.

**M: so, you went on the internet…**

R: yeah because of a need to cure for it. So, I was very serious on it. There was even a day I went to a friend who invited me to a group. You know before I never used to attend these sorts of groups. So, the person assessing started asking questions and I was the only one who could answer the questions

**M: because you have researched extensively?**

R: I knew what viral is, I know what CD4s are, people who are positive…

**M: what are CD4s?**

R: CD4 is the amount of drug that takes your body to prevent it from to prevent…

**M: do we all know about CD4 or are we learning this just now?**

R: I have heard of it

**M: you’ve heard of it?**

R: yeah

R: so in that group no one knows whether you are positive or not but because you are just invited as just a listener, in your mind you know that your positive but in their minds they know you are negative. And at some point, the guy asking questions mentioned, to see this person is negative but he knows better than you. At the time the positive people were quiet because they didn’t know.

**M: so, nobody else knew someone else had…**

R: yeah because, I was being told if we go back there we should be talking to others. When I came here I was a bit angry and I asked a lot of questions until I know what I want to. Now there is nothing new because they told me everything.

**M: so, they told you everything?**

R: yeah

**M: is there any information you wish you were told at the time you came for counselling? (silence) have you been told about sexual reproductive health? (silence)**

R: as in sexual?

**M: these are things about sexual intercourse and contraceptives or any contraception information**

R: no

R: I can say that I have been told

**M: so, you’ve been given that information?**

R: yeah

**M: what have you been told about it?**

R: at first during the time I was researching and there was this time I asked, so, I am positive now in future if I grow up…

**M: if I grow up, why if?**

R: yeah if, because at the time I wasn’t sure I would make it because at that time before I started enquiring a lot there was a day I came very sick and so I was like am dying…

**M: okay before you continue, lets deal with this question of if. Has anyone else had that feeling, that if I grow up and not when I grow up?**

R: yeah

**M: you too?**

R: yeah me too

**M: what about you, you’ve never asked yourself about if I grow up and when I grow up? because there is a big difference. So, when you ask yourself if I grow up, what comes to your mind?**

R: according to me that if was because of the mentality I had in my head. That anyone who has HIV is a person who is nothing in the society, so I was like if I grow up. According to me, I did my research privately and I used to hate doing things like this in groups because people have their own opinions about HIV. I focused on what I got from the internet and I know that I stand on my own. So, if am asked something like in a group, I don’t tell them what is in my mind but what is in the internet.

**M: but really what is in your mind about it, do you think you’ll grow up?**

R: yeah, I think I will grow up.

**M: what about the rest of us, do you’ll reach a point that you’ll get a job, find a girl and get married?**

R: for me am already grown up

**M: you’re grown up?**

R: yeah

**M: so now you see that you have a full life ahead of you?**

R: yeah

**M: are you planning on getting married?**

R: yeah maybe getting married and having kids, maybe that’s ahead of me

**M: so that’s your next plan?**

R: yeah

**M: and for the rest of us?**

R: right now, for us people who are positive, we are advanced because now people can have children because of pep. That gave me encouragement of having kids.

**M: and having a family?**

R: yeah

**M: what about you, do you see yourself succeed as an adult have a wife and kids?**

R: yeah, I see I can because if it was passing on, that would have happened when I was young. Remembering what I went through when I was small, looking at where a from to where am headed, it seems closer to get to where am headed compared to where am from.

**M: and how do you feel, do you think you’ll be able to get kids and marry?**

R: I see myself doing that but am not sure

**M: so, its fifty, fifty for you?**

R: yeah

**M: what makes you feel like this, why are you not sure, is it that you feel like you’ll wake up one day and you’re not there?**

R: no, it’s not like that. My problem is how I’ll get a wife in the first place.

**M: is it because of people’s perceptions?**

R: yeah

**M:** so, you feel like getting a wife would be the tricky part, having to explain to her?

R: yeah

**M: but would you like to marry?**

R: yeah

**M: do you see yourself being a full-grown adult have kids and all that?**

R: yes

**M: what about you?**

R: yes, having a wife and kids (laughter). Look now we are talking about two trials for those drugs to, now be the time we are marrying, we would have worked right?

**M: yes**

R: and before that time comes they will have found the cure and we will marry wives here.

**M: how do you feel?**

R: yes, I will marry because when I was young we had guidance and counselling at school. So, they told us about drugs, disclosure and if one is positive and the other negative there are ways you can live without infecting the other.

**M: do you feel like you would need some training when you guys feel like you are ready to get married, get a wife, do you feel you would need some training for that?**

R: yeah that’s a must because its weird when especially when you are the man and you are the positive and the girl is not. And you are not like electricity that you will hold each other and produce a kid (laughter). There has to be a process that you don’t want to infect your child with the virus. So that training is needed.

**M: so, the training is needed?**

R: yeah

**M: and what do you think?**

R: (silence) you have to… we should get that training because of… (silence) you know training is not hard, but the main challenge is coping with the training, trying to explain why we should do things in a certain way

**M: you feel like that won’t be easy**

R: yeah, for me I always hope to just get lucky and get a wife that will understand me.

**M: for those in high school what do you hope to be in the future when you finish high school?**

R: I would like to pursue hospitality and management my other second option is being a doctor

**M: what about you what do you hope to be when you finish high school?**

R: at first, I wanted to be a pilot but now I want to be a doctor.

**M: what changed, what made you change from pilot to doctor? (Silence) there has to be a reason and both careers have a lot of studying you’ll still need an A.**

R: for me when I was small I wanted to be a pilot but its not even about education its about my mum. She came here to Kenyatta and the way people were treating her and not just my mother alone…

**M: other people too?**

R: yeah other people. It got into my head and thought let me be a doctor and make a change. Like there was one day I was going to the toilet while visiting my mum and there was a lady who was crying, my child, my child, my child. Then a nurse came and said get out of here, why you are crying? We have seen so many die just go to the morgue and you’ll get your child there. So, I was like is she serious, you are saying that to someone, the child died and is still lying on the floor and then they are still talking shit. Can’t you have some sympathy with the person? And so, I was like, it motivated me

**M: to want to be a doctor… so you want to be the avenue of change?**

R: yeah

**M: okay. What do you want to be when you are done with high school?**

R: I want to be a pilot

**M: what about you?**

R: a criminologist

**M: eh okay, why a criminologist?**

R: okay, I also had those big dreams of being a pilot (laughter)

**M: those are just dreams (laughter)**

R: yeah those are just dreams so, when I got to around class seven I saw reality (laughter) and I saw that’s somewhere I can’t get to.

**M: I can’t make it over here? (laughter)**

R: so, when I joined high school I took a look around and I was like corruption, I mean look at how much Kenya is exposed to crimes and then I like diplomacy a lot and am always ready to mediate on case of a fight. That’s why I want to be a criminologist

**M: eh, okay. What do you want to be?**

R: at first, I wanted to be an accountant but so now I want to do catering

**M: what changed from accounts to catering**

R: change? My maths was very low

**M: so, it’s our results that make us change our career mostly?**

R: yeah, but even the luck of money because I know even if you have failed and you want to be that pilot or whatever

**M: you will just be what you want**

R: you will just be another problem may be, let’s say you wanted to be a pilot but then when you move forward you are interested with something totally different because you might be performing badly in the subjects you need for the career you wanted but perform better in other subjects, then you eventually decide to pursue what you do best.

**M: what do you want to be?**

R: electrical engineer

**M: what would be the challenge of being an electrical engineer?**

R: I want to be one but am still afraid of electricity (laughter)

**M: that’s one challenge, fearing electricity**

R: another challenge is the luck of jobs.

**M: luck of jobs for?**

R: for paying that school fees now

**M: so, some of us have to hustle so that we are able to pay our own fee?**

R: yeah

**M: what other challenge can we have?**

R: if I make it to be a criminologist my challenge is that I don’t want to die. That job has so many consequences. Maybe people calling you a snitch and am still helping these Africans

R: and for me I wouldn’t like them to know my status.one can see my picture and say we can’t even eat inside here and they all leave. When you try to explain they don’t want.

**M: now that you are positive, do you think that if you get a patient, they would let you treat them if you told them you are positive?**

R: very well because they know there is someone who is looking out for him somewhere because if you sit with them they can convince you when they tell you that even they are positive you take this. Because they would understand because he has the experience, or she has the experience of what you are going through than a person whose there from nowhere he doesn’t know anything, he doesn’t know how you feel or how you cope with your life and the patient will feel at peace because this is normal.

**M: does everyone feel the same way**

R: yeah. We are always told that, so you know a negative doctor can’t come here and teach you and tells you things that are nothing, you can’t listen to them because they know its me who has it and not them so they don’t know how I feel so you cant listen to them

**M: so, do you think the time you weren’t taking your medicine, is it because the doctor was negative and doesn’t understand you or what was it?**

R: I didn’t know whether he was positive or negative. So, I asked why they didn’t tell long before but maybe if I was told when I was smaller I would go tell your friends that I was in the hospital and I was told I have HIV innocently and being told as an adult then you can know how to handle that situation

**M: are there sometimes we feel like we don’t want to take ARVs?**

R: there are sometimes you find that they are many

**M: like how many?**

R: let’s say five

**M: five tablets?**

R: yeah and you know that in the evening there is one more needed so that’s six.

**M: so, you take five in the morning and six in the evening?**

R: and sometimes you might be added some more let’s say like two more for TB

**M: wah, those are eight?**

R: yeah, and when you look in the kitchen there is nothing, so you can just decide to take them in the evening when a hill had been made (laughter)

**M: a hill of ugali?**

R: yeah, so that’s when I say no

**M: so, you say no so that you can wait for food to get prepared first?**

R: yeah

**M: are there other times we don’t feel like taking the drugs?**

R: for me there was a time, my aunt, no one knew it was just me, my father and my mum, now my mum passed on in her bed so she had told her sisters and I didn’t even know that they knew and my aunt would call and ask me if I have gone for the medicine and I would always ask her why should I go for medicine and am not sick. So, one of my aunts stays in London and she asked me why I don’t take my medicine, then she asked how many I take in a day and I told her I normally take two in the morning and two in the evening and she would ask why you should only take once, you know there they have advanced medication, now she discourages you.

**M: so, after that you were discouraged and stopped after some time?**

R: for some time but then I came here, and I was told there was a doc here, I don’t know doctor Jumma, who told me you can do better that this, so you just take your drugs then well see what can happen. Me I was devoted and started taking the drugs and now am using a one dose drug

**M: just once a day?**

R: once a day just one pill for the whole day.

R: maybe I decided to stop, just recently when I was done with my form four. You know I had even decided to move away from home. I got my self a small place. Insurance gave me some money. So I felt like I made it, so you know when you stay alone, there are some things that just come up, friends may come and tell you lets go there and by the time you go there and walking around with them you forget that time has passed, so it passed like that for two three days and you say ah, nothing is even happened I would rather just stop so when I pass by hospital is when am told this and that and my viral load had gone up. And you know me sometimes I don’t take things seriously. My mum called me and told me that I should just go back home, and I told them that there is something am still thinking about but then I came back, and I was talked to about college and that’s when I decided to quit my job and she said it’s okay you just come and that’s when I started to take the drugs again

**M: is there anyone else who has stopped taking the drugs for some time and you see that you just survive? Since you started you never stopped even one day?**

R: maybe forgetting. You know sometimes you can go to your friends then you get late coming back home now you can’t take them. And let’s say at the game and its fun even if you start talking about the drugs, maybe coming to take them at night when I come to eat food,

**M: have you ever not taken the drugs for some time?**

R: yeah

**M: for how long?**

R: like one week

**M: about one week?**

R: yeah, the week I ran from home

**M: the time you ran away from home, is there anything that affected you at that time?**

R: no

**M: so, if you were asked you would have stayed longer, maybe more days or a few more weeks?**

R: yeah

**M: so, what made you start taking them again?**

R: I was called

**M: you were called by your mum**

R: yeah, then she told me to go back home

**M: do you think these drugs have side effects to us?**

R: yes, they have side effects

**M: like which ones?**

R: like sceptrin, its toxic in our body

**M: how?**

R: it dries you, it dehydrates you

**M: oh, it dehydrates your body?**

R: yeah so you need to take a lot of water and if you don’t take water in plenty then you’ll be dehydrated. Looking at me I wasn’t taking water well and you see the effects

**M: the black spots?**

R: yes, these black spots, that’s a side effect and then the one drug that I used to take you feel like when you take it its like you are drunk (laughter)

**M: you don’t feel a hundred**

R: you feel like even when you stand up you just go so that thee reason they advise you to take it at night. But at some point, it goes away

**M: what other side effect do we face? (silence) there is that that you feel drunk and dehydration…**

R: there is a certain gas. How can I say this if you don’t drink water properly there is a gas that is produced that burns here…?

**M: like heartburn**

R: yeah like a heartburn but its not a heartburn and you can steel feel the taste still when it burns. It feels like you are removing the smell of the medicine from your mouth. Also, sometimes if you don’t take enough water while taking these drugs they come and crystalize in your liver and that may make some complications you can get liver cancer.

**M: eh, okay. So how do these side effects make us feel? (silence) How does it make you feel**

R: (silence) to me I would say sometimes I wish I didn’t have this virus so that I don’t have the side effects of these virus.

**M: so sometimes you may wish that…**

R: to me, what I can say is that, the side effects are there but there is a way you can, not control them but you can reduce them.

**M: how?**

R: you can, like taking water. Like in a day you make sure you take at least a litre and a half.

**M: of water each day?**

R: of water to detox your body. Diet, your diet has to be…

**M: you diet also has to be, not just eating chips and sausages**

R: yeah, not chips and those things. Your diet has to be good. You need to take care of yourself because everything has a way of getting help.

**M: when you explain those side effects, do they make you want to stop taking these drugs?**

R: completely. You just feel like you want to stop

**M: makes you feel like stopping?**

R: yeah, especially the first one that makes you feel drunk

**M: that won’t be something that people would say that makes me feel high? (laughter)**

R: there was a time I was in school, I was in boarding school and I messed up. I thought it was Panadol and I took it in the morning then I got into class. And the teacher was like teaching and I was like, so the teacher as asking what is wrong with you. Even I was taken to the office because of that issue.

**M: it became an issue?**

R: it become an issue explaining was hard

**M: and you can’t start telling them**

R: when the teacher asked I told them, teacher am not feeling well, let me go, please, write me a leave out please let me go. They even checked my alcohol level

**M: they even checked your alcohol level?**

R: yeah, you know out dorm had notorious boys, so they thought maybe…

**M: maybe he had tasted something.**

R: he has tasted something, so they checked me, and I said no, just take me home and I will know what I’ll do. So, I went back home for a day and relaxed then that’s when I said am not taking the drug if it does this to me.

**M: so, the way you said the side effects of make you want to stop the drug, who else has experienced that?**

R: I take mine at night and when I do I feel dizzy and like throwing up and I can’t even eat because I lose my appetite.

**M: and change of routine, can that be a reason that makes us not to want to take these drugs? Maybe you got into boarding school and that kind of stuff. Have we all been in boarding school?**

R: yeah

**M: you haven’t been to boarding school? But the rest have been to boarding school.**

**Are you boarding?**

R: yes

**M: so, when you were boarding did you ever feel like let me leave these drugs for some time so that people don’t know?**

R: yes, when you were admitted.

**M: when you were admitted so how were you able to deal with it?**

R: so, before I decided to carry my drugs, they used to just stay in the dorms now when asked the head boy permission he would let me but the teacher on duty didn’t care(laughter) and the teacher would beat me mine and you would just let the drugs stay in the dorms and when I come back is when I’ll take them. Or in the morning when you wake up at three thirty

**M: you woke up at three thirty in the morning? Going where?**

R: to class

**M: its like you got into an army barracks (laughter)**

R: okay, we woke up at three thirty, then at three forty is when we go to class so when the patron gets in again you even forget the drugs, you even forget to lock your box now if you forget to lock your box what about the drugs? Then you start going back when you feel a cold is coming.

**M: then it becomes a bit more serious, and, let me come to you. When you are in boarding to you feel like you don’t want to take your drugs?**

R: no, for me it hasn’t been challenging because none of my friends knew I was taking and drugs. Where I was being advised there were sachets that I was given so when I left the dorms I put them in the sachets then put in in the locker. So, when it gets time to take them I just get out and take them and no one knew.

**M: so, nobody knew what it was?**

R: yeah

**M: is there anyone who ever asked while you were in school what this is you are taking every day?**

R: yeah

**M: what did you tell them?**

R: I didn’t even tell him I had just been given the medicine like this, you see how the sceptrin packets are written Abonza, and so this guy started calling me Abonza. So, I was scared and it held and held so when I checked the pack well is when I saw it was written Abonza. So, people started asking me so why are you called Abonza. So even till today people call me that.

**M: so, people started calling you Abonza. That’s a nick name you can’t explain well.**

R: even me there was a time I was caught when I was in form two. That time they were doing a search in the dorms and then that time I hadn’t taken out my drugs because we were just taken out by the dorm master. So, I took the drugs and poured them in my sweater. So that evening getting in the dorm I find the deputy he told me open your bag, your trunk. When I opened he saw the drugs and asked what are these? I told him they are medicine teacher does anyone know? And then everyone came to look at them because they were many I was even taken to the office

**M: so, you looked like a peddler**

R: yeah, before I was taken to the office I was asked if I want to she school nurse? And I said when I was admitted, so I started making up a story, I went the last morning its just you guys that don’t know. So, and this was something bothering because when I went back the dorms everyone was like he can die here

**M: because they were so many?**

R: people said that that one has a problem he’ll die on us here. It even got to a point that even the deputy came to class the deputy could come after class to ask me if I took my drugs? And that was a bother to me because I hate that.

**M: so, did he know what the drugs were for?**

R: no because I had left before it came to that.

**M: and between day and boarding which one would you say it will be easier to take your drugs?**

R: its day

**M: day is easier**

R: because boarding someone can ask you what these drugs are for and you can also be late

**M: what about the rest of us, when you look at day and boarding, which is a bit easier?**

R: day was good because I also studied in day

**M: you studied in day? And that was easier for you?**

R: yeah because its okay so you can just change the timing, in the evening and in the morning when you leave.

**M: oh, so you are allowed to change time, or you have to consult the doctor first?**

R: you have to consult first.

R: you have to?

**M: did you change your time without consulting?**

R: yeah, I did, even now

**M: even now you’ve changed your time yourself?**

R: but if you change and then you are strict then its fine. Because I have asked the doctor because I ask him, and he told me if you ae serious you want to change the time then take it to be serious.

**M: and (silence) do you feel supported about your HIV status at home? Do you feel supported**

R: (silence) you know sometimes you are supported so much that you feel as if you ae being tricked into something.

**M: because you are being treated very well**

R: yeah even better than others

**M: and is that a good thing or a bad thing?**

R: its not something bad but sometimes

**M: your treated so well that its noticeable or**

R: yeah and sometimes, you see the first day’s mum comes with fruits and says no one to touch it.

**M: so, it’s not like you want special treatment that you are bought fruits or milk and that its for you alone? Have the rest of us experienced that mum has brought something and says that this is yours alone?**

R: yeah, even now, and I hate it

**M: even now?**

R: yeah, and you a be hated

**M: you can be hated?**

R: yeah, let’s say that some oranges and maybe bananas are said that they are yours and the others have not been given and they always treat you unfairly and when you are not around they even give you little food.

R: when you are left in the house and you leave for a while when you come back you will find that…

**M: they are all finished up?**

R: yeah. For me even now, when mum was around and now she passed when anything happens they ask you and when I ask them why they are asking me, you are the one like your mother’s shadow everything they can’t even say it’s their mum they say your mum.

**M: and they ae your brothers**

R: everything is done for you if you ask for things you are given. It doesn’t create a good image they act as if you are special

**M: the see that you feel so much more special than them**

R: yeah, even now if my dad asks me how are you feeling, and I ask him back how am I feeling? (laughter)

**M: and do your brothers know about it?**

R: no

**M: and do you feel like if they knew they would understand more?**

R: eh! No

**M: no? why, you have disagreed so fast. You don’t want them to know at all.**

R: all my support basically comes from my dad

**M: do you have both brothers and sisters?**

R: yeah

**M: but you wouldn’t want them to find out**

R: yes

**M: why not?**

R: my brother can turn against me. And you can also be stigmatized. Do you know how bad stigma from family is always so bad?

**M: yeah**

R: even if you are just sitting on the chair everyone sits far away from you. Have you used this cup right now (laughter)?

**M: I can’t drink from that**

R: your cup, your plate your spoon they can even write your name on them (laughter)

**M: they even write your name on it, who has seen anything like that? There is something you were saying before that**

R: I was saying that, if all your brothers all know if you do something wrong to him in the house the guy can beat you up and tell you to get out of here, you got sick in the first place (laughter) I don’t even know you, things like that. Only one person understands.

**M: so, its better your parents know or the oldest on to know**

R: yeah

**M: but the rest its bad. What were you saying**

R: when it gets to the time your names are written over there

**M: on your cup and plate, has it ever got to that point with you?**

R: it’s happened that I was written to like that. There was a time I got sick. You see a cold

**M: yeah**

R: those that comes out from here that makes you look dented. The had to do that because it took time to heal

**M: did you feel a bit stigmatized when people started to do this to you?**

R: I just came out and told my mum never to do such a thing because.

**M: labelling your things aside from the others.**

R: yeah labelling things

**M: was she labelling things?**

R: no, she wasn’t labelling things, but I just told them because they saw as if I drunk that tea and then place it there, within ten minutes the virus is already dead there is no other way its dead as long as its out of the body, within ten minutes its dead so you cant say that it will affect me

**M: so, has anyone ever told a close friend and then they went against you? (silence) or any close friend about your status, girlfriend, best friend, cousin**

R: I told a few friends of mine they know.

**M: they know**

R: yeah

**M: did they change their perception about you?**

R: no, they treat you just like a normal person

**M: they haven’t side lined you and said they don’t want you to be friends now?**

R: no

**M: is there anyone who has told their girlfriend?**

R: I have never told any girl, what I did is my auntie told her children then her child was my girlfriend’s friend, so she went and told her. And that girl left. From that Now I see that I will wait for a time when I will be ready to tell and not someone else say it on my behalf.

**M: what were you saying**

R: I have before but what I came to notice is that you never tell someone who lives in the same area as you (laughter)

**M: they should be far**

R: yeah look for someone from far, so that in case you tell them she can’t know where you stay but if you tell someone who lives close to you, if they see you with a girl they just got tell het this guy has this and that. If you go tell one person that they say walk yourself to heaven.

**M: have you ever told a girl? Or any other person like your friend**

R: (silence)

**M: and you think, like the way you told that girl, how did she receive it?**

R: I told her there is something I have to tell you and she said its okay. So, I asked her why do you love people, the way they are or the looks and she said I like the way they and their looks, so you don’t like someone with your heart, I said I love people with all my heart but there is something in me and that is what I want to tell you. I am HIV positive, then she asked what did you say, are you dreaming or are you just saying? I told her that it’s the truth then she said let me get out a bit am coming back, and she had even left her bag in the house and that’s how she left.

**M: there was her bag there**

R: she left the bag empty right there

**M: the bag was empty?**

R: nothing, just some small things

**M: did she ever come back for it**

R: no

**M: so, you’ve never talked after that?**

R: not even a call, if I try calling her, I think she’s black listed me

**M: since you haven’t told people, how do you feel they will receive it?**

R: it depends on who you are telling because this girl of mine that I told, I was the one scared when I told her that I don’t want it, but she took it well and we are still friends.

**M: so, she’s never changed her perception about you**

R: no, she’s never

**M: so, you are the one who just saw that… do you think she received it well?**

R: yeah, she received it not in a bad way, but she would have said that maybe in a relationship we shouldn’t have gotten that open.

**M: and who would you like to tell about your status and get support from them? It can be your friend, a family member…**

R: friends are good, that’s how I see

**M: friends are good, why?**

R: that’s just how I see it

**M: that’s how you view it?**

R: yeah. Family members.

**M: you wish you could tell them?**

R: yeah because friends disagree, friends disagree you can even stop being friends so if you tell them.

**M: what about the rest who would you like to tell? (Silence) no one, what about you (silence) , who would you like to tell (silence) no one what about you?**

R: family

**M: so, when you say family do we mean direct family or even extended family? Like uncles or do we just mean like brothers?**

R: brothers

**M: so direct family. You guys have said no**

R: its better a friend because if they are going to talk about you, they do it out there, there was a time they are talking about you in the house and you are there.

**M: so sometimes they talk about you and your there?**

R: yes, you even wonder, I even stood up and told them, you talk, talk and talk until you get tired because am sick and tired with you, me I will work, I will study and finish then I will take care of myself so don’t worry.

**M: so that was your uncles or?**

R: my aunts, my uncles don talk but my aunt you now

**M: do you feel supported by the people who know, do the people who know support you?**

R: (silence)

**M: do you all feel that way? Who supports you mostly?**

R: my father

**M: your father? What kind of support do they give you?**

R: (silence)

**M: okay while he thinks about that, who else is supported in their family? Are you supported?**

R: yes, buy by uncle

**M: your uncle?**

R: yeah

**M: how does he support you**

R: he supports me with food and takin my medicine, that kind of stuff

**M: what about emotionally?**

R: (silence)

**M: because you know as men we don’t like showing our emotions. It is very rare you find a guy sitting down and crying telling another guy, but in the real sense all of us guys have emotions it’s just that we don’t show it. We can even deny it if we are asked. So who gives us emotional support?**

R: (silence)

**M: silence means no**

R: only God (laughter)

**M: who else, its only god (silence) so all of us it’s only God? What about you**

R: only God

**M: do we wish we had people who support us emotionally?**

R: (silence)

**M: someone who would come along, and you can tell them the real truth and your sure they are not getting it from somewhere else> that is going on would you like to have a person like that?**

R: I have a cousin

**M: you have a cousin?**

R: who supported me, my aunt even if they talk about me, the do support me financially if am sick or even if am not sick they tell me do you have fruits. There is a person who I exposed myself to and I told them everything and me I prefer a person who I will tell, and they wont pressure you. You know there is a person if you tell they even look if they are shocked and they start asking to have you gone to the clinic, have you done this, have you done that. So, I don’t like that, so I prefer to stay on my own. I get support financially but emotional support just from one of my cousins.

**M: do you wish the hospital would create you an avenue for someone you can be have real talk with even if its once a month to tell them everything your going through and you are assured. What if the hospital gave you something like that?**

R: I prefer the hospital, you know in hospital you can come you talk and you go personally and then you go. Even if you tell those stories when you are hurt until you are done and gone, even the place you were talking at someone else will come and do the same then hell forget what you told him.

**M: so, you already get that platform here in the hospital?**

R: sometimes

**M: so am saying if you get someone dedicated, someone you will be coming to even after seeing the doctor. Just getting someone, you can download all this information to. Can that help?**

R: yes

**M: how do you feel**

R: for me am good just as I am. The little I get from the councillor and the doctor then am good

**M: what do you feel?**

R: it would be good because even sometimes talking to someone gives you some relief that you didn’t have, so sometimes its good/

**M: so, having said that, is there anything that maybe you had confined when we started the discussion and you want to say it now? (silence) anything, it doesn’t have to be something big. So, have we ever experienced stigma?**

R: yes, but not with the drugs

**M: because of?**

R: am short (laughs) you know being short and you have to associate with big people then you are like, we play games, so I decided to stay indoors, because indoors is easy to apply and you can play with others because it does not have many people. I play ball by the way and I am a crazy defender since I was in primary but joining anestar you know, finding other people and they are tall and they say are you the one going to hold the defence for us, the ball will pass over your head. Somebody else accepts you so that he can eat your money by setting up long processes

**M: then you still don’t play**

R: yeah you still don’t play, you go for basketball, just for fun not even to play seriously. If I try shoot the ball you hear people saying that won’t even get there. Another thing is being underestimated like you can’t do anything big

**M: anything bigger than you. You are getting limits from how tall you are. We all understand what stigma is? (Silence) for the person who doesn’t know, stigma is a wrongful perception that people will think about you and will make them treat you negative way. Knowing that have you experienced stigma? (Silence). So when you experienced stigma in sports how did you deal with it?**

R: I become enthusiastic and had psych

**M: you get jovial and become the guy who is psyched up**

R: yeah and I have it even now.

**M: have you ever experienced stigma? You have been so quiet tell us something. (silence) Are you feeling shy? Have you experienced stigma? Please talk. if you shake your head it won’t hear you. (laughs) what else would you like to talk about as we finish, something you feel we haven’t talked about, about anything and everything? (silence) or a comment you would like to make? (silence) let me ask you a question first before we finish. Have you ever disclosed to someone about your status you expected they would have received the information than how they received it?**

R: my family

**M: are you the one who told them personally or someone else did it? Have you ever told anyone personally?**

R: no

**M: do you feel like you are ever going to tell anyone personally?**

R: that depends with the kind of person

**M: that’s just opened up another avenue, do we feel like we’ll ever tell someone else about our status personally?**

R: it’s really hard

**M: why is it hard**

R: personally, it’s really hard

**M: do you think you will ever tell anyone personally about your status?**

R: okay its not easy but I have disclosed to one person, a close friend personally and he took it positively after that he’s been supportive throughout.

**M: so knowing that can you go to disclose to someone else?**

R: not really.

**M: so what made you trust this person to that extent?**

R: he was a close friend and we have been together since we were young so there is that trust that just came naturally.

**M: do you feel like you will ever disclose to anyone personally?**

R: at some point I will get there

**M: at what point?**

R: when I want to marry

**M: when you want to marry**

R: yeah, because its not like you are going to tell your parents to tell them of your behalf, you will just have to man up

**M: you man up, so its not easy?**

R: yes

**M: do you think you’ll ever tell anyone personally?**

R: when I marry

**M: just your wife, if its not your wife you won’t tell anyone else?**

R: and the kids

**M: oh, and the kids maybe. And will you ever tell anybody personally about your status?**

R: am not disclosing but I found out about my status by myself

**M: how?**

R: a long time ago when Kenyatta was using the file system, now my first document has the information about my status. So, every time we came here its my dad who was given that file and he neve left it anywhere, he used to hide it between his newspapers. So, one day he went to the washrooms and left it with me, so I opened it up and there was my name with HIV. So, you know you don’t want to tell him you know. You know that feeling? Now there was this time he was shouting at me and said, if my dad finds out. He even gets shocked and he knows.

**M: how did you feel, oh, you weren’t here when we did the introduction. So how did you feel that time when you read it and found out?**

R: at first, I was afraid and then it become like food for thought. Our days the we had those phones with many buttons, now I was in a hurry to google, I didn’t even have time to convert into bundles. I searched to see if it was HIV. At this point I was certain it was HIV, but I didn’t want to say. Because I will look like am loose and that am digging in into something I don’t want to know

**M: so, what went through your mind the moment you saw you have it?**

R: where did it come from?

**M: where it came from**

R: where did it come from, now I didn’t know, and I didn’t want to ask my dad?

**M: have you ever asked him?**

R: now that day he was shouting at me and when we all eaten we were mad at each other because he was not telling me.

**M: he wasn’t telling you. And how did you cope up emotionally after you found out?**

R: I was told there are many people and there is this fellow who writes in the standard who also had HIV and he gives out updates so knowing am not alone then to hear some people, I mean I know we are many

**M: who is that in the standard who has …do we think we can be that bold. Like that guy from standard to publicize yourself like that one day you can be bold enough lest say like that guy from standard to say I am HIV positive publish it in the newspaper**

R: as long as you have made it. (laughter)

**M: if you have made it, if you have something (laughter)**

R: and if you are a musician. And there is this actor in Hollywood who came out and now he is rich, I have forgotten his name. he published and even went to the newspaper, now nobody talks because he’s rich.

**M: so, you feel if you are rich people will change their perception**

R: yeah

**M: if you tell anyone they won’t reject you, if you have no money you have no say**

R: status will still be rich (laugh)

**M: so as we finish is there anything else you would like to add, anything you want to say (silence), okay, thank you so much for coming, for sharing your thoughts and opinions with us I really appreciate it.**
