# Supplementary material for: Psychosocial needs among older perinatally infected adolescents living with HIV and transitioning to adult care in Kenya
Source: PLoS One. 2020 Jul 29;15(7):e0233451. doi: 10.1371/journal.pone.0233451 (PMC7390380; doi:10.1371/journal.pone.0233451)
Supplement: S1 File — (ZIP) [file pone.0233451.s002.zip › uploaded final PLOS/Reviewed Transcripts/FGD7.docx]

**FGD7**

**M: So we can start with the introduction because we all don’t know each, at least we should know each other then we continue. By the way which language are we comfortable with? Don’t tell me French or Spanish (laughter).**

R: Swahili.

**M: Swahili is fine, okay, so we are going to start with introduction, and maybe you will just tell me anything about yourself, one name and anything about yourself that you would like us to know. Now because you have volunteered you can start (laughter) he has volunteered right?**

R: Hi.

**M: Hi, we have answered you, don’t stand, I have told you that it’s not a class and I am not a teacher.**

R: My name is XX what else?

**M: Anything that you want to tell us about you.**

R: I have nothing else.

**M: Then a hobby, tell us your hobby.**

R: Dancing.

**M: What type of music do you like?**

R: Gospel

**M: Gospel, but dancing you know has beats, there are those that are slow, three is reggae, there is…**

R: No not reggae, reggae is for reggae people.

**M: What kind do you like Kigosho?**

R: Not Kigosho, just the ones like for XXX

**M: Oh local, thank you for being the first one to talk.**

R: Yes.

**M: Okay.**

R: I am xxx , I like drawing.

**M: Drawing, can you draw me?**

R: Not exactly.

**M: You can try?**

R: Yes.

**M: Okay.**

R: I am xxxx, and I am cool and god fearing.

**M: Eh.**

R: I like drawing.

**M: What do you like to draw?**

R: People.

R: Can you draw me?

**M: People okay cook.**

R: Hi my name is xxxx, in my free time I like listening to music and swimming .

**M: Mm.**

R: Hi, I am xxxx, I like art and designing things although I don’t have a lot of creativity.

**M: You like design?**

R: Yes.

**M: Isn’t designing creativity or?**

R: But not…

**M: You feel that you are not there yet, but with time you will.**

R: Hi, my name is xxxxxx, I like music, swimming, dancing.

**M: Like him?**

R: Hip hop.

**M: Oh.**

R: Hi, I am xxxxx, I like watching TV. (Laughter)

**M: Anything on TV?**

R: Cartoon.

**M: Cartoon.**

R: Soaps.

**M: You all like telemundo?**

R: Hi I my name is xxxxxx and I like swimming.

**M: Okay, fine, and you all remember my name?**

R: Yes.

**M: I like many things, swimming, one thing, but I have not swam for 5 years and then I went to swim and I thought I got pneumonia. (Laughter), I had not swum for a long time, maybe now I don’t like it, I used to like it. So I would like us to start our discussion, discuss our experiences in this clinic, how have they been? xxxxx how has your experience been?**

R: Coming here?

**M: Yes in this facility.**

R: Good.

**M: Good, someone else to help her to add to that, when she says good do you agree with her?**

R: It has been good.

**M: How has it been?**

R: It’s good because we feel like we are one family.

**M: So at least feeling like family is good thing.**

R: Mm.

**M: What else is good? You know if 5 people say good, it means different things, like if I say I like tea, maybe you like lemon tea, maybe you like with milk, or you like with a lot of milk. If we say that tea is good, all of us mean different things. So I want to understand, we say that our experience has been good here, what do we mean?**

R: Its good because the docs try to motivate us.

**M: Mm, someone else? What do you want to say? xxxx?**

R: I would like to support xxx, its good because they motivate us, and you find that when they find your viral load is not down, they tell you that you have not been taking your drugs but you should continue so that your viral load is low.

**M: Mm.**

R: It has been good because they motivate and they are supportive and they also talk to parents like now when they called us for support group, they called our parents so that we could come and then they are also friendly.

**M: Anything else that you want to add that you forgot to add?**

R: It is good because doctors motivate us and there are many topics that they teach us that tell us things that we don’t know.

**M: Like which topics?**

R: How you can take the drugs, the challenges that you will face.

**M: Hmmm, do you want to add? How long have you been coming to this clinic? I am sure we didn’t come the same day. When did you start to come?**

R: When I was young, I can’t remember, I used to be brought here.

**M: When you were breastfeeding?**

R: Yes.

**M: So you can’t even remember.**

R: Mm.

**M: Okay.**

R: Me too.

**M: You too.**

R: 10 years.

**M: When was this?**

R: 2007

R: When I was 12 years.

R: I can’t remember the year but I was 8 to 7 years.

**M: Hmmm.**

R: I was not in this institution, I came last year, I used to go to Machakos because of school, but I transferred and came here, but I started there when I was ten years.

**M: You started when you were ten.**

R: Mm.

R: I started when I was 9 , in 2009.

**M: Okay, so I want you to tell me about experiences that you had the first time you learnt about your status, how did you feel and how did you know?**

R: For me I didn’t react because I was young, and I was told that when I was born, I was born with it, so I didn’t react.

**M: You didn’t know.**

R: Mm.

**M: At some point you knew you were infected, but you grow without knowing right? But there is that day that you are told and you get to know your status, how was your experience that day? Naomi don’t be stressed, how was it?**

R: Mm I was disclosed to when i was 10 years, but since I didn’t know what it was I did not react.

**M: Did you have questions?**

R: No I was just fine.

**M: Who disclosed to you?**

R: Mom.

R: For me I didn’t know till a day I got malaria, and I was taken to the hospital I was not treated, I was told to come the next day, I waited some years later is when I was told I have it, I was told a story about animals that died, and that helped me.

**M: How did it help you?**

R: It was about hare and his family, they were sick and they all died except one that trusted themselves, and they were left with hope that they would grow up to be big.

**M: So how old were you?**

R: 11.

**M: So at 11 when you read such a story, what kind of impact does it have on your life? How do you feel?**

R: The story shows how you should congratulate yourself so that you can see yourself as a person and move on.

**M: To get encouraged.**

R: Yeah.

**M: How did you learn your status, is there someone else that wants to share?**

R: I was tested, I didn’t know, I had some problems and then I was tested and I found out.

**M: What problems?**

R: Health.

**M: Who brought you?**

R: My mother.

**M: And who told you?**

R: The doctor.

**M: How did you feel? Are there some questions that you have but you have never asked such questions but you ask yourself in your head? Let us not fear speaking. It’s just a part of life, it’s not only status, we all have questions about life, I want you tell me when you are alone, those questions that you ask yourself.**

R: I ask myself where it came from.

R: I ask myself why me?

**M: Mm.**

R: Sometimes I am told to just accept myself because I am already infected, but then I wonder why take tablets, is there something like needles that you can be given that last for 5 years.

**M: Instead of taking tablets.**

R: Yeah, by the way I had heard that story of being injected and then you come back the next year after that I didn’t hear it again.

**M: Where did you hear this?**

R: On TV.

**M: So you feel that is a better option.**

R: Yes.

**M: So taking pills, how is it?**

R: That life is hard, sometimes we miss taking them because you get tired of carrying the bottles making noise at you, it gets hard.

**M: Mm.**

R: Sometimes you are with your friends, so you don’t have that courage, your friends are keen, they want to know what you are doing, so it’s better we get injected because no one will notice.

R: Or they put an implant in us.

**M: So the biggest burden is the tablets.**

R: The problem is not taking them, the problem is how to take it.

R: It would have been better if it was liquid.

**M: When you take the pills how do you feel?**

R: You don’t feel anything but say you are in a party with friends and family, you find it hard to take them because you will be stigmatized.

R: So there is this issue of stigma that is coming in.

**M: Mm, is there anyone who has been stigmatized at any point because of their status?**

R: No.

**M: So if you are quiet I know its no. okay so have you been through the disclosure counseling in the clinic or our experiences have been different? Have you been through any counseling about disclosing status? You have?**

R: Mm.

**M: How many have? And there are those who haven’t.**

R: Mm.

**M: Do you remember anything that you were told in this disclosure counseling?**

R: Yes, if we take the drugs and feel that we are healthy we should not stop taking the drugs, because that would not work.

**M: Something else that you remember?**

R: Also you should accept yourself because this will be like this your whole life.

**M: Mm.**

R: So we should accept the situation.

**M: Anything else?**

R: If you take those drugs, you should not take them for granted, your whole life is there, everything is there, because if not for those drugs, you cant live.

**M: So it’s like having a good relationship with the drugs. So do you have an issue of telling other people your status?**

R: If you tell people your status they will avoid you.

**M: They will?**

R: It’s not easy, you just have to hide it.

**M: Hmmm.**

R: First it’s not easy.

R: You should not disclose to everyone, you should study how someone is at first and then after some time, you can tell them, like for me there are those I can tell and then there are those that I can’t tell. Like if I have a boyfriend, then if I tell this one, you know boys meet and talk about you, you will get stigma, I cant.

**M: Mm, so telling people is hard.**

R: You have to know how this person is.

R: It’s not easy, like with friends, you can’t live on your own, that you don’t want help from your friends, you will need help. But you can’t just tell them your status directly, you have to wait to tell them, you know many of the things you have told them, they would never say. So you tell them after you have studied them and you know they don’t talk about you. Because if you tell someone and then they go and discuss you, you will have low esteem and that is not good for someone.

**M: Someone else what do you feel about disclosing? Derrick, you want to say something?**

R: No.

**M: There is no one who has disclosed to someone?**

R: As a person there are things you have to disclose so that you are not depressed, as in the person you are going to disclose to, you know also you can make friends with negative people so that you can exchange experiences, so that you can see that your experience is different. So someone who loves you, that anything whatever happens, they will always be there, so can talk to them. But you will tell them in bits, and tell them if it’s okay we can exchange experiences and they can support you, and when you talk to them, you will feel that some things have reduced so it will be fine.

**M: It will reduce stress for you.**

R: Yes.

**M: Have you ever experience?**

R: Yeah.

**M: And did you experience stigma after that?**

R: No.

**M: Have you ever disclosed your status to anyone?**

R: No.

**M: Boyfriend, girlfriend?**

R: We are waiting to finish school first.

**M: What? This is a safe space don’t worry.**

R: I know.

**M: You are waiting to finish school?**

R: Yes, and then we will look for love.

**M: Okay, so apart from xxxx there is no one who has disclosed to someone else, a friend or someone?**

R: Boyfriend.

**M: You have?**

R: Yes.

**M: How did he take it?**

R: He had to understand me, I had already got to know him, we had stayed 3 years, so I had understood him, so I had to tell him, because there is no need of me not telling him and then soon he regrets it.

**M: Mm, so he took it well?**

R: Mm.

**M: You are together or you broke up?**

R: We are together.

**M: Someone else?xxxx tell us anything so we hear your voice.**

R: Some are bad, you may tell them and then they go and tell their friends, and you will start to hear “that girl is dying”. So he will be telling people.

**M: So at least you avoid that.**

R: Mm.

**M: So is it normally like this or if you risk it things may go bad, or it’s not a risk to take?**

R: It’s not a risk to take.

**M: You just leave it.**

R: Mm.

**M: Okay and in the clinic do you talk about disclosing?**

R: No.

**M: So the day that you were told your status, did you have questions you wanted to ask but you were not able to ask? Let’s go round then, did you have any questions?**

R: Yes, to my parents.

**M: Mm.**

R: I asked my mom why she didn’t tell me before, but you know women, noise.

**M: She shouted?**

R: Yes.

R: For me they hid it from me for a long time, I found out on my own.

**M: So you found out by yourself, what triggered you so that you found there is something wrong?**

R: The day I came here and doctor was asking what my status was and ii didn’t know what status was. So I went to the doctor and asked what it was, but they had also lied to me a lot, so when he told me what it meant that is what I found out what it was and I found out my status.

**M: Mm.**

R: I started asking my mom questions, asking why I take medicine, she told me it was for HIV, I didn’t say anything.

**M: You didn’t have any questions even when you were alone?**

R: What I used to ask myself was I used to hear that is that it used to come from animals.(laughter)

**M: You never wondered how you got it from animals? Did you get an answer from those?**

R: No.

**M: You haven’t, hmmm. How did you find out?**

R: They used to lie to me that I don’t have it, but my health was not good, I used to have headaches, and they told me that it was for that. One day we came here and I was told that the virus was down so they decided to just tell me. So I went through counseling by the doctor.

**M: So your parents are the ones who used to tell you that it was for your head?**

R: I don’t know, I was very young then.

**M: Parents are clever.**

R: They used to tell me it was for my stomach.

**M: So then after he told you?**

R: I was fine because we had already covered it in school, so I just accepted.

**M: But like when you are at school learning about it, you don’t know you are one of them, how do you feel when you are told that your status is like that? What questions came to mind?**

R: I thought they were lying to me, I didn’t think it was true.

**M: Mm. It does not look real.**

R: Mm.

R: Before I was told, I was watching on TV World AIDS days and I started to say fukes I don’t have it. Then the next year when I was from playing I found mom at home and she told me to sit down and I sat down, when she was telling me I didn’t concentrate, so when she told me I didn’t ask anything. But I remembered what I had seen on world aids day and they said that first you get HIV and then you get AIDS if you don’t take medication. So I remembered that there was a day that mom told me that I had HIV, I wondered how I got it, I looked for support groups, I got to know more. I asked if there can be a way out, as an operation to remove the virus, but they told me that they can’t be seen. I also asked if they can inject instead of the drugs.

**M: So for you your biggest challenge was drinking the drugs.**

R: Mm.

**M: Have you asked your mom?**

R: No I have not.

**M: Mm.**

R: For me I didn’t react, because when I was told I thought that it was a sickness like any other, because I didn’t know it’s a case with many people, and there is a stage that you reach you get worse than it was, I got used to it. Then I was told that if you don’t take those drugs, either your viral load will go down or either the next stage, so you should take drugs. I was given courage by the people that I was living with.

**M: So at least family members know.**

R: Yes.

**M: Mm.**

R: Oh I didn’t know, I was young, so I didn’t understand anything. I came to learn with those pills, I hate drugs and I used to wonder why I am taking them. I saw my sister also taking them, so I was wondering why she is also taking them, but since she was younger than me I could not ask her. I finally read the package and saw what they were for and I just accepted.

**M: So no one told you?**

R: I don’t know, I can’t remember.

**M: So you came to figure out later what it really means.**

R: Yeah.

R: As for me, my mother feared telling me, so she brought me to the hospital, she took me to the counselor, and this counselor is the one that talked to me and I accepted myself, I felt that I was able to live like other people, I accepted my situation.

**M: You didn’t have questions that day?**

R: No.

**M: Even one?**

R: No.

R: I got sick with rushes and then I was taken to the xxx hospital and they told me to come here because they could not treat me there. So when I came here with my dad that is when they told me about it, but I was very young, I didn’t understand, I came to understand when I was in class 7 and I accepted my situation.

**M: How did you understand?**

R: My mom took me to VCT and I was told I had the virus, but I was told that I can live with it, so I just accepted.

**M: Mm.**

R: I didn’t have questions, I was told about it and I just accepted and life continued.

**M: Who told you?**

R: My mom, she took me to a counselor and they counseled me and I accepted.

**M: You just accepted?**

R: Yes what else could I do, you just have to accept that is your life now you just continue with your life and be positive.

**M: So when you have stress of any kind not necessarily about your status, any stress who do you talk to?**

R: Stress is for people with a wife and kids, no one here is married so we don’t have stress.

R: Washing clothes.

R: Dishes, you eat you wash the dishes, your dad cannot wash dishes, you have to do it.

**M: Come live with us, mothers like teenagers who work.**

R: You know teenagers don’t like to work, they just want to sit and have fun.

**M: They don’t want to work.**

R: Yeah, like some teenagers are told to take the cups in the kitchen and then they don’t do it, they wait until later after.

R: Instead of just picking it and continue watching the TV, some teenagers are just rude. You know if you are obedient to your parents, then they will give you more freedom, when you want to go somewhere you will be allowed. But if you don’t, they will remember what you did and refuse.

**M: So you don’t have stress? I have never heard of this, you even hear of teenagers killing themselves, that is stress right?**

R: Mm.

**M: You have gone and chatted up a girl and she has said no.**

R: You just move on.

R: Girls are many.

**M: But there is somewhere you will feel something.**

R: Yes, if you really loved that person.

**M: Even if it is a little, you will feel something.**

R: No.

**M: Exams, school, there is no stress?**

R: Yeah, because you will fail and your parents will ask you if you are going to make it if you fail.

R: Or they take away your phone saying that is what is ruining kids, you try to tell them that you are not like that, but they just won’t trust you.

**M: So when you have this stress who do you talk to?**

R: I pray.

**M: You tell god to talk to your mother?**

R: Sometimes you are talking to yourself and people think you are crazy because you are talking to yourself.

**M: Okay they think you have smoked bang.**

R: no but I am talking to myself.

**M: So you don’t talk to anyone?**

R: Talk to yourself.

**M: You don’t have bff?**

R: No we don’t trust them.

R: That is just a name to lie to them, that is not true, how do you become someone’s friend forever, it’s not possible, it’s just like now for like 2 years and then you are going to go to college and get someone else who you are going to call bff. When you work you will have another one so it’s stupid to have a bff.

R: But you can also talk to yourself, you talk to yourself and you solve that issue alone, like when I fail I start telling myself now you have failed, till my desk mate asks what is the problem and then I tell her that I am solving my own things, to leave me alone.

**M: You talk to yourself telling yourself what?**

R: I tell myself that I have failed it’s not the end, I tell myself there is still more time, there are still teachers we can ask questions, there is a reason that you have failed. I tell myself “you studied right? You know that you read right?” so it will be fine, but if you didn’t study you are punishing yourself because you know you didn’t study.

**M: You punish yourself?**

R: You tell yourself that you didn’t study.

**M: So when you punish yourself what do you do? You beat yourself?**

R: You sacrifice, that time people are having fun, you sacrifice.

**M: You punish yourself?**

R: If you can’t you tell someone who is close to you to punish you, as in if you are not strict yourself, you tell someone who is strict, when people are having fun you study, when it’s time to sleep you go and extend the studies.

R: Mostly chicks when they hear stories about boys, they jump in and they will talk and time goes by, especially now in January, no one will study.

R: We go to fests, events.

R: Yeah they know.

**M: You want to say something?**

R: When someone tries to tell you something you shut them up.

R: When they try to tell you a story you tell them to stop.

**M: So there is no one who is giving you an emotional support? I think it’s a different story for everyone, not everyone punishes themselves.**

R: No there are times you love yourself, you don’t have to punish yourself.

R: You have started to change?

**M: When you need emotional support, where do you get it?**

R: You know emotional, there is a person instead of emotional support they continue to hurt you, there is no need. So when you see people having fun, you have fun and at least the anger that you had will reduce as in then you will have a chance to talk to someone.

**M: You were saying?**

R: There are people you can’t tell anything, even those bff.

R: There are people who can make you mad.

R: Even at home people may bore you, but your friends will not be strict on you, you are all one age so you do whatever you want, so you have fun, but at home you will just stay in your room because you don’t want to bother with anyone.

**M: Okay.**

R: I prefer to keep it to myself, who will you tell? It’s better to be quiet things will be fine.

**M: Keep quiet?**

R: You know if you keep quiet you will have depression, so it’s better to let it out.

R: I think there are people just cry and are relieved.

**M: David, when you have stress, where do you get support?**

R: My dad.

**M: He listens to you?**

R: Mm.

**M: Derrick?**

R: I don’t have anyone, I just keep to myself.

**M: Why?**

R: I just solve it and life continues.

**M: Mm.**

R: There is a day my mom told me to stop thinking so much, like my mom noticed like I had depression, I am used to keeping things to myself, till that time my mom told me to stop doing that, but I didn’t follow.

**M: Why do you think she told you that?**

R: Maybe she is afraid that I will get depression.

**M: So she is afraid, but you think you are managing well.**

R: Mm.

**M: Ruth?**

R: I would rather stay with the stress or just go out and talk a walk.

**M: You stay with it.**

R: Yeah because if you talk to a friend and you are mad, you will be angry at them.

**M: And if it’s some other support that you need, any other kind of support, where do you get it?**

R: From my brother, sister.

**M: What kind of support?**

R: Like when I need something, anything.

R: Money (laughter)

**M: Where do you get it?**

R: You give stories to your parents and they give you.

**M: You have to give them a story to get support?**

R: Yes.

**M: Hmmm, someone else, Julia?**

R: You have family members.

**M: For which support?**

R: When you need something like money.

**M: So money is the only thing we would ask for or if you need something, but if you have stress you won’t talk?**

R: You can talk, but you know there are people you can tell and they think you are seeking sympathy. Like for me it’s hard for me to tell you something, unless it’s really hurting me, that is when I will tell you. If you have hurt me, I will come and tell you and we will sit and talk, but if we have a problem, I will tell my auntie, my sister, my brothers.

**M: So people will think you are seeking sympathy?**

R: Some, you know there are people who don’t have a heart.

**M: So and if its issues to do with your HIV status, where would you seek support?**

R: From someone who is like you.

R: Or the doctors.

R: You go back to the youth nurse.

**M: To the youth nurd=se.**

R: Yes.

**M: Even for you, from youth nurse?**

R: Mm.

**M: And the youth nurse is available always when you need her?**

R: Yes, you can come back, it’s not only today.

R: And if you have storo bonus you talk to her in the evening.

**M: Okay, there is no family member that you will go to?**

R: No.

R: Maybe a family member who brings you to the hospital, or a family member who knows about your status, not just anyone.

**M: So there are family members that don’t know our status.**

R: Mm.

**M: And do you feel that you are supported in the family? Hey , your mind is not here.**

R: I have drifted.

R: Or you are thinking of where you will cross over the year.

**M: Where will you cross the year from today, or there is a bash?**

R: Yes.

**M: Right now or at night?**

R: At night.

R: Kesha.

R: You can see how people are dressed here, the way they are, they are not coming back until tomorrow.

**M: Tomorrow morning.**

R: You can see the bags that are carried, there is make up.

**M: Do you feel that you get support from home?**

R: Not a lot.

**M: Not a lot.**

R: But you know you also need something that will keep you busy, as in something that will distract you, and most of our parents deny us.

**M: How?**

R: Like something that I like a lot is chatting with friends on the phone, but mom does not want this.

**M: You agree?**

R: I agree, you know when you take a phone and call someone, they tell you, “oh it’s that person that you are always with”. As in its like they don’t want you to call friends, they want you to keep calling them only.

**M: Mm.**

R: You know the reason.

R: You know all work and no play makes jack a dull boy.

R: That is not play, take a ball and play.

R: But a phone we are also not allowed to get a phone unless you are trusted to show that if you are given this phone there is nothing that you will do that is bad. You can’t just come from school and say you want a phone, check your grade first, if you are studying, you can’t just be given a phone.

R: Maybe you have a lot of mistakes, and maybe your mom sees that you see things that you are not supposed to see.

R: I think if they want to take it they can take it so I can study, but once I am done in November 29^th^, they should return it to me, I should not ask for it, they should just return it.

**M: Do we feel we are supported or just a little? In the family?**

R: Maybe someone calls you and it’s a friend, when you are about to go and pick, your mom picks it, and you wonder why. Even if you tell them who it is, they will take the phone and hide it.

**M: They will take the phone and hide it?**

R: Yes.

R: Even the line.

R: You go and renew.

R: You will finish school and you will have your freedom.

**M: Mm.**

R: Struggles are a must but you will manage.

**M: So support?**

R: A bit.

**M: Mm, xxx you are quiet, do you feel that you are supported?**

R: Not that much.

**M: Just a bit, how would you like to be supported?**

R: Freedom.

**M: You have freedom?**

R: My mom is tough, if you go out of the house a bit, my mom will follow you.

**M: She will follow you?**

R: Yes.

**M: You have to stay in the house?**

R: Maybe you can leave when she is not there, but when she is there, she won’t allow.

R: That is wrong.

R: Who will pay your school fees.

**M: Even today for the New Year’s?**

R: Today you will go by force.

**M: So the support that you want from the family is freedom?**

R: Yeah total freedom.

**M: Total freedom?**

R: Yeah they should give it to us so that they see where we can reach, because like now I have finished school, and my mom should not be stopping me from going places, I need to have fun, I need to go to fests sometimes, there are times that I need to go out. I decide to go out and my mom says “you want to go round the streets?” I wonder if she expects me to just stay in the house looking at her.

R: Like me for me I have watched all the movies, all the programs, cartoons, and we moved, so where we are there is no one that I know, I stay in the house, you have watched movies, you have finished until you are tired and you feel like you want to talk to someone. At least with the phone you can exchange thoughts with someone.

R: And you feel lonely sometimes, I don’t like this story of staying home, I like hanging with friends because when I am with my friends I won’t think, but when I sit at home, I just have stress.

**M: and you?**

R: I support her, staying home is boring.

**M: There are no activities that are at home?**

R: You listen to music, you go to you tube, like you have done everything in the house.

R: When she sees you go out, she is at the door looking at you, and you wonder how did she know? When she sees you looking at a girl she gets mad. Then the second day you will stay home, you get bored and then you wait for her to leave the house, when she leaves you say you are now free, so you start thinking where to go, then you go and when I reach there, she is there. She follows me everywhere like I am a girl.

R: And if you were a girl?

R: And you can’t know when you are being followed, when I look behind I don’t see anyone.

**M: How naughty are you? Is there something that you did that is why she does that?**

R: Maybe you came back home drunk?

**M: Was she like that from before?**

R: Yes.

**M: And when you talk to them?**

R: I tell her “mom, just let me be free I walk around at least” she then says “fine” and when you leave and come back, there are so many questions.

R: But I think you should not be harsh on your children because when you are harsh, your child will go and talk to someone else and they will leave you there, so you should not be harsh on them. You need to give them freedom, tell them “go and come back at this time” you also went through this and you wanted this freedom also. They also were having boyfriends, so why not let us also go through that.

R: That is what they say, they never did that, they used to only study, morning to evening all the time.

R: That is a lie (laughter).

R: That is what they say.

R: It’s just a lie.

R: It’s like they are the ones that used to study the most.

**M: They just used to study only?**

R: Yeah, that was their only job, they used to study and come back in the evening, even lunch they never used to eat. When you look at the belly you wonder they never eat?

R: Like here I can see people are stressed, I think she has not asked for permission for today, its already 12 noon, ask for it, when its 6pm they won’t agree.

R: You just go and come back later and get the punishment because you already had fun.

R: Its better you just ask for permission.

**M: So the parents should understand?**

R: They should because if you understand I will come back and tell you that it was like this and that. The next time you will tell me to and come back at a certain time. But if they don’t understand then you will go and come back when you want.

R: In whose house? (Laughter) go and sleep outside, you will find your things packed.

R: You will find your things packed outside.

**M: It seems that you are well disciplined.**

R: I can’t just go, I just ask for permission, when you are able to sweet talk them, when they say no, you lie about something, you even tell them that you will give them the number of where you will be, but it’s your other number that she does not know.

R: Yes, you put it on and when they call you change your voice.

R: Then you turn your phone and you cross the year.

R: When you turn off your phone then she will know that you are doing something wrong.

R: You tell her that the battery died.

R: There are times that they are so overprotective, like there is a day I had chemistry work and I didn’t have the book, so I was to go to a friend to take the book, I told my sister to give me the phone to talk to her because when I talk to her on the phone I am able to talk to her well, when she is there I cant. She said “you have started bad behavior” she thought I wanted to go out. Even when I came back with the book she had to check everything to make sure. As in I even have a problem, when my mom is here, I can’t talk to her, but when I message her, I tell her everything.

R: And those parents that don’t want you to go out for the book when you are chased from school they start giving you a lecture.

R: Yeah they tell you “why didn’t you write?”.

R: And they are the ones that won’t buy for you.

**M: So you need more support from parents.**

R: And freedom.

R: And they should understand more, like if you have finished school, why do they want you to stay in the house?

R: It’s not fair.

**M: So what makes it hard to talk to your parents?**

R: As in they judge.

R: They are quick to judge, they should know you and then judge you, but they don’t know you but they are quick to judge you until it hurts, that “what I have I don’t that my mom does not trust me?”.

R: We are not bothering her.

R: When you are going to a birthday, she follows you.

**M: She follows you and stays?**

R: She stays and takes the juice.

**M: Do you have other brothers and sisters?**

R: I have only my brother.

**M: Derrick you are so quiet, what do you think about this?**

R: You know I don’t like going out, even my parents ask me why I don’t go outside.

**M: They ask you why?**

R: Time is not yet.

R: Are your parents tired of you?

**M: Let’s listen to him.**

R: My brother has been given freedom too, he also goes out a lot, and he is the one who is indiscipline, he goes everywhere, he is seen everywhere, he is seen in Corner, he is seen here, and he is younger than me. So that is why they ask me why I don’t go outside.

R: I wish that was me.

**M: And phone?**

R: I don’t have a phone.

**M: But you prefer to stay in the house.**

R: Mm.

**M: and you xxx?**

R: I have freedom too, because I have my own house now I have freedom.

**M: You have your own house?**

R: Yes.

R: Call me we live, (laughter)

R: We pay rent?

**M: You have a house away from home?**

R: Yeah away from home.

**M: Why?**

R: He wanted his own house.

**M: You rent for yourself?**

R: They pay for me.

**M: Because of school or?**

R: Yeah.

**M: So you have freedom.**

R: Yea.

**M: Wow okay, so freedom and support from parents. And has anyone here ever experienced stigma at any point in their life?**

R: No

R: I can’t get it, I am strong.

R: You encourage yourself, they see that this one we can’t bother with her. I have almost gotten it but it didn’t get to me, some girl in school, you see when you have many friends and then this girl didn’t have any friends. So she started stories about me saying I am positive that she was told. When I ask her who told her, she won’t say, but me I am a don’t care, I don’t care about whatever someone is saying, I didn’t listen to her. We were in class and my friend told her “there is no need to bully someone and you don’t know the whole story, you should know the story and then come and tell me” from that day she never said anything else. She came to tell me to forgive her and we moved on. You don’t cry, because if you cry people will take advantage of you, you pretend that you have that strength to conquer them.

**M: Mm.**

R: There are those people who maybe the teacher was harsh on you or said something wrong, they come and tell you why won’t you react, but those people you just tell them to back off, they want to talk saying something to show that you are weak because you are not reacting. So you have to be harsh on them because there are people who are like that.

**M: They want to show they are stronger than you.**

R: And you don’t need them.

R: Mostly girls.

R: I even fear them, like you may pass somewhere and find them and greet one, and then you hear them say “why isn’t he greeting us?” the gossip that will be cooked there.

R: Girls are like that, they are envious.

**M: Mm, so we had said that taking the pills is stressful.**

R: Very much.

R: Especially for me.

R: I have a question, they can’t find a way of helping us? Because I hear that in the US they have that medication and they don’t want to give it to Kenyans, I don’t know if it’s true or not, I don’t know if it’s a lie.

**M: Mm.**

R: There are times when you feel like they don’t want to go down, you feel like throwing them.

R: Yeah throwing them away to the dustbin.

**M: You feel like throwing them away.**

R: They should bring that one you can be injected the whole year

R: Or four years.

**M: Four years?**

R: Yeah, you just come to the clinic.

**M: You will even forget.**

R: You will, they can tell us to meet and talk, that is fine but this daily one, no.

R: And they don’t reduce, they increase.

**M: They increase, for everyone?**

R: They reduce.

**M: They reduce, or they can be added.**

R: It depends.

R: When they reduce, then they come and add some more.

**M: Mm.**

R: But when you are healthy, and take your medication, viral load is low, then there are some pills you won’t be given.

**M: What makes you take the pills regardless?**

R: Because you know there is no other solution, there is nothing else you can do.

R: You have no otherwise, if you don’t take them, you will hurt yourself.

R: If you don’t take them, you will be buried alone.

R: We will come and eat meat.

**M: You will go to eat meat.**

R: No meat will be cooked.

**M: Meat will be cooked.**

R: Yeah.

**M: And is there a time that you feel like you don’t want to take them?**

R: Yes, they are tiresome, like you can go outside and play a lot and time passes you, and you forget.

R: You play and forget.

R: When you come back home and eat and sleep and forget to take them.

**M: You forget.**

R: Even when you forget, I was told that you should take them even if your time has passed, there is no problem.

R: And if you forget completely.

R: Maybe that is what you want.

R: I was told that if you forget for 30 minutes, that is okay or 1 hour.

R: But if you think that you won’t take for one week, you should be able to take once for 7 days.

R: No that won’t help.

**M: Do you do that?**

R: No I just wish.

R: Will they pass your throat really?

R: They are too many.

R: They are too strong, it can’t help you to take them at once.

**M: That’s how bad you don’t want to take those pills.**

R: Mm.

**M: Which other situations make you want not to take the pills?**

R: Say you are in a bash and then you are busy.

R: That is when you forget.

**M: What are you doing?**

R: You are dancing and you forget.

R: You can’t remember.

R: Your mind is with the music and friends.

**M: And you ?**

R: I find it hard to take them in the morning, I feel as if they are choking me, but I have to do it.

R: Do those drugs make you sleep a lot?

R: Yeah.

**M: They make you want to sleep?**

R: Yeah, especially during CRE.

R: And math.

R: After lunch.

**M: I want to hear your story its interesting.**

R: I was saying that they should change the medication.

R: I even don’t think I will be able to take them today because of that bash, I will have to carry it.

R: We are going to a wake, not a bash.

R: I am going to a bash.

**M: You were saying?**

R: I want the doctor to change my medication because I used to take ZDL which is take at 6:30, in the morning and evening. I told him to change it and he gave me one for 9pm, and it helped.

**M: That helped?**

R: Yeah.

**M: Because you didn’t want to take them?**

R: Because I used to forget, but with that my viral load was not increasing, it was just zero.

R: You are like me, I also forget but I also remain zero.

R: I have a small brother, I don’t know the difference, I can forget even two days, not now they have changed for me, the morning and the next day. My brother takes everyday but his virus increases. So I don’t know what the difference is.

R: Maybe he doesn’t eat.

**M: And yours does not increase even if you don’t eat?**

R: Yeah its always zero, when I am tested its zero.

R: Maybe the food he eats.

R: But they eat the same thing.

R: And drinking water.

R: That day we were told that we should drink a lot of water because the side effect is that it drains your water.

R: Which drug?

**M: Septrim.**

R: You should drink it in the morning.

R: Mm.

R: If you take it you take it, you drink with a lot of water.

R: I tried to take it in the morning and I could not I take it at night.

**M: So we avoid taking the pills when we, first when you are bored with it, taking it every day.**

R: When you don’t feel like it.

**M: Taking it with a lot of water, or when you are in the bash.**

R: Yeah.

R: Should you take the drugs after eating or before?

**M: What do you think?**

R: After.

R: Anytime.

R: You have to eat , you can’t take them when you are hungry.

**M: So you have to eat first.**

R: Yeah.

**M: What can make life easier for you to the extent that taking the pills will be easier?**

R: Be injected.

R: Or be liquid.

R: Liquid is worse, because someone else will be worse because someone else may come and take it. It rather be injection so that it stays in your body.

**M: If it’s a liquid someone else will take it?**

R: How will they know? When they ask you, what will you tell them?

R: They won’t know.

R: They will see the name of the drug and they will go to the internet and they will see it. That is the problem, you see like these ones you can just put them in those small papers and no one will notice. But he liquid someone will see the bottle and they will go and search.

R: But the liquid one someone will not forget to take.

**M: You think that with liquid you won’t forget?**

R: Yeah and you can tell people its mouth wash. (Laughter)

R: Something you can take once.

**M: Are there any other people in the family who are taking the drugs?**

R: No.

**M: You are the only one?**

R: Me and mom.

R: Me there is.

R: Two of us.

**M: Sisters.**

R: All of us in the house.

**M: How many?**

R: We are five, but one of us, our last born is the one that is negative.

**M: Mm.**

R: Even our last born is negative.

**M: Mm.**

R: Yeah.

**M: How old is he?**

R: There is medication that is there, like before a child would be born positive like me, but he was born negative.

R: Right now you can get a negative child.

**M: Mm.**

R: Yeah because when you are positive, you would not want your child to go through what you have gone through. So people should be taught what they can do. Because I would not want my child to go through some things in this life.

**M: So you will work hard.**

R: Yeah for them to be negative, because I don’t want to answer some questions.

**M: How do you feel, do you feel that when you have kids, they will be negative?**

R: Yeah.

R: Dream so.

**M: You dream so?**

R: We are not dreaming.

**M: Is it a dream or its real?**

R: Its real.

**M: Is it possible?**

R: Yeah.

**M: You think its impossible?**

R: I don’t know.

R: Its real, I believed it when our last born was born negative and we are all positive, so I believed that it can happen.

**M: Because you can see, what do you think?**

R: I don’t believe it.

**M: Why?**

R: If they are positive what will you do? Will you kill them?

R: You are the one who will be depressed.

R: You will be the one to tell them.

**M: Mm.**

R: No let them grow up negative, I would not want to leave them that stress.

**M: You don’t want to pass your child that stress.**

R: Yeah what will you tell them?

R: Questions.

R: Children of today have so many questions, they ask so many questions.

**M: Mm.**

R: I don’t want questions.

**M: You think about the future, when you are married, family, things like that?**

R: We are yet to think about it.

**M: You are.**

R: I think about it.

**M: You have decided not to think of it for now.**

R: We are in school.

R: Right now it’s just having fun, you are just playing with them.

R: But if you are found, you will know you don’t know.

R: At the wedding another girl will come to the wedding with a child.

R: The pastor will be shocked even.

R: You play safe.

**M: You play safe.**

R: You are careful.

R: There is no need to be with a commitment with a girl, you just enjoy life, a girl will come and eat your money.

**M: What are you planning with it?**

R: You also want to have fun with her right?

R: Yeah.

R: There are men who want to spend money, you go to an event and the entrance is 1000.

R: You have to pay.

R: No stay home.

R: You said that you want to take me out, so.

**M: So as you are growing up, what do you feel about moving to an adult clinic?**

R: It won’t be bad.

R: Youth or adult? I would rather stay here, I don’t want, I would rather one for age mates.

**M: Age mates.**

R: Adults are old some of them.

**M: At 18?**

R: You sit with 18 to 20.

R: There should be teenagers, youths, and those older people on their own. If I have to go a group and there are older people I won’t be comfortable.

R: If you are in a line with an older person, you will allow them to go first, and if there is another one you allow them until its late.

**M: Mm.**

R: Just put youths together and adults together.

**M: So want to remain with teens?**

R: Yeah.

**M: And if you reach 19?**

R: One for youths.

R: And one for older people.

**M: Okay and is there information that you feel that you need that will help you live a healthier life, information that you have never hard?**

R: If there is hope for a cure. (Laughter), they should investigate.

**M: Which other information? David.**

R: The one for the cure.

**M: Mm.**

R: Me too.

R: Me three.

**M: So it’s about the cure.**

R: Yeah, that is what we want.

R: Those pills are irritating, taking them is a problem, when you are going somewhere you have to take them.

**M: Mm.**

R: They have side effects.

R: When you think that you will take them until you get old, its stressful.

**M: Mm.**

R: There is a day I was given and they all poured down.

**M: What?**

R: There is a day I had taken them from the hospital so when I was coming back home, I had forgotten I had not carried a bag. When I was coming home, you see those bags that are being sold now, I put them there, and when I got into the mat, all of them fell down. I was embarrassed, people started picking the pills for me, thank god I was at my stage.

**M: People helped you?**

R: Yeah, I didn’t look at their faces, I pulled my hat down.

**M: Mm.**

R: Even at school during inspection, I prefer to be last so that the teacher does not ask me so many questions.

**M: Oh.**

R: If a teacher hates you, they will embarrass you.

**M: They inspect the drugs?**

R: Yeah.

R: When a teacher hates you, they will keep giving you that look.

**M: What are you saying?**

R: We are saying that if a teacher hates you, they will want to know what these drugs are for, but for me I just tell them “they are for taking”.

R: You just tell him it’s for drinking, I can give you any answer, if they ask you “why are you taking these drugs?” “Because people take drugs”. (Laughter)

**M: What?**

R: Because you are sick.

R: By the way you should show them that you don’t care what they are saying, because if you care they will start to…

R: Or you tell them “you have never taken drugs?”

R: Like when they are ask where you are from, you tell them up and down, because if you show them that if they ask you something you answer well, they will embarrass you.

R: You should answer them in a way that they will never ask again.

**M: Mm.**

R: And don’t hide those drugs, put them there, if they want to know open the box for them to see, they won’t bother, but if you hide, they will wonder.

**M: They will wonder.**

R: Yeah.

R: When we opened school, I put them on the locker and my desk mate asked me “are they for HIV?”

R: When they ask like that, you tell them, “Yeah is it an issue?” (Laughter)

R: Is it an issue?

R: Yeah I think I would say that.

R: Yeah I said that once.

R: Some ask, “These drugs never finish?” you tell them “use your brain, can someone use the same drugs every day?” (Laughter)

**M: The ones from the other day are finished.**

R: If someone asks you, you don’t think about it, if you start thinking, they will think there is something there. You will be asked “what are these drugs for?” you say “For taking” “when will you finish”: “the day the doctor says so”. (Laughter)

R: They will have to leave you alone.

R: They will think it’s something else.

R: That is when they will apologize and tell you “no I wanted us to go to the canteen. (Laughter)

R: Then you tell them “did I tell you that I wanted to go to the canteen”.

R: Never hide, put them there.

R: They are even used to it now they ask me “you haven’t taken your drugs?”, I just keep quiet.

**M: They ask you?**

R: Tell them “you think I don’t know?”

R: You tell them that they keep quiet.

R: Like a friend of mine I was in the mat with, he would hear the drugs making noise in the bag when we hit a bump, he asked me what those were, I told him “they are for worms”. He said “let me see them” I told him to go where he was going.

R: You should have given him, now he suspects you.

R: If you would have given him, he will have to let it be, he will think that it’s not what he thought, he thought they were for HIV, but if you give him directly, he will think that they are for whatever you told him

**M: It’s a challenge, but I can see you handle it well.**

R: You have to

**M: You do.**

R: Long as you are alive.

**M: Are there other topics that you feel that are important that you want to know? Something that can support you in your life?**

R: People say that when you have HIV, you become very skinny like a tree, but it’s not true.

R: Just at these people here, are they skinny? No, can’t you see these ones are fat.

R: It’s those ones who don’t take the pills well.

R: On You tube I saw a girl who looked bad, she was in the ward being washed,, she was so skinny that you could see her bones.

**M: What was the issue?**

R: She was not taking pills, she had HIV.

R: I think HIV is better than cancer, with cancer, you have to cut your hair, look bad.

R: But at least with cancer you can be cured.

R: Unless it’s too late.

R: But can you stay with cancer if you know you have it?

R: If you know late, you will just die.

R: But with AIDS you can live for long.

R: You can live for long.

**M: Mm.**

R: If its cancer you would die.

R: The drugs should be sweeter.

R: But I don’t like sweet drugs, I will puke, like there are those I was given for vitamin A, I felt like puking, it tasted bad.

R: Some you are told to chew.

R: There are those which are oily.

R: I wish we could sniff it.

**M: Which you can?**

R: Sniff.

R: If you go to a party you just sniff.

**M: You won’t be high?**

R: No, even if you are high people won’t notice.

R: People will be like “these ones are used to be high” (laughter)

R: That is better because no one will notice; people will think it’s not that.

R: They will think its shisha.

**M: Where do you see yourself 5 years from now?**

R: Very far.

R: Superstar.

R: Wonder woman.

R: Super girl.

R: You should just stay with god, where he will take you.

**M: Where do you see yourself five years from now?**

R: Rock star.

R: I will be a footballer, you will be seeing me on TV, I have a passion for football. I am wearing a stretcher, I am just waiting for a game, then at night I cross the year. You know I have to look for money, you heard that girls are waiting to eat our money.

R: Be ready to spend.

**M: Ruth, where will you be in five years?**

R: Very far.

**M: How far?**

R: You have left Kenya or? (Laughter)

R: No, maybe I will have my house, a job, family.

R: Family? That is 2026, you will have a family?

R: Yeah.

**M: Julian?**

R: I want to achieve the goals that I have set now.

**M: Like?**

R: For school, pass exams.

**M: Okay, five years from now? Louise?**

R: Achieve my goals.

**M: Like?**

R: Have a car.

R: Like?

R: Lambo or a Bugatti.

R: Wish (laughter)

R: First I will go to US.

R: No tuk tuk.

R: Or bike.

**M: Mm.**

R: Have a job, a car, help my sister.

R: Amen.

R: And live a good life and have freedom that I want, just that.

**M: Mm.**

R: For me my goals to achieve them, like being a doctor, even if I will study many years, because I have been dreaming of.

R: Inject people?

R: Yes, and know more about art and design.

**M: Hmmm.**

R: For me I want to achieve my goals as in to have 6 digit salary.

R: Oh, you think you are Kenyatta?

R: And I have a dream to cure a disease, any, I don’t want to say HIV because I want it to have been found by that time. And go to a good school and be taking myself to campus with my car.

R: Amen.

R: And have a good life, and freedom.

**M: Freedom is important.**

R: Yeah.

**M: Let us hear from someone with freedom.**

R: I want to be a celeb.

R: You can join me.

R: How many followers do you have on IG, search, its 11.1k search me.

**M: 11.1**

R: I don’t know if I should show you, I am almost 12k

R: I want to do law.

R: Like me.

R: You didn’t say.

R: I want to be a surgeon and draw.

R: Like me.

**M: You all want to draw, be doctors.**

R: I want to model.

**M: What is your dream?**

R: MP

R: He will be a chief, a village elder, he says something and people follow.

**M: You want to be an MP.**

R: Politician? No I can die any time.

**M: Do you think there are challenges that will stop you achieving those dreams?**

R: No.

R: Maybe if you refuse to study.

**M: And any challenge that is associated with your status?**

R: No.

**M: None.**

R: Mm.

**M:**

**M: Okay thank you**

**End**
