# Supplementary material for: Psychosocial needs among older perinatally infected adolescents living with HIV and transitioning to adult care in Kenya
Source: PLoS One. 2020 Jul 29;15(7):e0233451. doi: 10.1371/journal.pone.0233451 (PMC7390380; doi:10.1371/journal.pone.0233451)
Supplement: S1 File — (ZIP) [file pone.0233451.s002.zip › uploaded final PLOS/Reviewed Transcripts/FGD 1.doc]

**M: I will request that we speak one person at a time during the discussion and then which language are you comfortable in?**

R: You can use both.

**M: Okay and my first question is I would like you to tell me about your experiences in this clinic, how has it been?**

R: It has been good, ever since I started taking medication in this facility, I have not faced any challenges between me and the staff they are all supportive.

**M: How long have you been coming here?**

R: Since 2004.

**M: Anyone else who would like to share their experience?**

R: My experience is same to (name) I have been collecting medicine here since 2005, and the staff are very good and even the counseling is good and we are treated well I have no complaints.

**M: What do you mean by good experiences?**

R: The way we are handled here is not the same way you could be treated elsewhere. Then, the doctors are caring and then in terms of medication they always have medicine and I have never come for medicine and I was told there was no medicine and I had to go and buy it on my own.

**M: Anyone else? How long have you been coming here?**

R: I have been coming on my own since 2014 and I came to learn what I used to come for in this facility for all those years last year and I started with this ARV medication in August. But I would say the doctors really encourage you and if you interact with them and share with them your situation they are very understanding and they are willing to help and they are also kind.

**M: So the doctors are good and they treat you kindly and they understand?**

R: Yes.

**M: Okay.**

R: And if you forgot your appointment they will link you and even call you to remind you that is one quality I really like about them.

**M: They call you?**

R: Yes, that is what I really like.

**M: Do you always forget?**

R: Yes, I do forget and they call me and remind me and I come.

**M: Okay.**

R: But of course you cannot lack demerits I have an experience of a doctor who talked to me badly and she was addressing me as if I was her child and I didn’t like it. I ignored it and it’s a by gone but I didn’t like it.

**M: Was it a specific doctor?**

R: Yes, it was a specific doctor and I mastered her face.

**M: What do you mean by her talking to you like you were her child?**

R: She was very rude towards me and that day I had just moved from the first phase of my medication to the second phase and I think she is the one who messed up because I thought I was taking the medication as prescribed but she was just addressing me badly.

**M: Did she assume that you were not taking your medication as prescribed?**

R: Yes, I think she felt that way which was not the case.

**M: Anyone else who would like to share their experience? (Name)**

R: My experience has been good and they are listening.

**M: Do you want to add anything, I saw you nodding when he mentioned that?**

R: Yes, I did they do remind us because I remember them calling me one time to remind me about my appointment when I was in school. Basically they called my aunt to ask her why I had not gone to the hospital and I really liked that, I felt like it was a really good gesture.

**M: So at least they do follow ups?**

R: Yes and I feel like they care.

**M: And thinking of the first time you learnt about your status how was your experience?**

R: I used to come to the hospital but initially I thought that I was just bringing my mum to the hospital but at some point both Lillian and my mum sat me down and explained to me what my status was and the reason why I was coming to the facility. I remember initially I used to take only Septrin and I didn’t mind since I was just taking a tablet and I thought I would handle it, but in August I was introduced to the ARV’s and I got confused because I was thinking that if I can survive taking the Septrin then why not continue taking them? And so that is when fear came in thinking that taking the medicine all the time was going to be tiresome. My other challenge was the fact that I don’t spend most of my time at home, at times I go for camping and I kept wondering how I was going to be able to handle the issue of taking that medication daily while on camp.

**M: During the period you used to take Septrin you never understood why you were taking the Septrins?**

R: No, I didn’t understand the reason why, but I remember that every morning when my mum woke me up she would tell me to take some medicine and I did without asking her the reason why. So one time my mum was told by Lillian to bring me so that I could speak to her because previously whenever I would come she would attend to me coincidentally and then after the counseling session I remember going outside the door and I read something like CCC and my mum explained to me what that meant.

**M: Anyone else?**

R: There was a time I was sitting with my mum in the house and then one time she asked me that (name) do you know the reasons why you take medicine daily? And I told her no and she asked me if I knew about HIV and I told her yes. and then she told me I know that you are going to blame me over what I am going to tell you but it’s time that you knew what is going on and then she told me that I contacted HIV through her during birth but I didn’t think it was a big deal since I was already used to taking that medicine every morning and every evening and I realized that its already part of my routine and there is no need of getting angry at my mum and blaming her and so I continued taking medicine and my life continued and its now my normal.

**M: There is no day that you got tired of taking the medicine and you felt like you needed a break?**

R: I have never felt that way.

**M: Anyone else?**

R: I knew about my illness last year and then when I came Lillian asked me if I knew the reason I came to the facility and I told her no. and she explained to me about my status and I didn’t think of it as a big deal since I was already used to coming here and taking medicine. And she told me that if I continued taking my medicine diligently then I was not going to face any challenges.

**M: When you say it was not a big deal are you referring to the issue of taking drugs or your status?**

R: The continuation with taking the medicine since we were already taking the medicine. The only difference was I now confirmed that I was HIV positive and I was counseled and now I have more knowledge of my status and nothing was going to change not unless God helps us and the manufacturers and scientists come up with a permanent cure.

**M: And you decided to move on with your life?**

R: Yes.

**M: Anyone else?**

R: Initially when I was young I was told that the drugs I was taking was cure for pneumonia and one time I asked why I had to take them daily and I was told that at some point when I feel better I would stop taking them but I never stopped taking them. And then in class 3 I asked again, in class 4 I asked again but in 2010 after FIFA I was told and I kept wondering how a young man like myself even contracted HIV. So I just adapted and decided to focus on my studies but I have faced many obstacles like ensuring that I take my medicine on time and also keeping nosy friends away because most of them cannot shut up once you share such an issue with them. I am not sure how some will react, are they going to treat me differently now that I am HIV positive**?** Are they going to shun me so all these thoughts came rushing through my head? So I didn’t want that stigma and I was forced to adapt.

**M: How old were you when you were told about your status?**

R: 10 years and I was just wondering how I am going to live with it and where did it come from?

**M: In your opinion do you like the way your status was disclosed to you or would you have liked it to be done in another way. Who informed you?**

R: My parents told me.

**M: Would you have liked your parents to tell you of your status or would you have preferred it to be done by a counselor at the hospital?**

R: I prefer being told by another person I trust them more and I wouldn’t want them to tell me through another person that would have been a total stranger to me that I could be seeing for the first time. My parents are people who have raised me and I trust them so I prefer them over anyone else.

**M: Okay.**

R: I was taking the medicine already so when I was told about my status, I didn’t mind since I was already taking medicine and I would just continue. But I have one concern because during advertisements for testing I see them showing the results to the person tested but I was never shown that so initially I used to feel that I was just taking medicine but I was not really HIV positive**.** And I have noticed that whenever I come for the CD4 checking every time the results are just undetectable whether I have been consistent with the medicine or not. At this stage of my life I would like to be given more details of what is being done to my body because my parents disclosed to me my status but they don’t have the evidence.

**M: You want to see the evidence?**

R: Yes, rather than just being told that tests are being done at the comprehensive center and I don’t have evidence even after coming all this way. I would like to be personally shown what is going on, but even though I have grown with the disease but I need more evidence though I have grown up taking the medicine and it has not caused me any harm.

**M: Yes, (name) share your experience?**

R: I used to be given medicine by mum and she would come for counseling with me and I would hear what the counselor was saying but I was not thinking it was intended for me. But eventually the counselor told me about it.

**M: Is that how you would have preferred to get the information or would you have preferred another way?**

R: I would have preferred that my parents told me about it in a cool and collected manner.

**M: Yes, (name)**

R: I don’t mind the way it was communicated to me, I am okay with how things turned out.

**M: How did you feel when you knew about your status? What emotions did you have at that point?**

R: At first when my mum told me my status, I didn’t know what to do, I just went out to play football then I came back and I asked her now to clarify what she had told me earlier on in the day. And then she told me about it and that was when I really got emotional, but just like all things it came to pass.

**M: Then she explained everything to you?**

R: Yes and at that point I knew things were serious and I didn’t even go back to play football.

**M: Anyone else, how did you feel when you knew about your status?**

R: I didn’t have any feelings because my mum already had told me about the medicine I was taking and she told me that I will be given more detail at the counselors office and I should not over react because it was not her option to get infected as well.

**M: Any other feelings?**

R: I didn’t feel bad because when Lillian explained to me, she started explaining with my parent’s status and then the medication and by the time she got to me I was even tired of the stories.

**M: She stared the stories from far?**

R: Yes and by the time she got to me I really didn’t care, initially I thought that HIV positive were all slim and unhealthy but looking at me I was so healthy and I knew that if I take the medicine then my health would continue to improve and I had nothing to worry about. And that made me feel good because nobody was going to easily notice my status not unless I told them.

**M: How did you feel when you knew about your status (name)?**

R: I was young and in school and I got no feelings I continued taking my medication because I used to drink it after school and I knew that was the path that I was going to continue with.

**M: Did you have any questions you wanted to ask but decided not to ask?**

R: My question was how my parents were infected by this disease and at one point I even asked my mum how she contracted and how I was also infected. I remember asking her while at the hospital didn’t the doctors do what they were required to do to prevent the transmission to me? And the funny thing is that my big brother is not affected he is HIV negative.

**M: And you keep wondering how he is HIV negative while you are HIV positive?**

R: Yes, those are the questions I had.

**M: Did you ask all those questions?**

R: Yes I did and I got questions.

**M: Did you have any questions you wanted to ask but decided not to ask?**

R: Yes I kept wondering why I was taking medicine all my life and I have not been tested and even shown the results. I wanted to ask my mum and I through that if I asked her she might find it rude, though I am used to talking to xxxx and xxx and	xxxx and

**M: Why didn’t you ask the counselors?**

R: I wanted to but I have never had the courage to ask them.

**M: Would you like to be tested still?**

R: Yes, I would like to be tested.

**M: (Name) did you have any questions you wanted to ask but decided not to ask?**

R: No.

**M: You didn’t have questions you just decided to empty your brain and move on?**

R: Yes.

**M: Is that true?**

R: Yes.

**M: (Name) did you have any questions you wanted to ask but decided not to ask?**

R: I have never had an opportunity to ask but if I did I would have wanted to ask what happened to me? How come my other siblings are HIV negative? Does it mean they don’t like me? Those are the questions that kept lingering in my mind. I remember I was very jealous at some point and we used to fight with my sister all the time because she is HIV negative, while I am HIV positive but we have now moved on from that and we are close. I remember at times she would pressure me to drink the medicine and she would really get me so upset I would ask her to take the medicine if she wanted to and we would disagree a lot.

**M: When your status was disclosed to you, is there anything you wish it would have been addressed but it was not addressed?**

R: I would have just know how I contracted the virus.

**M: Any other issues?**

R: More information like the consequences of not taking drugs, like if you fail to take the drugs and your CD4 increases then you will be moved to another class. Like I was not willing to switch to ARV’s because I was already used to taking Septrins and I was okay. So why introduce me to ARV’s that really affected me, I felt like the Government was forcing everyone to take ARV’s.

**M: Were you told it was a Government directive?**

R: When I came here I was told that it was a Government directive that everyone should start taking the ARV’s and I was wondering why it was a big deal while I had suffered from the same disease but I have survived on Septrin for a long period of time. When I went to Lillian at some point I felt as if I was the only one who was still using Septrin while everyone else was using ARV’s. So even Lillian called me and she was asking me how I had survived by only taking the Aspirin.

**M: Were you given any reasons why you had to use that medication or were you just taking?**

R: I was not being told, but I just took them so that I could recover from whatever ailment it was and I was told that I had to take them to be able to rise the following day.

**M: Have you ever tried not to take the medicine and see what happened?**

R: Not while I was young because of that fear of not waking up, I actually used to go and show them the pill before taking it, just as a confirmation.

**M: So at times your parents tricked you into taking medicine?**

R: Yes.

**M: Any other trick they used?**

R: That was the main one and as a result I was just afraid of not waking up the following morning so I would just take the medication.

**M: And then since knowing you** HIV **status how have you coped with your status emotionally?**

R: First of all I made a decision to be happy to keep my sanity so I joke a lot in a way that if someone was told that (name) is HIV positive nobody would even believe it. I decided to be very busy, I organize funkies and no one would easily believe if they were told that (name) was HIV positive**.** I feel that as long as my friends don’t know anything about it, then I will be okay. I know that there are functions then I can attend go have fun and not put myself or anyone else at risk but I fear that if I tell my friends then they will start sidelining me and they might exclude me from certain functions.

**M: You feel that if they find out you might miss out on some functions?**

R: Yes, and they might even fail to inform you because they fear that during the functions you might have sex with the same girl and they might be affected and they might blame you or even arrange for your death. So I prefer controlling myself and not disclosing my status especially to my friends.

**M: Anyone else how have you been copying?**

R: I have also been copying well but funnily enough just like him, I wouldn’t want some friends to know because they can be your friends but also your enemies once they find out.

**M: Why don’t you want them to know, what are the challenges?**

R: There are friends who have never understood about Aids and they feel that being HIV positive means a total death sentence, they have never understood that HIV Aids is just like any other disease with the medication though you have to take it daily but it’s just like any other disease that if you take the medicine you can stay for long. So the reason I keep it from them is once they know then your relationship with them might be over. So for that reason in my circles only five people know my status and none of them are my friends. I wouldn’t even want any of my friends to know.

**M: Who knows your status?**

R: My mother and my father and other siblings.

**M: Okay.**

R: The other reason I don’t talk about my status is that maybe you might have told someone and then you disagree and this person is hyper and he or she might just tell everyone maybe in a social place or during a function and that might demoralize you. But now if we disagree that person has nothing they are holding me for and we can talk about any other things but not my health, but if they know something they might even push you to kill yourself due to the stigmatization.

**M: Because they will be using that?**

R: Yes, you might find that even if you were in a relationship with a certain girl that you had not disclosed your status to, or you were in the process of doing the same they might go and tell them but they might not do it in a good way, they might do it to show that you have probably come to infect this specific girl and “kill them” and they might use that disease to black mail you because they know that if you don’t do what they want then they can go and tell everyone about your status.

**M: What are the chances that your friends are HIV positive? Because by looking at someone you cannot just know that they are HIV positive or HIV negative?**

R: I read on the internet that one out of ten people are HIV positive so it’s not that none of them are not infected but one are HIV positive but they have also not told other people.

**M: But why is it a big deal for your friends to know your status?**

R: It is just what we told you, I fear that once they know then I will not be able to join them for the fun and that is also important for me to socialize with my peers. I will just be lonely there with my family all the time and I don’t want that I want to live.

**M: Any other reasons?**

R: I know that we are many because even in school there are people who throw away those cans after using them and by looking at them since I know the packaging I know that there is someone else but I don’t know the identity of this people. So in that case I cannot just trust anyone but recently when I came here I met one of my school mates he in form three and I was really shocked and it made me realize that I was not alone.

**M: Did you talk to him?**

R: Not much but I was happy that I at least know someone and I am not alone but Joyce connected us and told us to be helping each other while at school. And since that time we have been supporting each other a lot but while in the estate I am just a regular boy and nobody can notice anything. I take my medication very early in the morning and late at night so I am good and my life goes on

**M: In your opinion have you seen anyone dealing with the same issues that you are dealing with maybe they encourage you when you see how they manage the disease?**

R: My parents and then there is this speaker who attends most talks and he openly talks about his HIV status and how he has managed to live was normal life with the disease for long. I don’t know his name but he is a motivational speaker and he motivates me a lot. I admire them but I don’t think I have the guts to stand up and tell a whole congregation about my status, maybe in the future but not right now. I currently feel that I am a stage where I will just deal with my status and if you know well and good if not then I will not be the one telling you. So mostly my parents because they have encouraged me that I can live even much longer being HIV positive and I don’t want to tell everyone because it’s not like they will help me in any other way other than giving me those judgmental looks and that is what I dislike. It’s not something like cancer that has no cure, it is something that if I take medicine then I can live for long till my dying day.

**M: But would you encourage anyone if they approached you?**

R: Yes, I would but I would not want to act as the expert because you might get people to start suspecting you.

**M: Anyone else?**

R: My mum because she has really been committed to her medication, she carries them everywhere she goes and she doesn’t miss taking her medication. And she has a piece of paper that helps track how she take her medication and that has really helped her a lot since she walks around with water and medicine and when the time comes for her to take the medicine he takes it regardless of where she is.

**M: Any other person?**

R: I know a certain actor who acts in Real House helps of Kawangware I don’t want to reveal their identity but when I heard about it, I was really encouraged because that is a HIV positive thespian but they are really good in their craft, having a good time doing what they like and making money and it has not affected them at all and I feel that I can do the same as long as I follow my prescription.

**M: Any other person?**

R: My mother just like she said her mother always has her drugs in her handbag and never misses a day but it’s a challenge to us men because we don’t walk with handbags so it’s tricky carrying our medication.

**M: But you can carry your back pack I see many men carrying their back packs?**

R: That is tricky with all these AP’s harassing youths; you never know when they might stop you asking you what is in the back pack. Actually I don’t even like putting on caps while at home due to the same reason of the AP’s harassing us, they are always seeking for bribes and when the see you with a bag even if they don’t find anything suspicious inside they still expect you to bribe them and I don’t work, I don’t have money to give them because I also rely on my parents to give me money. If you don’t have money they can even drop two rolls of bhang in your bag and accuse you of taking bhang and that story might turn out to be what it was not initially. There was a time I had earphones in my pockets and they stopped me and accused me of being a rapist and I was locked in and my parents had to come and have me released but I was actually coming from church. I remember being the only one that was released without giving them any cash because my dad was really upset because of what they were trying to blame me over and

**M: Any other person?**

R: I went through the same experience he did, we had come from overnight prayers and were walking back home and I was stopped by the police and we were accused of carrying drugs, like cocaine, heroin in our bags and so my dad was informed he came and he refuted those claims as they were unable to produce evidence but they just accused us of smelling bhang and those other drugs and eventually the case drugged on for some time but we were eventually released and the charges were dropped.

**M: Were you released immediately?**

R: Yes, but the investigation and the case went on for a while as my dad pursued the case further because it was a case of misinformation.

**M: Okay.**

R: I was also harassed in almost a similar case and that was as a result of that location I was, it’s just a certain location that most youths normally eat and so the police came and we were arrested and taken to Jogoo road police station and unfortunately I don’t get along with my step dad so he just like supported the police accusations that I take a lot of bhang and so I ended up spending a night at the police cells and I was released the following day due to lack of evidence. I would have gone to industrial area but I was lucky and I totally felt betrayed but that is life.

**M: So if you are arrested and you end up spending time in the cell then you miss out on your medication?**

R: Yes, I do.

**M: So carrying bags, and caps and hood for boys its dangerous?**

R: Yes it is.

**M: Whom do you talk to when you need any other kind of support not necessarily emotional or HIV whom do you rely on?**

R: I talk to my mum and my childhood friend, though we have been friends for long since we were young I can tell her anything but not my status.

**M: You have not trusted her enough to tell her your status?**

R: Not yet.

**M: What are your fears?**

R: I cannot even tell her, never thought about it, never will.

**M: Anyone else?**

R: I would talk to my mum and my aunty, only those two are the people that I can trust with any type of stresses that I am going through in life.

**M: Even if it’s about your relationships?**

R: No, that I cannot talk to them about it, I deal with that on my own. I will just sit down and talk about it and if its anything else maybe you insulted me I will insult you back or even use your insult to insult you and it might make you feel really bad about what you just told me and luckily or unluckily I don’t let anyone tear me down, I am very resilient and I really protect myself.

**M: Who else would you talk to?**

R: In school there is a certain accountant that I am very close to and another lady that I can talk to but I would not approach them to talk about all my problems, just specific ones, and then my friend at home, but there are specific issues that I can talk to with my dad when I know that he will not lecture me about them that is when I approach him. There are times I tell my mum something’s and she ends up telling my dad and things really blow out of proportion so it depends on the issues and there are specific things I tell my friends, some the accountant and then others my parents.

**M: How about you?**

R: There is no one that I trust and I prefer to keep my problems to myself because I feel that all they do is tell me sorry but they don’t do anything about my problems.

**M: You don’t feel that by someone telling you sorry that helps?**

R: How does that even help me?

**M: Don’t you think that by talking to someone at times it helps to ease the pain or that doesn’t apply to me?**

R: That doesn’t help, the only way you can tell me sorry is maybe if it’s a financial problem then you tell me sorry and give me the cash to deal with my current financial constraint that would be a welcome idea. That is why I prefer dealing with the accountant because if I tell him I don’t have money he can give me even a hundred shillings or if I have been sent back home to collect fees and I don’t have money then he can give me bus fare to come back home. But there are others that I decide to deal with on my own.

**M: (Name) whom do you talk to?**

R: It depends on the different situations but I can talk to my dad and then to my best friend and my mum.

**M: When you have issues to do with HIV whom do you talk to?**

R: I can talk to my best friend we are very close.

**M: (Name) when you have issues to do with HIV whom do you talk to?**

R: I don’t talk to anyone i would rather come to the facility and speak to the counselor as she is the expert, I feel more comfortable here because she is more informed. I cannot share with my mum because if I tell her that I forgot to take medication then she will be so angry at me and even make me bad so I would rather come to someone neutral. If I tell my mum she will start telling me that the life I am putting at risk is mine and if I joke around I will die and so forth, so I keep that away from her and I would rather tell the counselor.

**M: You just hold on to your issue until you come and speak to the counselor?**

R: Yes, that is where I feel comfortable. When going to school my mum counts and packs my medication that I will take for the time I will be in school, for instance if I will be in school for forty days she will pack fifty for me with just a few extras if I come back with thirty then it means I have not been taking my medication and when she sees that she really gets upset.

**M: (Name) whom do you talk to?**

R: I don’t speak to anyone.

**M: What if you have any challenges like when you fail to take your medicine whom do you talk to?,**

R: I don’t speak to no one that is my problem to deal with so I don’t think I should be talking to anyone about that and I deal with it.

**M: How do you deal with it?**

R: Even if I talk to that person they don’t help me in any way, so it doesn’t add up me putting up my business to people who don’t help me at all. This life that I am protecting is mine and if anything happens, it happens to me, we will not go together six feet under.

**M: Do you feel like you receive support from home?**

R: Yes, I do.

**M: Anyone else?**

R: It is there all the time.

**M: (Name) when do you feel like you get support?**

R: I feel like I get support when it comes to taking my medicine because I have family members who always remind me to take my medication, but on the other hand if I forget to take medicine and I tell my mum then she blames me and gets mad at me accusing me of shortening my life and wanting to die and that really makes me sad.

**M: At times you forget taking your medicine?**

R: Yes, I do especially during funkies and when I go home for the holidays and I have many activities.

**M: Do you have funkies many times while in school?**

R: Yes, they are very many, especially towards the end of first term and then second term.

**M: Okay.**

R: Especially in form two when things start to become really tough because you are just getting into the system and then you have the funkies as well. so at times during the funkies we get ladies coming to our school and we become over excited the whole night and in the morning you forget your medication you are just thinking of the ladies. So basically while in school at times we have funkies all weekends during the first term its sports and second term its clubs but third term its more chilled so you can imagine the number of times you can forget taking your medication if you don’t focus. I was using Septrin the second month but I would end up going back home with very many as a result of attending more funkies and forgetting to take the medicine but normally during third term I take my medication properly. So, at times you come from the funkies and you decide to relax on the bed a little because I take mine at 10:00, 11:00p.m but you end up sleeping till morning. But if I wake up at 4:00a.m then I can still take my medicine.

**M: So we said that most of the time we get support from the family?**

R: Yes.

**M: What kind of support do we get from them?**

R: Financial.

**M: Any other support?**

R: Basic needs like financial and emotional support by reminding you to take medicine and that love and care.

**M: Any other support?**

R: Financial as well as emotional when I fail to take medicine and then I have to confide in my dad because my mum really discourages me especially when it comes to not taking medicine she doesn’t mince her words and easily reminds me that if I fail to take medicine I risk dying.

**M: How about emotional support do you get that support?**

R: In my case my father leaves very early and comes back very late so we get very little time or no time at all unless I stay late and then my mother is a very busy woman and she only remembers to ask me if I took medicine the previous night in the morning and at times I don’t remember if I did take the medicine or not, so I just lie to her that I took the medicine while I didn’t.

**M: (Name) any other support?**

R: It is hard to confide in them because at times when I get sick then she will start blaming me for not taking my medicine and at times I don’t feel the need of talking to them.

**M: So even if you develop a cold she blames you?**

R: Yes, she does.

**M: If you develop a headache it’s your fault?**

R: Yes, like the other day I really got ill and it was such a big issue. I had travelled to Mombasa but I came back with so many pills and when my mum counted them and saw the ones that were left so really go angry with me. And then when I came back before adapting again I became sick then I was being blamed that it was because I had not taken all the medication that I was supposed to take.

**M: Are there any people you wish you would be open with but for some reason you don’t feel open to them?**

R: The cook, I know that in school once you talk to the cook then if they understand you, your meals are guaranteed to improve but the challenge might be if they fail to keep your secret and then everyone knows.

**M: How about you?**

R: There is no one.

**M: How about you is there anyone you wish they knew your status?**

R: There is no one.

**M: Have you ever experienced HIV stigma?**

R: No, I haven’t.

**M: Howa bout you (name)?**

R: No.

**M: (Name)**

R: No.

**M: We have talked about taking our medicine and it also came out that there are times when you skip medicine for one reason or the other but are there any other occasions or at** **what other point do you face any difficulties taking the ARV’s? What is the most challenging thing about it?**

R: The size, it should be reduced.

**M: What should be done to make it easier for you to continue taking your drugs?**

R: It should be a liquid and not a tablet

**M: Any other recommendations?**

R: While taking Septrin you need to drink a lot of water and I am not a good water drinker so that is a big challenge to me, if it was turned to a syrup that would increase my uptake.

**M: Any other person?**

R: At times in school I just feel lazy, at times I am busy chatting with friends in class, then we move to the dormitory and by the time we finish chatting I get lazy I don’t feel like going for the water and it’s time to take medicine and I just feel lazy. I cannot send anyone to get me the water then they start getting all nosy in my business so that is another challenge.

**M: How about you (name)?**

R: It is the same reason, at times I just feel lazy and then some people naturally fear taking medication.

**M: But you are forced to take them?**

R: Yes,

**M: Okay.**

R: And you cannot drink that medicine without water, and the pill is very bitter.

**M: What encourages you or motivates you to continue taking the medicine?**

R: That is my life and I have every right to protect it, I am taking it and hoping that one point I will get to a point where it will be undetectable

**M: Is that pressure or motivation?**

R: Motivation.

**M: What else motivates you to continue taking your medicine?**

R: I want it to be undetectable, and then my life depends on it and if I fail to take it then I will become ill and that can even result to my demise.

**M: Are there times you don’t feel like taking medicine?**

R: No.

**M: Are you always happy to take medicine all the time?**

R: You don’t feel happy but you take it because you need to stay alive

**M: What occasions don’t you feel like taking medicine?**

R: When I get to bed and I start feeling lazy, and then I remember that I have to take medicine, that moment really makes me feel so bad because I really get so lazy.

**M: Any other occasions don’t you feel like taking medicine?**

R: At times you are busy chatting with friends and the stories are really hot and you remember that it’s time for you to take medicine and you cannot just take off abruptly, so that is also another challenge.

**M: For those who have family members who are taking medicine, in your opinion what encourages them to continue taking medicine?**

R: It’s the same thing to extend your life and to reduce the viral load.

**M: How about you, you said your mum is your motivator so what encourages her?**

R: The fact that she wants to be alive and also to be a role model for me.

**M: Okay.**

R: I am the motivator in my family so everyone looks up to me.

**M: How about you?**

R: My mum she takes drugs on time so that she can at least encourage me to also take mine on time

**M: (Name) do you know anyone in your family who is currently taking ARV;s?**

R: No, I am the only one.

**M: How about you?**

R: My mother but I am the one who reminds her to take her medicine. And then my dad at times we are sitting together and we are both supposed to take medicine but we just ignore and we take later. At times our mum comes to reminds us and then at times you just sit down and remember that there is something you have not done and then you remember you have not taken the medicine and you go for it.

**M: Anyone else?**

R: At times my mum is right at home just going about her duties, maybe she is on her phone and she might forget to take her medication so its upon me to remind her because if I fail to do that then she will blame me again.

**M: So being reminded really works and it helps?**

R: Yes.

**M: (Name)**

R: I would say my dad, he is very tough and has accepted his status so he drinks loyally.

**M: Anyone else?**

R: I have an alarm.

**M: And your sister or did she stop?**

R: She is still taking but we don’t spend all the time together, everyone goes about their business,

**M: And then we are all growing up and then those who are in youth clinics will soon join the adult’s clinic, so how do you feel about joining the adult’s clinic when the time comes in the future when you turn 18 years?**

R: It is okay as long as my medicine is not changed.

**M: You don’t want your drugs to be changed?**

R: Yes.

**M: But how do you feel about joining the adult’s clinic?**

R: I don’t mind, I am already used to the current medicine and the time if that is changed then that will affect me drastically.

**M: Why not?**

R: I don’t have a specific time that I take medicine and I take it anytime between 8:00 to 1:00p.m so if that routine is changed then it might affect my schedule

**M: (Name) how do you feel about joining the adult’s clinic**

R: I don’t mind, there is no big change other than joining the adults and I don’t mind being around them because they are more experienced and I can learn a lot from them.

**M: You do not have any concerns?**

R: No, it’s just life we all have to move from one step to the other.

**M: Any other concerns about joining the adult’s clinic**

R: I don’t have any concerns, when I am asked to move, I will move.

**M: How about leaving behind the friends you have made in the youth clinic?**

R: It will be hard but that is life. It is just like moving from class 8 to form 1, I left behind people that I knew and I moved ahead and made more friends and I adapted it is all about life.

**M: Any concerns about joining the adult’s clinic, what challenges do you foresee?**

R: I don’t foresee any challenges.

**M: (Name) you have moved to the adult’s clinic?**

R: Yes.

**M: How has your experience there been?**

R: It is good, because at times you sit with those adults and they share information that you didn’t even know, maybe if you were sitting next to them they teach you a lot so I am very grateful.

**M: Are there issues you feel that if you were given more information about, then you would learn more about your health and become more healthy?**

R: Yes, about our future, I would definitely like to get married and have a family in the future but is my baby going to be HIV positive or negative, and what should be done so that I don’t end up having a baby that is HIV positive?

**M: You would like to get such information?**

R: Yes.

**M: Anyone else?**

R: I was told by my mum that there is more information nowadays and nowadays children born will not be infected and they will be born HIV negative and can live negatively as long as you do what you are instructed to do by the doctors.

**M: Any other issues?**

R: I am just wondering when this cure will come.

**M: That is what you would like to know?**

R: Yes.

**M: Any other concerns?**

R: If there is lack of medicine, what can be the other alternative so that you continue living a healthy life? At times we go camping and we might not have medicine around so that is the other option? I was given a pill box but at times I get tired of carrying it. While at school I put it in the bag and then at times I forget the bag in the dormitory so is there any other thing we can be given or we can do if you fail to take your medicine over a specific period of time?

**M: Are there areas about your health that you wish to discuss when you get to the clinic?**

R: There is none.

**M: We are just winding up but where do you see yourself in 5 years from now?**

R: My viral load would have reduced and I will continue taking medicine and I will be living a healthy life.

**M: Any other person?**

R: I visualize a healthy person, probably not taking medicine anymore because a cure will have been found miraculously and I will be healthy.

**M: Okay.**

R: My viral load will have drastically reduced.

**M: Where do you see yourself in 5 years from now?**

R: I will be a successful person and I will have met someone accepting me and maybe someone in the same situation as me and we will be trying to build a life together.

**M: And still thinking of those five years do you feel that there are challenges you might face in the future?**

R: Yes.

**M: What challenges will you face?**

R: I will clear school next year and maybe I will start dating and I might meet a very good woman that I like and maybe I want us to get serious in the future and now when it gets to the point of disclosing my status that would really be hard for me. I am not sure if she will stay once she hears my status and I wouldn’t want to marry her without telling her the truth that will definitely strain the relationship.

**M: Anyone else?**

R: It is the same issue have to disclose my status to my significant other and then I am not sure if she will disclose it to other people or if she will keep quiet.

**M: Any other challenges?**

R: Disclosing my status to my partner.

**M: (Name) thinking of the next 5 years and your aspirations, what are the challenges you might face?**

R: I have tried very hard to keep this secret and maybe when I join campus I might get clumsy with my status and medication and then someone finds out and then I don’t know how they will react.

**M: Do you currently have girlfriends?**

R: I do.

**M: The rest of you?**

R: We do.

**M: But you have not told them your status?**

R: It is very hard and even you find our parents telling us that is just a stage and there is a high chance that is not the person that we are going to get married to, so there is no need of disclosing my status as long as we are not getting intimate

**M: You don’t feel like you might disclose your status to her?**

R: We might break up and she will tell everyone, and so I have to hide that for now.

**M: How do you handle your sexuality?**

R: I just abstain.

**M: Are you sure?**

R: Yes.

**M: Your girlfriends are not giving you pressure?**

R: No, other than pressure my concern is if she gets pregnant then what happens to that baby, we need a lot of information first before bringing a baby to this world.

**M: They don’t give you pressure, it might not be verbal but maybe the way she is dressed in a mini skirt you can see the cleavage nothing happens?**

R: Then I can use a condom, because even during the training sessions one of the trainers told us that he know that he cannot even ask us to abstain because we will not listen, but he advised us to make sure we use a condom. So if there is too much pressure then I use a condom and if I don’t feel like then she has to go because I don’t want to risk her life and even mine further. I remember that when I came in November I talked to a certain staff I cannot remember his name but he advised me to take some condom because the long holidays were coming and I needed to protect myself. But the challenge is I cannot take those condoms home so I didn’t take them.

**M: That is the question I wanted to ask you where do you keep those condoms then?**

R: I sell them and make some money.

**M: What if you need condoms and you don’t have money to buy condoms what do you do?**

R: If I don’t have money and no condom and the lady also doesn’t have condoms and she has no money then the party stops there, nothing can take place without condoms.

**M: Even the ladies don’t mind buying condoms?**

R: They buy.

**M: Do some come with condoms?**

R: Yes, if they ask you to come then they must have their own protection.

**M: What if they ask you to have intercourse without condoms?**

R: That is not even an option.

**M: Name, would you allow it?**

R: No, we cannot have intercourse without a condom.

**M: The challenge is when you think of marriage and then the fact that you have to disclose?**

R: Yes, that is when the real issue will come, right now I cannot tell a girlfriend that I am positive, yet I have not cleared school. And then I have to go to university and then some years after university as a I settle, I don’t think if you tell a lady now she can hold on to that secret for long she will leave you. So I am holding that secret for that lady that I will have dated for long and we are in a serious relationship and then when we start talking marriage then I will tell her.

**M: Okay.**

R: There are very slim chances of a positive person getting married to a negative person. I don’t even know where my mum and dad met I feel that probably the virus was passed down from either one of their parents who are now my grandparents so it’s a long trail that goes backwards.

**M: So you feel that its inherited?**

R: It could be.

**M: Okay.**

R: It is stressful but when it comes to that point of disclosing my status then we have to because at that point we will be discussing about our house, our future and getting children those are sensitive and serious issues that the moment of truth has to come. At the moment we might have a little fun but with precaution but we are well aware that a time will come in the future that we have to speak the truth. You might be serious about a certain lady and maybe you are not the only one that she is dating.

**M: Is that possible?**

R: Yes, especially in school, you might think you are the only one and maybe she has other boyfriends.

**M: Okay.**

R: And there is that belief that a girl is only your girlfriend when you are together but when she goes out nobody knows what she is doing and whom she is talking to. Like even during those functions one time she might be with yours, another time you find her with a boy from another school, not even your school so you cannot be certain you are the only one because you are never sure how many others she dates long the way.

**M: How about you?**

R: I believe that yes it might be hard disclosing your status but if your partner really loves you then he will accept you the way you are and then you can find a solution together.

people.

**M: Thank you, you were open and I enjoyed the discussion, it was interesting to me, I hope it was interesting to you.**

R: A lot.
