# Supplementary material for: Psychosocial needs among older perinatally infected adolescents living with HIV and transitioning to adult care in Kenya
Source: PLoS One. 2020 Jul 29;15(7):e0233451. doi: 10.1371/journal.pone.0233451 (PMC7390380; doi:10.1371/journal.pone.0233451)
Supplement: S1 File — (ZIP) [file pone.0233451.s002.zip › uploaded final PLOS/Reviewed Transcripts/FGD3.doc]

**M:	Okay so my first question is about how we coped emotionally with the knowledge of your status, your HIV status, how did you cope with that emotionally?**

R:	I knew about my status in class 6 while I had attended a counseling session with my mum and in that process I was asked if I knew the reason why I was taking the medication and I responded yes and then they asked if I knew my status and I said I didn’t know and then I was told that I was HIV positive. At first I cried but my mum calmed me down and told me to stop crying because it was not my fault that I had contracted HIV but before I completely accepted my question I went through a lot of challenges and I kept asking myself many questions and I kept asking God why he allowed it to happen to me. And I remember at some point I had completely isolated myself and I felt like the odd one out so those were the challenges I faced, I used to be lonely and I wanted to spend time alone in isolation. So, initially I was really hurt but I came to realize that I was not the only one and then I learnt from this group that I have been attending and I listened to the information that was shared during those sessions and I realized that I have nothing to worry about and I can still live a healthy life regardless of my status.

**M:	How long did it take you to reach that point of acceptance from the moment you knew about your status?**

R:	It took long because in 2014 while I was in class 6 I was feeling very stressed and even while in class 7 though it had started to reduce and then in class 8 it reduced so it has taken me two years to accept my status.

**M:	Thanks for sharing, the rest of us, when did you know about your status and how did you cope with that emotionally?**

R:	I confirmed my status when I was in form one but while in class 8 my mum had sensed and she never told me. I remember being very sick when I was sitting for my KCPE exams but I received treatment and recovered. Then I joined form one and became sick and I was taken to several hospitals but finally I was brought tohospital and I was tested and they realized that I was suffering from HIV. I remember my initial reaction was I will not take the medication, am even prepared to die and I stayed for 8 months before I started taking the medication. I received counseling and I remember suffering from TB and after a month I started taking the medication.

**M:	When you started taking this medication had you already been placed under the TB treatment program?**

R:	No.

**M:	What brought about the change of heart?**

R:	My counselor spoke to me and she made me realize that I was not the only one suffering from HIV and then in our family it is only my dad and myself who were HIV positive so that thing was really giving me challenges and made me think a lot.

**M:	Anyone else, when did you know about your status and how did you cope with that emotionally?**

R:	I knew about it when I was 12 years, we had travelled abroad with my parents and I remember at first I was shocked and it took me one year to overcome. I was initially very stressed, but prior to that I used to wonder why I had to take all that medication yet I didn’t feel sick but when I was told I continued taking the medication but it was hard for me.

**M:	So by the time they disclosed to you, you were already under medication?**

R:	Yes.

**M:	You used to take medication but you didn’t understand why?**

R:	Yes, I didn’t understand why.

**M:	How did you react?**

R:	I was just shocked and I got scared and it took time to overcome.

**M:	Thanks for sharing, anyone else?**

R:	I was in class 5 and I started taking medicine when I was in class 5 but I didn’t know I was HIV positive and so I remember asking my dad why I had to continually take the medication and he told me it was for my chest and so I believed him and continued with the medication.

**M:	Okay.**

R:	Then I remember later on in my life I went to the doctor to get the medication but the doctor asked me if I knew why I was taking the medication and I told her it was for my chest and she asked me who had told me that I had a chest problem and I told her it was my dad. And then she counseled me.

**M:	Had you come alone?**

R:	I was with my dad and then she finally disclosed my status to me and that moment I really felt lonely, and I cried a lot and then when I got home I faced many challenges. I didn’t want to be with my friends because I felt that they might realize that something is wrong. I felt that they might not want to talk to me if they knew my status. And I remember at some point I didn’t want to take the medication but after attending many counseling sessions I realized that I was not alone and I need to be positive and live my life.

**M:	What else made you realize that you were not alone?**

R:	The hospital visits, when I come I see other patients and that made me realize that I was not alone.

**M:	The rest of us, when did you know about your status and how did you cope with that emotionally?**

R:	I think I have taken the medication for the longest period; I started taking the medication when i was in class 3 that was back in 2007. I remember during my hospital visit I had mumps and then it came to a point where my CD4 was at 200 because of that infection and according to the doctor, they told us that the CD4 should be 1200 and above and the irony was I was taking the medication thinking that I was treating the mumps. Then when I was in class 6 I remember the Doctor asking me if I knew why I was taking the medication and I told him it was for the mumps and I was very shocked. I kept asking him if he was sure and he asked me if I needed us to redo the test and I told him no. then after that he took me for counseling and I met a counselor who spoke to me and I remember being introduced to a certain youth who was also HIV positive, but he passed on because he was in denial and he refused to adhere and take the medication. I remember he was the same person who told me to adhere to the medication while he was not taking his but unfortunately he passed on.

**M:	He was encouraging you?**

R:	Yes, but he stopped at some point and he became sick, his skin was peeling, then he was infected with meningitis and he died. So, after seeing his health change for the worse I took it up on myself to ensure that I adhere to the medication. So that was like my strong turning point and made me realize that I need to take my medication seriously.

**M:	Anyone else?**

R:	I remember taking the medication when I was very young but I never understood why then one day when I was in class 3 my sister asked me if I knew why I was taking the medicine and I told her mum told me to take them, they are multivitamins.

**M:	Was that your elder sister?**

R:	Yes, then when I got class four both my mum and my sister opened up to me about my status and I didn’t mind taking the medication it had already become part of my routine and I never took it really hard on me but I remember the time that I took it really hard was when I was in class 6 and realized that it was only me and my mum who were taking the medication out of five children it was only me who was affected and it really made me feel really bad. So I came to hospital and saw a counselor and she spoke to me and now I have accepted my condition and am now moving on with my life.

**M:	The rest of us, when did you know about your status and how did you cope with that emotionally?**

R:	I remember taking medication but whenever I used to ask my mum she would never tell me the truth, she kept ignoring me and then one time I got very sick and went to the hospital and when they run several tests I was confirmed to be HIV positive but I was not told, it was my mum who was told and when I continued taking medication.

**M:	So when did you know about your status and how old were you?**

R:	I was 11 years in class 5 that is when I was told but I didn’t feel as if it was something serious and then one time I became very sick and we came to hospital and I was give more medication and thereafter my mum and another doctor opened up to me and after being told by two people I realized this was a serious issue and I have to take my medication seriously since it was my life and nobody else was taking the medication at home. But after sometime I stopped worrying and adjusted but initially I kept wondering why I was the only one taking medication at home.

**M:	Okay finally.**

R:	I knew about my status in form one first term during the mid-term I fell ill and had sores on my back and all over my body. I was taken to Thika medical and I was told my blood was unclear and I was given some medicine but that didn’t help. So I was referred to Hospital and one doctor looked at me and told me there was no need of being tested as he could already see what I was suffering from. So we went to a certain room and a blood test was done and I turned out HIV positive while my sister was negative. So, I remember asking her what that meant and she told me to ask the doctor.

**M:	Is she your elder sister?**

R:	Yes, we had come with her and so I went to the doctor and she talked to me, she told me even if I am HIV positive it is not the end of the world if I adhere to the medication I can still live a long and healthy life and do anything I want in life. Then I came to the doctor’s office and I opened a file and I was tested and I was given medication and upon the blood test being done my CD4 was at 31 and I was told it was too low and so I was given medication and continued with my medication. Then one time while I was in form two, while I was taking out my medication, a friend saw me and asked me what medication I take daily and I told her they were for my chest pain. And then another time she got suspicious and I opened up to her and she was very disrespectful because she went around the whole school telling everyone that I was HIV positive and I was very traumatized and I stopped taking medication for 6 months and my condition became worse. I was taken back to hospital and they discovered that I was not taking my medication. I was taken for counseling and even my sister had to intervene and talk to me. So, she eventually told me that mum died due to this disease and I was wondering how come she died if she had been taking the medication? And she told me there was a reason why mum died it was because she never used to take her medication. At that point I didn’t even care and I wanted to die just like my mum and when she realized that I had become too stubborn she opened up to me again and took me to see one cousins who was also infected and his condition was worse at that point and he didn’t take medication the way he should be and so by that time he had been taken to several hospitals and he had started losing his eye sight. At that point he had basically given up but he advised me to take my medication seriously and after seeing him in that state I decided to take my medication seriously, but sadly he passed on and was buried at Lang’ata.

**M:	And you decided to adhere to medication?**

R:	Yes.

**M:	Why do you think many teenagers don’t like taking their HIV medication?**

R:	They find it difficult taking medication daily.

R:	And also having to keep that time.

**M:	Keeping time is difficult?**

R:	Yes, and even when you need to travel at times you cancel your plans because you might be away at that time and you need to be taking your medication at that time.

**M:	Any other reasons?**

R:	And you cannot forget taking that medication.

**M:	Anything else?**

R:	Some people talking negatively about this medication and it makes me believe what they are saying is true.

**M:	So people’s perception and then you don’t want to be associated with the medication?**

R:	Yes.

**M:	Anything else?**

R:	The medication is very bitter.

R:	And the tablets are very huge.

R:	I have to accompany it with porridge.

R:	And it has a lingering after taste.

R:	Even tea, due to the after taste.

**M:	Anything else?**

R:	And it has a bad smell.

**M:	What do you take the medication with?**

R:	Water.

**M:	Any other reasons?**

R:	I don’t know what used to happen but at the beginning I would take that medication but after about 30 minutes I would vomit.

**M:	After 30 minutes?**

R:	Yes, I would vomit or feel nauseated, and then I would lack appetite.

**M:	What happens when you vomit it out?**

R:	You cannot take more medication because it will be past your time for taking the medication so you have to wait until the following day or the following time depending on our routine.

R:	If you take more it will be an overdose and you know that you don’t vomit it all out.

**M:	So taking that medication is a challenge?**

R:	Yes, it’s a big challenge.

**M:	Anything else?**

R:	At times you are sitting with some people whom you are not comfortable with and you realize you only have 5 minutes to take your medication and it becomes tricky to avoid them seeing you taking the medication.

**M:	Okay.**

R:	I would have taken them but at times people are curious and ask too many questions and then at times I don’t feel like responding to those questions. That really irritates me.

**M:	What questions do they ask?**

R:	They will ask why you are taking that medication, for what diseases.

**M:	Anything else?**

R:	And then they vary with size so they will ask you why your pills are so big in size.

**M:	Who are these people who ask you such questions? And is it because they catch you off guard or you are just uncomfortable taking it?**

R:	Family members.

R:	Relative

R:	Friends.

R:	Cousins.

R:	Cousins

R:	School friends.

R:	For instance, if you are in boarding school and your time comes you have to take the medication you could be in class and you will still be forced to take the medication, and of course while taking them some people are curious and they will ask you why you are taking the medication.

**M:	Are there occasions you just take the drugs willingly without worrying who is watching you?**

R:	There is no time anyone can just take the medicine without worrying if someone is watching them we are always conscious of our surroundings.

**M:	Why?**

R:	You just feel bad if someone finds you taking medication because you have to explain to them things that are personal to you. And if you tell one person like she said they will go and tell everyone and you feel like your privacy has been violated. And then you will start feeling bad so most of the time we hide when taking the medication.

**M:	Anyone else?**

R:	Then when you tell some people they will start asking why those tablets are really big or some start looking at you like you are very sick and all that and if you tell them, they will not trust you.

**M:	Okay.**

R:	In school I cannot take when everyone is watching me, because people in school are always spreading rumors and at that point maybe you are in four form and you find a form one just coming to ask for you to see who you are and that really made me uncomfortable.

**M:	Do you feel that in school there are other infected students who are taking ARV’s or do you feel you are the only one on HIV medication?**

R:	I am sure there are other students.

**M:	But you cannot confide in them and become friends?**

R:	Everyone keeps to themselves.

R:	Or you approach them and they confide in you, you will look like you had already been told you were just coming to confirm if it’s true and that can make the other person really uncomfortable.

**M:	Are there days you feel very discouraged?**

R:	Yes.

**M:	Which day are these and where do you receive support and from whom?**

R:	I would say my mum, is the worst support system and after listening to her talk badly about my medication I always ensure that I take my medication on time.

**M:	What does she say?**

R:	She says they are so big at times she throws them outside and I have to go and pick them and take them, it really makes me feel bad.

**M:	She discourages you?**

R:	Yes, she does.

**M:	So instead of encouraging you she discourages you?**

R:	Yes, so at times when she begins to talk that way I just go outside to forget about it or I can just go away and keep to myself and start singing.

**M:	When you feel discouraged how you do cope and who supports you?**

R:	My entire family know about my status, besides my younger cousins whom I have not told because they are very young but when I feel discouraged I speak to my family members and they are very supportive and encourage me to take medication to avoid the opportunistic diseases since these medications keep me alive. And who knows maybe one time a cure might be found so my family has been very supportive. And then when I come to Hospital, the health providers speak to me nicely and the counselors also support me.

**M:	How about you how do you cope when you feel discouraged and who supports you?**

R:	You can motivate yourself by sleeping.

**M:	Any other person?**

R:	I also sleep when am feeling down.

**M:	Does it help you?**

R:	Yes, it does.

**M:	Anyone else? How do you cope?**

R:	My family, and then at times we go for walks and joke around and that helps me forget.

**M:	Okay.**

R:	We are five boys, five girls that is my crew in my neighborhood so when I am feeling discouraged we meet up and joke around, play games, have fun and that helps me to forget.

**M:	What do you do when you feel discouraged or how do you cope and who supports you?**

R:	I listen to music.

R:	Skating.

R:	Listening to rhumba music.

**M:	I know it was emotional when you think about how you found out your status but thinking about the process and how it was revealed to you, is there anything you would like to be done differently? Would you have wanted the disclosure to have been done differently?**

R:	I think when we come for our results they should not make the counseling session too long it tortures me more, they should just make it short and to the point. Like she was told it was chest problems while it was not, its better you tell me straight up then I figure out how to cope with it.

**M:	Okay.**

R:	So that if you need to sleep over it or cry then you can do that and let the healing begin early.

**M:	Okay.**

R:	I don’t like how they do it here in Hospital because there is that tone the doctor uses and it makes you start doubting yourself, its better if they didn’t prolong that session and spare me the guessing.

**M:	So that suspense makes you feel that there is something they are trying to tell you?**

R:	Yes, when they do that something just comes to your mind.

**M:	So remember the last time you had that conversation what thoughts came to your head?**

R:	I was told directly by a friend.

**M:	Is there any information you would like to be given that day when they were giving you the results but they didn’t?**

R:	If they told me early on about my status it would not have taken me that long to accept my situation. I was told when I was in class 6 and I took up to 2 years to accept myself, and I started feeling that my mother didn’t love me because if she did then she would have told me earlier on. If they withhold such information it can have severe consequences for instance for someone who had high blood pressure they might just break that news to you and you collapse.

**M:	Any other emotions you felt at that point?**

R:	I felt really bad I wanted to strangle my mum for withholding such information about my life from me. I remember that day after being told of my status on our way home we didn’t talk. I didn’t want to talk to her and I remember she had bought some fries for us to eat but she had to eat them alone because I didn’t want to eat that day.

**M:	How long did you give that that cold war?**

R:	It took time before I spoke to her.

**M:	Did she try reaching out to you?**

R:	She tried reaching out to me but I was not willing to forgive her, I was feeling really bad, I could not imagine why she took long to forgive me. I would have wished that she told me earlier so that I would have grown up knowing my condition. There was a time I lapsed in my medication and I became very ill I had to be admitted here at Hospital.

**M:	You just refused to take the medication?**

R:	Yes, I did. I normally have a bad temper so she made it worse, she was forcing me to take the medication and I would spit it out. And since that day, everyone at home knows my tempera and they don’t hold back information from me, if am sick or whatever they have to inform me immediately.

**M:	Okay.**

R:	I remember lashing out at the female doctor for keeping that secret from me because I used to come here but she never told me until it was too late so that day she saw my bad side; I was not laughing with anyone.

**M:	Were you told about adherence after they disclosed your status?**

R:	I was not told.

**M:	How about you?**

R:	I was told.

**M:	What were you told?**

R:	I was told that I have to adhere to my medication and I took it seriously after seeing that guy I was talking about called XX who didn’t adhere to his medication and he ended up dying. So the doctor told me that I should adhere to my medication if I don’t want to end up like XX, that really scared me and I decided to continually take my medication.

**M:	Okay.**

R:	I remember her telling me that I have to take the medication daily and I have to keep time and I was wondering why I have to keep time. But she told me for this specific medication I have to keep time for it to be effective.

**M:	Were you told about adherence on the day they disclosed your status to you?**

R:	Yes. I remember when I started I used to take the medication every morning and evening and my mum used to constantly remind me to take them on time, initially I didn’t understand why but with time I got used to it and I always take them on time.

**M:	Do you think such information should have been shared to you on the same day you were told about your status?**

R:	Most people are normally told on the same day.

R:	Not all it depends on the doctors you find on duty.

**M:	Anyone else?**

R:	I was not told the same day; I was told later on.

**M:	Were you told immediately?**

R:	Yes, I was told everything immediately.

**M:	Apart from your caregivers, like parents have you disclosed your status to anyone?**

R:	Yes, to my friend but she told everyone about it, and I didn’t feel good about it, but these days I don’t mind.

**M:	How did it affect you that time she told everyone or what was their reaction?**

R:	People didn’t want to speak to me, they felt like I would spread the diseases to them. I felt lonely and after all that I mastered up my courage and decided to continue taking my medication especially after my sister told me that my mother died due to non-adherence to medication, I told myself that I will not feel ashamed and die because of anyone.

**M:	So you just pondered over it and decided to continue with your** medication?

R:	Yes.

**M:	Anyone else have you disclosed your status to anyone?**

R:	Only my mom knows.

**M:	Anyone else?**

R:	Only my mom knows.

**M:	Your family members don’t know?**

R:	No, they don’t.

**M:	And you told a friend?**

R:	Yes.

**M:	Is there someone that you normally look at and wish you would disclose your status to them?**

R:	My boyfriend, at times I wish to tell him but something keeps stopping me. So, I always wish that I could tell him so that he is aware of my status and he could decide what he wants if wants to continue with this relationship because some of these boyfriends end up being our husbands so we would like to be honest from the beginning of our relationship.

**M:	Okay.**

R:	It depends on the individuals and how much you trust them, there are some friends you might think you trust but they go behind your back and tell to everyone. So you always need to have that one confidant that you can tell everything.

**M:	Anyone else you wish you would disclose your status to them?**

R:	Some relatives.

**M:	Why?**

R:	For instance, my aunt, the sister to my late mum she treats me like her own child and she really loves me and I love her and I always wish she knew.

**M:	What’s stopping you from telling her?**

R:	My step mum treats me so bad, and it makes me feel so bad to the extent that I always end up just isolating myself by sleeping.

**M:	So does she discourage you from telling your aunt?**

R:	Yes,

**M:	Anyone else?**

R:	If you are close to your class teacher at times you want to disclose to them.

**M:	Okay.**

R:	I cannot tell my class teacher.

**M:	Okay it’s not a debate let us listen to her.**

R:	I would want to tell my class teacher because we have some very good class teachers and other are not good because you never know them that well and you might tell them they inform everyone in school but at times they are so good to us in school and you feel like you can confide in them.

**M:	Anyone else?**

R:	I don’t feel like I can trust my class teacher because I have a feeling that when they go back to the classroom my story will be the topic of discussion so I can never trust them fully.

**M:	Do you feel that they talk about students in the classroom?**

R:	Yes, they do.

**M:	Have you ever found them?**

R:	At times they go to the staff room to chat and the stories might become too sweet and they might spill the beans.

R:	But this issue of class teachers since they are always changing does it mean you will be telling all your class teachers?

R:	Not really but the one that I feel comfortable in.

**M:	Okay so I can see there is a challenge in term of disclosing your status, so if your family members or close friends knew about your status what challenges would that pose?**

R:	Some will take it positive but others will start asking me unnecessary questions that am not prepared to answer.

**M:	Okay.**

R:	Family members vary, some are good and some are bad.

**M:	How will they act?**

R:	Some will treat you differently, they will not be the same.

**M:	How will they treat you?**

R:	For instance, if your aunty liked you before if you tell her she might not accept you, and she might tell her children not to be close to you or to interact with you in any way.

**M:	Let me give you an example when we started we talked about how we felt when we knew about our status and some people felt bad, they isolated themselves and even didn’t want to adhere to medication but eventually you learnt that you are not alone and there are some people you can talk to so in light of that don’t you think that they might still support you better than if they didn’t know?**

R:	I think it’s the other way round they will treat you better when they know less about you than when they know more about you. They might not do it knowingly but there is part of them that will not treat you the same once they know about your status.

**M:	What we are talking about is our perception but we are not sure they will treat us that way.**

R:	And that is why we said it depends and we are not able to know at this point, maybe someone who used to treat you badly will turn around and treat you better and the person who used to treat you well will treat you badly so it varies.

**M:	So you would rather not risk because you don’t know how they will react?**

R:	Yes, we are safer that way.

**M:	Would you have wished to be given more information when they disclosed to you about your sexual and reproductive health?**

R:	Yes.

**M:	How about contraception?**

R:	If it was disclosed to us at class 2 then you know there is no way they can tell you about contraception at that point in your life. If the disclosure is done in high school maybe at form 1 then I can be told about contraception.

**M:	But would you like to receive that information about contraception?**

R:	I think at this age there is nothing more they can tell us, I think they have disclosed everything to us.

**M:	What have you been told; I would like to have a feel of the topic you have been told?**

R:	We have been told about two options either abstain or use a condom, we were even told about that today.

R:	That is something they tell us all the time.

**M:	Either abstain or use a condom?**

R:	Yes, those are the only two options we have been given.

**M:	Is there any information you would like to receive?**

R:	At times they come to our head but when you don’t think about it for some time you forget.

**M:	Give me examples.**

R:	Today happens to the day that I cannot any of them.

R:	At times you remember those questions then you sort of answer them on your own.

**M:	So you don’t always need to go to the health providers for answers?**

R:	No, at times we think on our own.

**M:	Where do those answers come from?**

R:	From people talking.

R:	And other people’s reactions.

R:	And their experiences.

**M:	Where else?**

R:	Movies.

**M:	So you sit and wait until you watch a movie?**

R:	For those who watch soaps we get some responses from the movies.

**M:	Do you get information about marriage and having kids, is that information you would like to receive?**

R:	That is vision 2030.

**M:	But you told me you have a boyfriend?**

R:	Yes, but we are not thinking about kids and family at the moment.

**M:	Anyone else?**

R:	I was told that if I have a boyfriend and he is negative and am positive I should come so that they run tests so that we can be counseled on the things we need to be aware of as we prepare to have children.

**M:	So if you want to have kids then you have to go for the tests?**

R:	Yes.

**M:	How about the rest of you, would you like to get information about marriage and having kids?**

R:	Not yet.

R:	We are still in school.

**M:	So, regardless of that, would you still like to be told about marriage and having kids**

R:	No, maybe more on abstinence or use of contraceptives.

**M:	Anyone else?**

R:	I think it would be better to be told earlier so that we can be informed instead of being uniformed then you end up pregnant and out of school.

R:	I disagree I feel that the more you are told about something the more curious you will be.

**M:	Okay.**

R:	I think we need that information so that you are well aware that as I go to meet this boyfriend and there is a likelihood of us engaging in sex, I need to be aware that this could happen and I might end up pregnant and dropping out of school.

**M:	So we should share that information?**

R:	Yes, and let people decide what to do with that information rather than not being informed.

**M:	Anything else?**

R:	Mine is a question if you confide to someone about your status and they post it on social media like Facebook is there any measure that I can take?

**M:	Yes, there are legal ways we will discuss later.**

R:	That is illegal you are not supposed to disclose someone’s status.

**M:	Okay.**

R:	I think it would be better to be told so that you can have that information at your fingertips.

**M:	Do you at times feel like you don’t want to take your medication?**

R:	Yes,

**M:	What circumstances drive you to feel that way?**

R:	When am tired.

**M:	Any other circumstances?**

R:	monotony of medication.

**M:	How about a break up with your boyfriend or when you disagree with your mum?**

R:	I always disagree with my mum,

**M:	Would that discourage you from taking your medication?**

R:	Yes, like my mother talks really negatively about my drugs at times I feel discouraged to take them, and if later on she comes to ask me if I have taken medication I will just lie to her.

**M:	How about breakups would that discourage you from taking medication?**

R:	No.

**M:	You had earlier on mentioned that you cannot take these drugs when you are in a crowd?**

R:	Yes, especially with some strangers.

**M:	Anything else?**

R:	And also the taste can discourage you.

**M:	Any other reasons?**

R:	When you sleep early and forget about the drugs.

**M:	What about if you are in boarding school verses day school would that discourage you from taking your medication?**

R:	Yes, a very big difference.

**M:	How?**

R:	In boarding school we are always together but in a day school I have time alone at home to take my medication.

R:	Or even everyone at home knows about your condition so you don’t see any need of hiding unlike in boarding schools where people bombard you with questions like why are the tablets so huge, why do you take medication daily and so forth.

**M:	So you would rather be in a day school than boarding school?**

R:	Yes.

**M:	Do you feel supported about your HIV status at home?**

R:	Yes, I am.

**M:	Anyone else?**

R:	I feel supported.

**M:	Who supports you at home, some people had mentioned mothers, parents of course, sisters, cousin, anyone else who supports you at home?**

R:	The most support we receive from our families and parents to be specific.

**M:	In what ways do they support you?**

R:	They encourage you and motivate you when you have lost hope and so forth.

**M:	Anyone else?**

R:	To me the best support is they have never treated me differently.

**M:	And you?**

R:	I get the best support at school because when am at home and my mother asks me if I have taken medication I always lie to her.

**M:	Someone else?**

R:	I think the best support is from mother she makes sure that I take the medication right infront of her I cannot just tell her that I have taken and she believes me.

**M:	Okay.**

R:	She has to make sure I have swallowed it and I open my mouth so that I don’t hide it. to me I think that is the best because were it not for her I think I would have given up at some point because at times I really feel like am tired of taking the medication but she has been really supporting me.

**M:	So she has encouraged you by ensuring you take the** medication?

R:	Yes.

**M:	Any other form of support apart from encouragement, being treated the same and your mum ensuring that you have taken your medication?**

R:	Mine is similar to hers, my mum always ensures that I have taken my medication.

**M:	Is there anyone who feels like they don’t receive sufficient support from home?**

R:	(Silence)

**M:	And once you complete high school and college you do you feel that you are now ready to face the world and go achieve your goals and dreams and succeed in life?**

R:	Yes.

**M:	What makes you feel that way?**

R:	When I see people doing what I have always dreamt of doing like when I see people singing that motivates me and it makes me feel that I can also be able to achieve my dreams and be better or equal to that person.

**M:	Anyone else?**

R:	If you want to achieve your dreams of course people will talk to you and encourage you but as an individual you must remember to take your medication as you think about your future. And you should not feel that since you are taking those drugs your dream will not come true. So you have to personally encourage yourself and see the future you want to have.

**M:	Okay.**

R:	And if you come across other people who are sick or in similar circumstances like yourself you can encourage them. So, I think you need to have that self-drive.

**M:	So there is that self-drive and you must have that?**

R:	Yes.

**M:	Anyone else?**

R:	Self-acceptance once you accept yourself and your condition then everyone else will accept your condition as well. The moment you have not accepted your condition then things will not feel right but once you accept yourself then everything will feel okay. As you walk around nobody can tell if you are no medication or not unless you neglect yourself so once you accept yourself you will join the rest of the society and enjoy and live your life.

**M:	Anyone else?**

R:	I believe the only difference between us who are HIV positive and those who are negative is the fact that we take medication, there is nothing else that differentiates us. Like I always say that I would like to be a pediatrician because one time my viral load was high, and were it not for those who encouraged me telling me that there is no difference between me and them and it was only the medication that I was taking that was differentiating us. So, I have been motivated to realize that someone somewhere will benefit through me and if I don’t take my medication I will not live to see my dreams, that is why I feel that people should not feel that they might not be able to fulfill their dreams because we can be anything we dreamt of if we put our minds to it. Even when you work as a pilot nobody among your passengers can know your status if you don’t share with them so it’s upon us to keep going. And I keep telling people that we should not feel low about ourselves just because we are taking that medication. Those pills do not make you less of a person. If we hadn’t told you about our status am sure you would not be able to tell, am sure we don’t even look sick actually we look healthier than the ones who are not taking drug.

All:	(Clapping)

**M:	That was well narrated. So thinking of your future what challenges do you foresee?**

R:	Self-drive.

**M:	Any other challenges?**

R:	Having to take medication for the rest of our lives or until we die.

**M:	Any other challenges you foresee, thinking of your aspirations, dating, and marriage and having kids?**

R:	Coming to the hospital all the time.

**M:	Any other challenges?**

R:	Disclosure to friends, boyfriends, husbands.

**M:	Any other challenges you foresee?**

R:	It depends there are some very good guys that if you tell they will be so supportive and there are some that if you tell they will start isolating you.

**M:	And previously they treated you well?**

R:	Yes.

**M:	Any other challenges?**

R:	The future if we have babies and maybe your child is negative and you are positive. And as they grow up they observe that you are always taking medication and they might be curious to know why and at some point you have to disclose your status to them.

**M:	Okay.**

R:	But I don’t think I would want to tell my child; why tell her it will just stress her out.

R:	But you cannot hide the secret forever I believe at one point you have to tell them the truth.

**M:	Anyone else?**

R:	I wouldn’t want to tell her when she is very young. If you tell her and she is very young she might tell her friends and there could be other repercussions.

R:	But thinking of how things are going on, I think they will be more informed much earlier than we did. They might even find out before you tell them.

**M:**

**M:	Okay so, would you like to be prepared for your future and how you would handle these challenges? Or do you feel that you will handle them at that point?**

R:	I don’t think you can see fire burning and wait for it to burn you, you have to face the challenges head on.

**M:	So you feel like you will tackle those challenges at that moment they present?**

R:	Yes.

**M:	Have you experienced any form of stigma?**

R:	What do you mean?

**M:	Stigma is when someone looks at you and judges you based on your current state any form of humiliation you have faced and the treatment you received as a result of that stigma?**

R:	No, I haven’t.

**M:	Anyone else?**

R:	Most people I hang out with are not positive so you might be sitting somewhere and they start discussing about HIV people and they describe the pills and I cannot show them my pills because they seem to know about those pills and if I show them they will start telling everyone about my status that am HIV positive.

**M:	Any other experiences?**

R:	At times some people go on social media to talk about people who go to CCC that they are HIV positive and they normally go for their medication there and such things really make us uncomfortable especially if you have not disclosed to everyone about your status.

**M:	Okay.**

R:	Like I came with my brother and he didn’t know my status and he just waited for me but he saw the initials CCC but he didn’t know what I was coming to do, but when he saw what someone had posted on social media about CCC am sure he put one and two together.

**M:	So you think he knows about your status?**

R:	Yes, he does but we have not discussed about it.

**M:	But you cannot ask him?**

R:	No, if I do it will bring other conversation.

**M:	But that is your brother?**

R:	Yes, he is but most people don’t know what the initial CCC stand for but when he sees people on social media talking about CCC it’s like everyone will know that anyone who goes to the CCC is HIV positive.

**M:	What if you went to visit someone?**

R:	They will not think that way.

**M:	So that is a challenge?**

R:	Yes.

**M:	Okay.**

R:	I have told my friends that I go to the clinic but they have never known what I go to do there. Most of them think that I have issues with the mumps that I had before, so they think that I still go to seek further care with the mumps but they don’t know that I go for HIV treatment. I don’t want to feed them any more information about me they can keep guessing about my life for all I care but I will not share with them because I don’t know what they will do with that information.

R:	Are you afraid?

R:	I just tell them that am going to the clinic and if they want to know more or they are	too curious then they should come with me to the clinic.

**M:	Okay thank you for your time, we have learnt a lot, at least everyone has shared.**

**END.**
