# Supplementary material for: Psychosocial needs among older perinatally infected adolescents living with HIV and transitioning to adult care in Kenya
Source: PLoS One. 2020 Jul 29;15(7):e0233451. doi: 10.1371/journal.pone.0233451 (PMC7390380; doi:10.1371/journal.pone.0233451)
Supplement: S1 File — (ZIP) [file pone.0233451.s002.zip › uploaded final PLOS/Reviewed Transcripts/FGD5.doc]

**F :	We can begin and another thing is I would ask you to be audible so that we can be able to listen to what you are saying. So, the first question is how did you cope emotionally when you found out about your HIV status? How did you feel? Were you angry, were you sad, you were okay, how did you feel?**

R1:	Truthfully it was painful.

R2 : I was sad.

**Mod: Okay sadness it was painful?**

Res: It was unbelievable.

**Mod: Someone else, how did you feel? I am sure there was a way you felt which was different for everyone?**

Res: It was bad.

Res: Shocking.

**Mod: Shocking, painful, bad, anyone else? So, maybe I would ask when did you find out? As in how old were you when you found out?**

Res: I cannot quite remember but I was in class six.

Res: Twelve years old.

**Mod: That was class?**

Res: Seven.

Res: I was eleven.

**Mod: Yes.**

Res: Nine.

Res: Eleven.

Res: Ten.

Res: Thirteen.

Res: Nine..

Res: Nine

Res: Thirteen.

**Mod: So, nobody found out when they were young like three years old?**

Res: Yes.

**Mod: Alright who told you?**

Res: The doctor at Kenyatta.

**Mod: The counselor?**

Res: Yes.

**Mod: And you?**

Res: The counselor.

**Mod: Okay.**

Res: My mum.

**Mod: Is there anyone who was informed by anybody other than their mum and their counselor at the hospital? Okay, alright. So when you were informed what were some of the things that you wished during that time you had been told that maybe were not tackled at that time? What are some of the things that you wish you were told, that if you were to be the counselor at that time you would have told someone? Or you wish this information you had at that time? Maybe about the drugs, maybe about the future, maybe about anything, what is it that you wish you had been told but you were not told?**

Res: The future.

**Mod: You wish that you were told about the future?**

Res: Yes.

**Mod: What else do you wish you had been at that time but you were not told? Anyone else, yes (name)?**

Res: The drugs.

**Mod: What about them?**

Res: Why I was taking the drugs but not getting better.

**Mod: Okay, someone else, anyone? What do you wish you were told looking back that the information was not provided? What do you wish you were told like for example about the drugs that you didn’t know then but with time you have come to find out? Anyone, just feel free nothing. What do you wish you were told at that time you found out?**

Res: My future.

**Mod: What about it?**

Res: When you were told that you are positive you feel as if your future is coming to and end.

**Mod: So you wish that you were told?**

Res: That my future is going to be bright and I will succeed.

**Mod: Yes, you what do you wish you were told?**

Res: I wish I was told more about the drugs, the way the people suffering from malaria are given and they completely recover, I wish I had information about my recovery period.

**Mod: Okay, what else, (name)?**

Res: Drugs.

**Mod: What about the drugs? What do you wish you were told about the drugs?**

Res: As in, I should continue taking them but maybe eventually a cure would come in future.

**Mod: What do you wish you were told?**

Res: About the drug.

**Mod: What about it?**

Res: I was told it was not the end of it all.

**Mod: So they did tell you?**

Res: Yes.

**Mod: And did they tell you for example how long you have to take the drug?**

Res: No.

**Mod: Is that something you wish you were told maybe?**

Res: No.

**Mod: Okay, are there times that you feel that you have had it with these drugs and what makes you feel like that?**

Res: The fact that they are big.

**Mod: What else, what about the drugs makes you feel that way?**

Res: The daily dosage.

**Mod: (Name)**

Res: The daily dosage.

**Mod: What else?**

Res: About the daily dosage.

**Mod: What else?**

Res: I had an infection and I had to take other drugs, and I felt that I had too many drugs to take and it was boring.

**Mod: Having to take these HIV drugs and other drugs for the infection?**

Res: Yes.

**Mod: So, when you get sick and in addition you have to take those drugs and these drugs it is too much?**

Res: Yes.

**Mod: Thank you for that, someone else?**

Res: Having to take them in the presence of very many people.

**Mod: Okay, first of all when do you take your dosage?**

Res: At night.

Res: Morning and evening.

**Mod: Twice?**

Res: Yes.

Res: Morning and evening.

**Mod: Aha.**

Res: Twice.

Res: Twice.

Res: Twice.

Res: Twice.

Res: Twice.

Res: Morning.

**Mod: So, we have morning, then morning and evening and twice?**

Res: Yes.

**Mod: When are there many people around?**

Res: When I visit other people, or maybe I have gone camping it is quite challenging.

**Mod: Okay, she talked about having to take the drugs in the presence of many people and she talked of the time, what else?**

Res: At times the drugs taste disgusting while taking them.

**Mod: They taste bad?**

Res: Yes.

**Mod: Who agrees that they taste bad or some of you don’t feel like that?**

Res: I don’t feel that way.

**Mod: The rest of you don’t feel bad?**

Res: Yes.

**Mod: Are there some side effects of the drugs, anyone side effects of the drug?**

Res: (Silence)

**Mod: Are they many, does anyone feel that they are many to take?**

Res: Yes.

**Mod: How do you feel?**

Res: I was told that I was going to be given one drug so that I can be taking one drug because at the moment I take three dugs a day for morning and evening.

**Mod: So you feel?**

Res: They are many and I have never understood why some people take one drug while I have to take three drugs.

**Mod: Okay, anything else, are there some of you in school?**

Res: Yes.

**Mod: How do you deal with that? Yes, I can see there is a story there?**

Res: I take mine at 6:30am and am free at that time because its breakfast time but it’s a challenge taking them in the evening.

**Mod: Okay, anyone else in boarding school? Is it a boarding school?**

Res: Yes.

**Mod: Anyone else how do you deal with that (name)?**

Res: I take mine at the matron’s room.

**Mod: So that is not a problem?**

Res: No, its not.

**Mod: Okay, anyone else?**

Res: I take mine at the nurses’ station.

**Mod: Alright, how about when you are not at home when you are supposed to take the drugs how do you deal with that?**

Res: You always make sure you keep time and you are home when you are supposed to be taking the drugs.

**Mod: Okay,**

Res: I make sure that am at home by 8:00p.m.

**Mod: So, that is how you cope with the drugs?**

Res: Yes.

**Mod: Anyone else how do you cope with that?**

Res: For me, if the time for taking my drugs is not yet reached and am not around I always say that it’s better to take late than never.

**Mod: So even if you are slightly late you have to make sure that you take them?**

Res: Yes.

**Mod: How about you, are you still in school?**

Res: No, I completed.

**Mod: But when in school, how do you cope with that story or it was not a problem?**

Res: It was not a problem.

**Mod: Why?**

Res: I had established a good time to take them.

**Mod: Okay, let us move on and do you feel that you are supported about your status at home or by anyone else that you need their support? Do you feel supported at home?**

Res: Yes.

**Mod: At home are you supported?**

Res: Very much.

**Mod: Anyone who feels that the support system is not great?**

Res: Yes.

**Mod: I can see a story there, tell us?**

Res: I feel supported.

**Mod: Who is that person that regardless of the situation you know they have your back like they support you fully?**

Res: My dad.

Res: My mom.

Res: My mom.

Res: My mom

Res: My dad.

Res: My mom.

Res: My mom.

Res: Both.

Res: My dad.

**Mod: So, the support is good?**

Res: Yes.

**Mod: And what about anyone else that you interact with, if you are in school your teachers, students or work mates how is the support there?**

Res: Not very good.

**Mod: How is the support?**

Res: If they are not aware that you are HIV positive they will not support you and if you tell them about your status they will avoid you.

**Mod: So, there are specific people you confide in?**

Res: Yes, I have one but she is also positive.

**Mod: Your friend?**

Res: Yes.

**Mod: So overall how is the support?**

Res: Mine is good.

**Mod: Anyone who feel there is a place they don’t get support, tell us about that, because it happens.**

Res: In school, there are those that start talking about some diseases and maybe you are sitting in behind them or next to them and they start to bad mouth the people who are infected for instance those infected by HIV and you feel really bad.

**Mod: Okay so that is not very nice, thanks for that, anyone else (name)?**

Res: Nothing at all.

**Mod: The support is good?**

Res: Yes.

**Mod: Remind me your name, yes (name)?**

Res: My support is good.

**Mod: Do you go to school?**

Res: I completed.

**Mod: And, how are things in school?**

Res: I was a day school and the challenge I faced was people would ask me why I had to come to the clinic every middle of the school term and I would lie to them that I had chest problems and I had to come for checkup.

**Mod: And the teachers?**

Res: Only the principle knew about my condition.

**Mod: How is your support system or where don’t you receive support?**

Res: I have a good support system and while I was in school only the principle was aware of my condition.

**Mod: Okay.**

Res: But when I had to come to the clinic some students always asked me where I had been and I would just tell them that I had to go to the clinic because I was sick but I could not tell them exactly what treatment I was coming for.

**Mod: And have you ever disclosed to anyone but they didn’t take it very well?**

Res: Yes.

**Mod: Who was that?**

Res: I told someone that I was HIV infected but they didn’t take it very serious.

**Mod: So, was this a friend, a boy friend?**

Res: A girlfriend.

**Mod: A friend who is a girl?**

Res: Yes.

**Mod: Anyone, (name)?**

Res: No.

**Mod: You have never disclosed your status to anyone and they didn’t take it serious?**

Res: I disclosed to someone and they took it positive.

**Mod: That is nice, and you?**

Res: I have never disclosed to someone.

**Mod: Yes.**

Res: I have disclosed to my close friends.

**Mod: Tell us about it?**

Res: My desk mate didn’t take the message positively and she stopped interacting with me the moment I disclosed my status. And then after sometime she realized that what she was doing was not right and she came and apologized to me.

**Mod: Anyone else, (name) have you ever disclosed to anyone who didn’t take the news very well?**

Res: No.

**Mod: The support has been great?**

Res: Yes.

**Mod: Okay, so the next question is do you consider or at this point do you have confidence that as you are have completed school and you are going to the outside world and joining the job market you are confident enough that you will succeed as a person who is positive? How do you feel about that as a HIV person who has cleared school and you are going outside to the job market?**

Res: It might seem hard to believe that although one is positive you can still succeed in life but I know that even though am positive I will still succeed in life no matter what.

**Mod: Okay.**

Res: To me being positive doesn’t mean anything to be because being positive and someone out there being negative I believe that I should be able to do what the negative person is capable of doing.

**Mod: What they do in terms of?**

Res: Whether it is a career or educational am also capable of doing the same.

**Mod: How do you feel?**

Res: Being positive doesn’t mean that am incapable of doing other things so I believe that I will succeed.

**Mod: So (name) what do you think?**

Res: I don’t think being positive brings a setback in my life.

**Mod: Okay and do you feel that you can be given training about life skills, general training that will make you feel ready for the future? Would you be interested in such a training?**

Res: Yes.

**Mod: Training would be nice?**

Res: Yes.

**Mod: About what, what kind of training would you like to receive? Marriage, dating?**

Res: Marriage.

**Mod: What else?**

Res: About having a family.

**Mod: Okay, (name)**

Res: Having a child.

**Mod: And a child who is?**

Res: Negative.

**Mod: Great. Although we had talked about this briefly but have you experienced stigma?**

Res: Yes.

**Mod: Tell us about that?**

Res: Yes, in my former school.

**Mod: Tell us about that what happened?**

Res: They used to steal my drugs in school.

**Mod: They used to steal your drugs?**

Res: Yes, and I could not tell them to bring back the drugs because they might feel that you are really in need of those drugs but in reality I really needed those drugs.

**Mod: So how did you deal with that?**

Res: I had to report to the teachers and that caused me to delay taking my drugs.

**Mod: Someone else, stigma and how did you deal with that?**

Res: I told my dad about that and I transferred from that school.

**Mod: So, you moved to another school?**

Res: Yes.

**Mod: Stigma anyone if it happened to us, you told us about your desk mate?**

Res: Yes.

**Mod: Okay, tell us.**

Res: There was this day I had taken my drugs and then I took them earlier and placed them inside the locker and so my friend found it and she asked me what they are for and I was like what do I tell her? And then I told her, well I had to lie to her that it was for headache and I felt bad because I thought if she would have known she would have discriminated herself from me.

**Mod: Okay, is that something that you wish you would have been told when your status was disclosed to you, like how to deal with those situations, is that something you wish you were told about?**

Res: Yes.

**Mod: Okay, someone else stigma what was the drama?**

Res: My drama was that one time, my primary desk mate saw me taking the drugs and then she asked me why I kept on taking drugs and I had to lie and tell her they were supplements and she made a comment like taking too many of those supplements is not good for your health and one time it will cause your demise and I was shocked.

**Mod: You were shocked?**

Res: Yes.

**Mod: Anyone else (name) stigma and how did you deal with it, how did you cope with it?**

Res: I used to take the drugs at the nurse’s offices and some people would walk in while I was taking the drugs and some would ask me what are these drugs for and even some asked me why I had all those drugs and what were they for and I had to lie to them that I had an infection or something.

**Mod: Okay, so did they eventually find out what they were and were you stigmatized?**

Res: It is very difficult for someone to find out in school.

**Mod: Okay, stigma?**

Res: Sometimes you attend camps and while taking drugs the other people are curious to know what the drugs are for and am not sure what to tell them.

**Mod: (name) stigma and how did you deal with it, how did you cope with it?**

Res: There was a time recently before we started the KCSE exams, a classmate a boy went around disclosing that I (name) was suffering from HIV and so unfortunately it happened that I developed an allergy and I developed some sores and so even greeting people was a challenge because people were afraid of shaking hands with me and I could hear everyone saying that I **(**name) was HIV positive, so I had to miss school for a few days for people to get silent and then I came back.

**Mod: Okay.**

Res: And so when some people confronted me for the truth I just insisted that I had an allergy I never confirmed anything to them.

**Mod: And how did you cope with that?**

Res: I just persevered.

**Mod: Okay (name) stigma and how did you deal with it, how did you cope with it?**

Res: Many students were curious what drugs I was taking and for what ailment and so I just disclosed to them that I was HIV positive.

**Mod: Where was that?**

Res: In school and with time some made fun of me and some didn’t and I just assumed them.

**Mod: So you coped by telling them the truth?**

Res: Yes, and they eventually stopped talking.

**Mod: Anyone else stigma and how did you deal with it, how did you cope with it?**

Res: There was a day we had gone for a camping trip in Kajiado and I had carried my drugs.

**Mod: What camping trip.**

Res: Young life.

**Mod: Okay.**

Res: And so we used to spend in a tent and we were three people in a tent and so my best friend cheeked my bag and found my drugs and she asked me what the drugs were for and I told her they were for a headache and some for my chest as I had chest infections and I told her to stop touching them because I really needed them.

**Mod: And did they finally find out what they were for?**

Res: They never found out.

**Mod: Anyone else, who has had a bad experience or stigma and how did you deal with it, how did you cope with it?**

Res: No.

**Mod: Anyone else a bad stigma, anyone?**

Res: No.

**Mod: I can see a story there.**

Res: No.

**Mod: Okay we are almost done anyone with any questions, comments.**

Res: Is it bad to have a boyfriend when you are positive?

**Mod: Okay, would you like to take that one now or…**

**Mod: Let us take all the questions and answer all of them.**

**Mod: Is it bad to have a boyfriend, okay bad in like. Of course it’s not bad but what do you mean by bad, do you mean like to engage in sex?**

Res: Yes.

**Mod: Any other questions.**

Res: If you have a boyfriend how do you disclose to them because it’s very hard.

**Mod: Finding the right time and the right words?**

Res: Yes, the right words to tell him.

**Mod: Anyone else with a question, something you have always wanted to know, we have a specialist here.**

**Mod: I am here to answer any questions you have on contraception, sex, disclosure, so if you can write them down am here. Yes (name), (name), nothing, anything.**

Res: I have always wanted to know okay I was told that you are capable of getting a HIV negative baby but if I end up with a partner who is HIV positive is it possible for us to have a HIV baby?

**Mod: Very good question, any other question family, marriage, like she mentioned contraception.**

Res: I would like to know how I can still be going for clinics and still be getting my drugs supposing I was to maybe travel to another country.

**Mod: Very good question how do you get your supplies there?**

Res: Yes.

**Mod: Any other questions (name), (name), (name) (name), you don’t have any questions? And please drink your juice, it was for you to drink before lunch, any other questions, comments.**

Res: I think sometimes at home some people suspect and they keep asking me about these drugs and some have observed that my immune system is weak and I get sickly all the time so how about those people, how do I cope with that because they talk about me?

**Mod: Any other questions, comments.**

Res: I would like advise on how to help a friend who is HIV positive, I think she already lost hope.

**Mod: Is it the same friend you were telling us about?**

Res: Yes, I believe she lost hope and she stopped taking drugs despite the mother encouraging her to continue taking drugs and she responded that if it’s because of death, there is no need she will just die.

**Mod: How old is she?**

Res: He is 17 years.

**Mod: Okay.**

Res: And he attends this clinic.

**Mod: I will tell the doctor to see how he can be helped.**

Res: But don’t disclose my identity.

**Mod: I will not do that, that will never happen.**

Res: I also have a sister who is HIV positive but I have never seen her taking drugs.

**Mod: Is it a big sister or small sister?**

Res: She is my big sister called **(**name), her case is known and even last year she was admitted.

**Mod: She doesn’t take drugs?**

Res: Totally, she only takes the drugs when she is severely ill.

**Mod: Okay, very good with the concerns.**

Res: And if you were close to someone and they ask you to go for a HIV test …

**Mod: Is it your boyfriend or?**

Res: A boyfriend and I keep postponing the issue.

**Mod: Okay, anything else?**

Res: (Silence)

**Mod: Thanks for coming and attending our interview, we will wait for the doctor to come and respond to your questions.**

Res: Okay, thank you.

**Mod: Welcome.**
