# Supplementary material for: Psychosocial needs among older perinatally infected adolescents living with HIV and transitioning to adult care in Kenya
Source: PLoS One. 2020 Jul 29;15(7):e0233451. doi: 10.1371/journal.pone.0233451 (PMC7390380; doi:10.1371/journal.pone.0233451)
Supplement: S1 File — (ZIP) [file pone.0233451.s002.zip › uploaded final PLOS/Reviewed Transcripts/IDI1M.doc]

**M: Okay, I think we are good to go**

R: Yes.

**M: How long have you been coming to this clinic?**

R: Since I was a kid.

**M: Can you remember the earliest time you came?**

R: Pre-unit.

**M: Around how old were you?**

R: I cannot remember.

**M: Pre-unit is maybe 4, 5 years?**

R: Yes.

**M: Do you come on your own now?**

R: Yes, I do.

**M: Have you been able to get used to the clinic and the way it works, coming, registering?**

R: Yes.

**M: What is the first thing you do when you come?**

R: I register first and then they take blood pressure, and I go for consultation.

**M: Do you remember the time you were told about your status and the reason why you are taking drugs?**

R: Yes, I remember.

**M: How old were you?**

R: Around 13, 14.

**M: Standard, 7, 6?**

R: Yes.

**M: Who told you?**

R: My mum told me before we came to the facility; she told me that morning before we came to the hospital and when we got here Lillian explained further.

**M: How was your reaction?**

R: It was not good, and I kept thinking about this life of having to take medicine the rest of my life but after counseling I went back home pretending to be happy but deep inside I was not happy.

**M: At the end of the time did you eventually tell someone how you feel tor you just kept it with you?**

R: I didn’t tell anyone because I didn’t want anyone to know what was going on deep inside.

**M: Why, I am just curious.**

R: After my status was disclosed to me, I wanted to lock myself in the house, I didn’t feel like coming out of the house and at that time there was a lot of stigma that I didn’t want to hear about and that most people did not have the information. But now I feel better and that is due to talking to several people over time.

**M: It made you feel different?**

R: Yes.

**M: And before you were told you were normal?**

R: Yes, before I was told I was social and I had no fear but after being told my status, I became less social and more quiet.

**M: That was your reaction?**

R: Yes.

**M: What brought about that reaction was it how people were reacting out there or what made you to withdraw?**

R: Before I used to hear people talk about HIV but I didn’t understand the reasons why I was taking the medicine. I would even tell people that I take medicine for cold.

**M: Did you know HIV at that time?**

R: I did but I didn’t imagine I had it, I lost hope but I didn’t want to show my family and I had to know more about it. People saw me acting normally but inside me I was a totally different person.

**M: How long was that?**

R: 3 years.

**M: You even remember how long you felt like that?**

R: Yes.

**M: What changed when you started being more social, what made you stop feeling the way you were feeling?**

R: I decided to be normal because if I act normal then nobody can tell my status just by looking at me. And if you start talking about that topic I have a choice of either exiting the place or just contributing or not contributing at all. And since I love music I can go out and sing some music or attend drama club to keep me busy until it became normal and nowadays I just take medicine in the morning and evening and it’s no longer bothering me. Nobody asks me any questions and if someone starts discussing that topic then I move out of that place because I don’t want to discuss that topic.

**M: Did you attend boarding school?**

R: No, I was in day school but the school was located in a certain ghetto and so I had to worry about me and everyone minded their own family including my dad and mum.

**M: Is your mum taking ARV’s?**

R: Yes.

**M: How about your dad?**

R: Even my dad.

**M: Anyone else taking ARV’s in your home?**

R: No, just the three of us.

**M: Are you the last born?**

R: No, I am the first born

**M: And you have other siblings?**

R: Yes, but they are negative.

**M: At least by that time mum knew what to do to prevent them from being affected?**

R: Yes, I was admitted here in Kenyatta for a while and then I was transferred to this facility.

**M: But when you were young they never told you what you were suffering from?**

R: No, because when you are young you can talk and disclose your status so they lied to me that I was taking drugs to cure a cold.

**M: But you now understand why they didn’t tell you?**

R: Yes.

**M: Do you feel that you would have been told earlier than 13 years or later than?**

R: After completing primary when I join secondary because had I been told when I was in class 4 or 5, I would have even run away from home. I had that habit of disappearing from home so maybe it would have been made worse. At that time I was very active and social and who know what I would have done.

**M: And in your opinion that first day you went to the counselor and your status was revealed to you, do you feel that there is something which would have been revealed to you then that would have made you feel better? If you had to do it again what things do you wish you would have been told?**

R: There is nothing, they told me everything and before I got here my mum told me everything that I was going to be told and when I got here it was quite easy. She told me that I was suffering from a certain disease but the counselor would expound on the same. And my mum made sure that day I was very happy so that I don’t over react.

**M: Did you ask any questions?**

R: Yes, I asked questions about ladies.

**M: What questions did you ask?**

R: About engaging in sex if I can spread the disease to my girlfriend because at that time I was not well informed and I was told that I can infect her. So when I was in high school I avoided socializing with girls because I might be involved with a girl and maybe she wants to get intimate and I don’t want to infect her so I decided to abstain all together. And if you decide to not get intimate with that girl they might feel that you are a weak, worthless man. So up to date I avoid being intimate with any girl, we just talk but I have decided to abstain

**M: Any other questions you asked?**

R: I asked why they had decided to tell me now and not later but I thank God for the counseling because I am now more informed and I can educate others.

**M: It took you like 3 years to cope and be able to take medicine without feeling anything?**

R: Yes.

**M: What made you cope other than keeping busy, going for drama, did you talk to anyone or open up to anyone? You also mentioned you used to be very social but did you talk to anyone?**

R: I had this friend, I was older than her but she was a good friend that I trusted could keep my secret and she has kept it a secret to date. She told me one story that really motivated me because there are other people in worse situations, some people were involved in an accident and they were paralyzed, others are suffering from cancer and while in hospital I remember one patient who was sick that just died and so that motivated me that my situation is not very bad and if I continue taking medicine then I will live a long life. There is no need of stressing myself and since that day I have not felt so bad.

**M: When you saw that patient who died?**

R: Yes, he died while I was in hospital and I remember while in xxxHospital, many patients died and only two of us survived in that ward and one is paralyzed and I am the other one. So that motivates me knowing that if God spared me all those chances then I need to keep living and stop blaming myself or anyone.

**M: Okay.**

R: So in Kenyatta I saw many incidences, even though I was still young, I was in class 8 but whenever I left home coming to Kenyatta, it dawned on me that many people are suffering and some are in worse situations than me. I have been taking my medicine well and I have not had any relapses.

**M: Did you start you clinic in there a before being transferred here?**

R: I was suffering from meningitis and I had not yet started taking my ARV’s and even my dad and mum had not yet known their status and when I got sick and I was tested then even my parents and siblings had to be tested.

**M: Which class were you?**

R: Pre-unit and then I came to this facility.

**M: You remember all that to date?**

R: Yes, I do remember. I had gone to a coma and I remember before going to the coma I asked my dad if I was going to make it and he assured me that I was going to make it. though I was in a coma for 18 days that is what my dad told me and he stayed with me for all that time and when I came to of that coma I found him right there next to me and since that day of the coma, I have been feeling good.

**M: 18 days?**

R: Yes.

**M: What else makes it difficult for you to cope, you mentioned the ladies and the lack of sex, what else makes it hard for you to cope?**

R: I also act at the National Theatre and then at times I am on stage and it’s time for me to take my medicine and maybe I don’t have a phone next to me to remind me and I cannot get out of the stage because I am performing. At times after the performance you get fans coming to you to congratulate you and so I end up missing taking pill that day or the day after due to the same function and I cannot take before the required time because I might feel dizzy on the stage.

**M: You take one pill once a day?**

R: Yes.

**M: Do you go to National Theatre daily?**

R: Not daily but there are many gigs, I am an actor and singer and I am a music student in Westland’s at Double Black and we might go to the club, maybe I go there as a Deejay and I cannot leave to go and take medicine, so that was my biggest challenge.

**M: What is your exact time that you should take medicine?**

R: 10:00am because I am usually free most of the time at 10:00am but it becomes a challenge when I have a gig.

**M: Most of the gigs are over the weekends so Monday to Thursday you are normally safe?**

R: Yes, I am. At times I travel for a whole week and it’s difficult to take medicine.

**M: There is no way you can sneak to the toilet?**

R: It just becomes difficult at times once you check in then it’s you and the entire crew you don’t get time alone.

**M: You don’t go to the toilet?**

R: I don’t get time at least at that time I might have to go after performing.

**M: So that is a challenge?**

R: Yes.

**M: Anything else?**

R: We moved back home in Juja and I know some people who had been stigmatized and they had even contemplated suicide and you know people in that region are not well educated.

**M: How old was that person?**

R: She was 16 years.

**M: She also got infected as a child from the parents?**

R: Yes she did and the mum delivered her and she died shortly after birth but their father deserted them and so the granny has been raising her and sometimes I help her and even take her out to seminars so that she can be more informed, because there are people who feel that once you are HIV positive, then your life is over. So I have been trying to help her because at times people point when they see us talking or walking together but I have been teaching her to ignore them because some of them are busy pointing and talking about us and yet they are not even sure about their status. So there is lack of education and stigma in my community but I like defending such affected people because they are very many but the funny thing is that I have not disclosed to them my status. The only people that know are my mum, dad and my siblings.

**M: Other than your friend and family members do you wish that you would tell someone else about your status?**

R: No

**M: Why not?**

R: It is not necessary not unless you need my help but I can give help without disclosing my status. What I do is defend the person being stigmatized, I might say something to the other person who was doing the stigmatization that they might even have no answer for me. I give them hard facts to make them realize what they are doing is wrong.

**M: In your opinion have you seen anyone dealing with the same issues that you are dealing with maybe they encourage you when you see how they manage the disease?**

R: I have not seen anyone like that, because personally I encouraged myself.

**M: There is no one you would refer to as a role model maybe someone who has gone through what you have been through?**

R: I don’t have any role models; I have lived doing things my own way and questioning myself if this is right or wrong. My father gives me advise that in whatever I do, it’s my life and I can either decide to build it or destroy it and that is the policy that I live by. At times before I do anything negative I always step back and remember that I am the one ruining my life and not any other person’s life and that has kept me grounded.

**M: Okay.**

R: For example I used to take drugs; there are no drugs that I have not used apart from the injectibles. I have smoked bhang, cocaine, all that due to stress and I would feel unloved because of my status and I felt like my other siblings were more loved and so, I would take drugs and then I realized being high is not a good thing and I stopped taking drugs while I was in form three. My viral load was quite high.

**M: There was a time your viral load was high?**

R: Yes, it was quite high because that time I never used to take my ARV’s and I would abuse drugs.

**M: Where did you get money to buy drugs?**

R: I was working as a Deejay since I was in form one and a thespian and I used to do such gigs and get money.

**M: That is how you used to cope with your stress at that time, by taking drugs?**

R: Yes and then my husband told me something that really scared me, he told me that even though I am abusing drugs, and avoiding medication, I will eventually die and I will be buried and he will buy rice and people eat and then I will be forgotten that really scared me. Then he told me that what he left behind is something that he will never do again so I keep wondering what was it he was talking about.

**M: Okay.**

R: So my viral load really went high and I would not stick to my appointments, I would skip medication and even at times I would not come for counseling I would just take the medicine and go home.

**M: Has your viral load gone to normal?**

R: Yes it is now undetectable.

**M: How do you feel about that?**

R: I feel good and I have been now monitoring my health keenly. I don’t need anyone to remind me and my dad told me that what I do now will determine my future so I have been taking my medication seriously and I always remember that I have to do good deeds I don’t care whether I will be paid for it or not. But I have been taking good care of my health. For instance if there is a gig and my appointment is on the same day I cannot choose to attend the gig and fail to come for my appointment, my health comes first.

**M: Do you still feel stressed?**

R: No, I no longer feel stressed unless my mum gets upset with me and she picks up a quarrel with me because at times she can be moody and when that happens I apologize and move away from the situation and I go play some music or do some activity that I enjoy and then I come back when she has gone back to her normal self.

**M: Where do you go to at that point?**

R: I go outside and play my guitar and we avoid that collision.

**M: Now when you need emotional support whom do you rely on?**

R: My dad.

**M: You have mentioned him a lot.**

R: Yes, I have lived with my dad longer than I have lived with my mum and he has tried to talk to me and guide me in the right direction. So my dad lives in xx and my mum lives in our xx and so I spend more time in Nairobi with my dad and most of the time when he sees me doing anything wrong he talks to me and advises me and he keeps reminding me to take my medicine because they will ensure I get a good future and also he teaches me how to cope with people.

**M: Yes.**

R: He also told me to avoid sharing my status with people, even if they open up to me I should not tell them my status because some people can talk and you never know how they might behave. The only person I should tell is my parents and my immediate family and according to him I should not tell neighbors. So most of the time he advises me a lot and he has never beaten me, he talks to me. He is not like my mum when my mum gets annoyed she beats me up but my dad talks to you and before he beats you, he must have warned you so many times and you were not listening then he will explode.

**M: Has your dad even beaten you?**

R: No, even when he heard that I was taking drug and I was involved with the wrong gangs he never got physical.

**M: Had you joined a gang?**

R: Yes and we used to take drugs.

**M: Did your parents know that you were involved in a gang and you were robbing people?**

R: Not at that moment when I was fully in it but he heard about it later on when I had come out of the gang, someone else told him not me.

**M: Who told him?**

R: A friend told him that he had seen me in a certain place which was not a good place and he suspected that I was involving myself with the wrong crowd. He saw me fighting with someone and he told my dad and my dad was like if it was self-defense then let him fight like a man. There were times I used to be very violent and I would fight a lot, there was a time I fought with a conductor and he never asked me about that, but these days I no longer engage myself with such things.

**M: So basically you have decided to live your life and you have not told anyone apart from your mum, da and your best friend that lady?**

R: Yes. So these days my conversations are based on where I am, if I am at work, then we focus on the work and when I leave work then I go home alone, I don’t like socializing with anyone. When I go to the gig we might go as a crowd but when I leave the gig I go home alone. And even where i love nobody knows my business I leave very early in the morning and come back late at night so I don’t know anyone personally. I don’t like anyone knowing how I live and trying to poke into my business, at the end of the day I just live alone and mind my own business.

**M: Do you live alone or with your parents?**

R: I live with my dad but he travels a lot so I feel like I just live alone.

**M: But whenever he is around he comes there?**

R: Yes.

**M: You don’t have friends?**

R: I have very many friends but not very close. I have one close friends but she is a lady that we have grown up together in the ghetto and they moved toxxx.

**M: Do you have a child?**

R: No, I don’t.

**M: Do you have a girlfriend?**

R: Yes.

**M: Do you get intimate?**

R: We have not discussed about sex and when the conversation goes towards that direction then I diffuse it right away.

**M: Have you ever experienced HIV stigma since nobody knows about your status?**

R: No, I haven’t because nobody knows about my status.

**M: I would like us to talk about ARV’s and at any point did you face any difficulties taking the ARV’s?**

R: I talked about the gig at times I don’t get time to take them.

**M: Any other reasons that make you not want to take medicine?**

R: I used to feel that way in the beginning but these days I am very committed I always take but incase I forget and remember too late maybe around 4:00p.m then I skip that day.

**M: What should be done to make it easier for you to continue taking your drugs? Like for instance when you are deejaying you can have a glass of water and just take the pill quickly and continue working?**

R:It is very difficult you might just decide to take it and a song is ending and you have to hype the crowd and continue playing the music.

**M: But you can time yourself when the music begins you just drink it very fast?**

R: At times its very tricky, there is a time I tried doing that and someone threw a bottle at me I almost got hurt.

**M: I think you should practice. I am sure there is someone a Deejay in the world, or in your position who finds time to take their medication despite their busy schedule at work.**

R: It is very difficult especially if there is an MC and the Deejay and the MC have to link up so that everything flows. If the MC is hyping up the crowd that is the time I can take the medicine but maybe it’s not the time that I should be taking my medicine.

**M: Thank you for that, and how about your mum and dad how have they managed to take that medication?**

R: Mum and dad I think are now used to taking the medicine over time, but at times my dad forgets and I remind him to take his medicine.

**M: You are his treatment buddy?**

R: Yes, if he is travelling for instance to Rwanda I reminding him to take his medicine and even to carry extra depending on the time that he will be away if its three or four days.

**M: Is he a truck driver?**

R: No, a bus driver and so I pack for him and put them in a jacket so that he can remember taking them. And then in the evening he calls me to remind me if I have taken medicine, he can even call me at 3:00, 4:00a.m reminding me to take my medicine and at times I could be deejaying and I forget to pick the phone but he will keep reminding me which is good.

**M: So let us talk about the future and currently you are attending the youth clinic?**

R: Yes.

**M: How do you feel about joining the adult’s clinic in the near future?**

R: I don’t want to come to that group because I will meet my parents and even that age group.

**M: You want to continue attending the youth group?**

R: Yes.

**M: Any concerns about joining the adult’s clinic, what challenges do you foresee?**

R: Sometimes they talk too much and I lose concentration.

**M: When do you lose concentration, at what point?**

R: When they all start talking and discuss then I feel like I cannot fit in their discussion and at times when they are talking I lose focus and start looking at my phone.

**M: When the youth are there do you talk to them?**

R: Yes, I do talk to them at least we can socialize.

**M: So you feel like you will not fit in?**

R: Yes and then they might talk too much but that is not the main issue, the main issue is not fitting.

**M: Where do you see yourself 10 years from now health wise, what is it going to look like?**

R: It is going to be normal because these days I don’t do drugs, I am not engaging in any vice and I don’t have any relationship with bad company.

**M: Are there goals you want to achieve in the next 10 years?**

R: Yes.

**M: What are your future goals?**

R: I want to be a prominent musician and do some music production.

**M: Like a recording artist?**

R: Yes, and if things go as planned then my life will be good.

**M: As you talk about being a recording artist and achieving all these goals, do you feel like your status might affect or stop you from achieving your goals?**

R: It will not affect in any way. I completed my school last year and I have faced many challenges that I have overcome so my status cannot stop me from achieving my dreams. I thank God because I know that every time I take the medicine it pushes my life with some days. I also take them knowing that in the future if I have a family my family will be healthy.

**M: Then would you like to have a family one day?**

R: Yes.

**M: Thinking about the family what are your future fears?**

R: There are no fears, I am confident.

**M: What are your goals around the family, are you looking for a negative person or negative person?**

R: Any person can fit as long as they accept me and love me.

**M: And your children what is your fear or hope around that?**

R: My fear is that they turn out positive like me.

**M: Do you feel you have enough information around that area how you can prevent that from happening?**

R: Yes I do.

**M: Like what?**

R: When my wife is pregnant, we should all come to the clinic and undergo checking so that the life of the baby is not ruined due to our carelessness.

**M: And then you said that you have chosen not to be sexually active but do you know what you can do if you choose to be sexually active? Something you can use?**

R: Yes, the use of condoms and PrEP but for now sex is not an issue to me.

**M: Really?**

R: Yes.

**M: As usual I hope my colleague told you that it’s a very confidential conversation and that is why I want you to be free I have worked with the youths and I know you dance but sometimes people are enrolled and the person introducing you in the process you might have sex with another person did that ever happen to you at any point in time and could that be the reason you are not too enthusiastic about it?**

R: It’s not like I have not engaged in sex, I have engaged in sex but there was a time when a time when another lady joked about being pregnant and I was responsible for the pregnancy.

**M: Had you used protection at that time?**

R: I had not used a protection but there was PrEP involved and she joked about that pregnancy and I thought if someone uses PrEP then no pregnancy can occur and those chemicals might destroy the sperms and no fertilization will occur. So when she told me that I completely died being responsible for the pregnancy but later on I felt bad that I had betrayed her yet I knew we had intercourse. So in the process I met her sister who asked me about it and I was defending myself saying that I am not sure if she had other boyfriends and then later on the lady came and told me that she was just testing me and it was a joke and she was not responsible but that really scared me the fact that I thought that lady was pregnant and I was going to be a father and from that day I don’t like talking about sex. When a lady starts talking about sex I diver to another conversation, and I feel like if we are in love then we will love each other but we have to avoid sexual talks for now.

**M: Do you think that will change?**

R: Yes, it will change, when I have a family I will enjoy having sex with my wife but for now most ladies are careless and I don’t want to be involved in that mess.

**M: So let us talk about something important and is there any information that you think would be helpful , in this program for information whereby we will have people coming once a week maybe in the month of April we will have some sessions are there specific things you would like to be covered that you feel that if they were in the program then you would feel that it is worth your time to come and listen?**

R: Yes, coming here is my first priority.

**M: But this one would not be for the medicine but for the program what would you like to hear?**

R: I would like you to talk about stigma especially in high school because once people know that you are infected then it will be really bad, because when people see you coming they will be like “he is coming” or something of that nature and you might tell them to stop doing that not because you have it but because you don’t want them to ever behave that way towards another person.

**M: How to deal with stigma especially in high school?**

R: Yes.

**M: How about in college?**

R: It doesn’t affect me much because I go back home in the evening.

**M: So mostly in boarding schools how to deal with disclosure and stigma?**

R: Yes, you might find that a teacher might know about it and she or he doesn’t keep his mouth shut and he comes to discuss about it in class and in my school we had such a teacher and so I never went for counseling in school. Even when I did a mistake in school I choose to go home rather than deal with the school counseling sessions.

**M: Any other things?**

R: How to deal with life outside and our medication

**M: How to cope with the different timings and schedules?**

R: Yes, how you can plan to take the medicine, maybe take it in the morning and the following day in the evening because my routine changes.

**M: Where do you get advise generally if you want to know the side effects of a certain drug or if you want to know about PrEP or anything?**

R: I go to the facility and most of the time when I want to ask a question I come here and get the answers right here.

**M: Any challenges you foresee that might stop you from attending?**

R: Work, but I am free Mondays and Sundays

**M: Any other challenges that might stop you from attending?**

R: If there is a lot of work then I cannot come because I need to finish all my work so that I can cover the other days.

**M: Is there anything you wanted to ask me that you wondered about and I am here and you can ask me?**

R: Are we going to be attending a support group?

**M: It is a support group because you have the same challenges but we refer to it as life skills program and we**

**M: There are things you have not told people or you have not talked about for a while and now are you feeling like your heart is lighter?**

R: Yes,

**M: There are things that you didn’t get a chance to talk about but then of course you are talking to someone who will keep this confidential because that is their work, that sometimes is good, because at times its good to talk about certain things so that some things get out of your mind. So xx I wish you very well and I want to call this interview to and end and we can go and have some tea. Are you hungry?**

R: Yes, I am.

**M: Lets go and see if there is some tea for us.**

R: Okay.
