# Supplementary material for: Psychosocial needs among older perinatally infected adolescents living with HIV and transitioning to adult care in Kenya
Source: PLoS One. 2020 Jul 29;15(7):e0233451. doi: 10.1371/journal.pone.0233451 (PMC7390380; doi:10.1371/journal.pone.0233451)
Supplement: S1 File — (ZIP) [file pone.0233451.s002.zip › uploaded final PLOS/Reviewed Transcripts/IDI2M.doc]

**M: So maybe we can begin with a brief introduction, tell me your name, what form are you?**

R: I will be joining form one.

**M: How do you feel?**

R: I am feeling good about it.

**M: Where will you be joining form one, or far away?**

R: It is within town.

**M: Is it a day school or boarding?**

R: Boarding school.

**M: Some people will be joining schools very far, you are lucky that you will be within, or you prefer going to upcountry schools?**

R: No, I am not used to that rural lifestyle.

**M: It is better when you are within its more accessible during visiting days and mid-terms?**

R: Yes.

**M: I would like us to discuss about your experience in this clinic, how has it been?**

R: It is good and when you get here they give you good service.

**M: What do you mean by good service?**

R: They talk to you nicely, counsel you and give you advice, if you skip an appointment they follow up and offer you counseling services.

**M: What happens when you go the counselor?**

R: She talks nicely to you and reminds you on the importance of coming to the clinic on your appointment date.

**M: What other advice does she offer?**

R: On your balanced diet and what you are supposed to eat.

**M: Do you also talk to her or she is the only one who talks?**

R: It’s been a while since I went to the counselors.

**M: But how is the experience when you come here is it interactive or are they the only people who talk while you listen?**

R: They just talk while I listen.

**M: Do you ask any questions?**

R: They normally ask the questions.

**M: What questions do they ask you?**

R: About my nutrition.

**M: Do you ask any questions?**

R: No, I don’t ask questions.

**M: Why?**

R: I prefer it that way.

**M: How long have you been coming here?**

R: Since 2014.

**M: Do you now understand the process when you come to the clinic and what happens?**

R: Yes

**M: Could you take me through the process, what happens when you get to the facility?**

R: I give out my card and they run tests like the weight and then I come to the doctor and they give me another appointment date and take medicine and go home.

**M: Do you decide on the appointment dates?**

R: No, they do write the appointment dates.

**M: Could you take me through the experience with the counselor the first time when you knew about your HIV status, how was it?**

R: There was a time that I feel really ill and my mum brought me to the hospital and they tested me and the doctor informed me about my HIV status.

**M: Who informed you was it the doctor or the counselor?**

R: I cannot remember clearly.

**M: When was it?**

R: 2014.

**M: And you were in which class?**

R: Class 4.

**M: And you were very young?**

R: Yes.

**M: Did you understand what you were being told at that age?**

R: No, I didn’t know but it was explained to me in 2015 and I understood it better.

**M: Who explained it to you better?**

R: My mum was asked to explain to me while at home and she did and when I came here they asked me if she had explained to me clearly and if I understood and I told them that I did.

**M: That was which year?**

R: 2015.

**M: Were you sitting next to your mum when she was told to explain it further to you?**

R: No, I was told to wait outside and when we got home she explained to me.

**M: But by that time you had an idea because you had been told when in class four?**

R: Yes, but I had not understood it clearly at that point.

**M: What did you understand about your condition when you were told in class 4?**

R: I didn’t bother but I continued taking medicine.

**M: Then in 2015?**

R: I continued taking the medicine but I was not very serious but in 2016, I became much more serious.

**M: What eventually made you to become serious?**

R: I would come and I saw many people and I realized that I was not the only one and I decided to take my medication without fail because that was the only option if I wanted to live, there was no other option.

**M: In 2015, did you used to take the medicine on your own or did you require guidance?**

R: I used to take the medicine on my own.

**M: Once you realized it was serious of course there are mental questions you have?**

R: Yes.

**M: What questions did you have?**

R: Why I had to be the only person who was infected in my whole family.

**M: Your brothers and sisters are not infected?**

R: Yes.

**M: Are you the last born?**

R: No, the second born.

**M: Is your first born infected?**

R: No.

**M: Then how many are behind you?**

R: Two

**M: Are they infected?**

R: No, they are okay.

**M: What questions do you ask then?**

R: Why me.

**M: How do you feel?**

R: I feel bad, at times I feel like asking my parents what happened but again I don’t want to confront them with such questions.

**M: But at times you feel like asking them?**

R: Yes,

**M: What questions would you ask them?**

R: Why me, what did they do? But I feel shy and I don’t know how to ask them and I just let it go.

**M: Do they feel like you have questions to ask them?**

R: I don’t think they do.

**M: Have they ever called you to tell you about what happened?**

R: No, they haven’t.

**M: But it’s not easy to deal with that wondering why you?**

R: Yes.

**M: How many kids are you in your family?**

R: Four.

**M: What questions do you ask for the counselor?**

R: (Silence)

**M: Are there questions you feel like asking the counselor then you just avoid asking them?**

R: Yes.

**M: Do you ask the counselor all questions?**

R: No, I don’t.

**M: Why don’t you ask all questions?**

R: I don’t trust them all, I feel that I might share with them something and they go and share with other people.

**M: Okay., the counselors?**

R: Yes, or you might meet them out there and they start telling other people your business.

**M: After knowing about your status are there issues you felt that they should have addressed during that time?**

R: Yes, the main one was how my future would be.

**M: That was the main question?**

R: Yes.

**M: What concerns do you have about your future?**

R: I hope that I will be able to get a good relationship because even these ladies also have other issues and they might not understand my situation.

**M: You don’t think the ladies will accept your situation?**

R: That might be tricky unless I find a very religious girl and an understanding church going lady but it’s difficult to convince the others. You might decide not to tell her but she will eventually know and that will not make things any easier for you.

**M: That is your main concern?**

R: Yes, it is.

**M: Any others, health wise?**

R: There is none.

**M: But those are the questions you have been asking yourself?**

R: Yes.

**M: How have you been copying emotionally since you knew your status?**

R: I really get uncomfortable especially in class when the teacher begins to talk about such issues. And since I am more informed about the issues I try to avoid contributing so much because it makes the other students wonder how you got to know so much about it, so you have to behave like you are ignorant yet you are not.

**M: How does that make you feel?**

R: Uncomfortable so when that topic comes up in class I have to be very quiet and not participate in responding.

**M: Any other things that affect you emotionally?**

R: Nothing, just that.

**M: Any other situations that made you feel uncomfortable other than the classroom setting?**

R: At times when I see a HIV positive person being discriminated upon I feel bad because I know that is my secret since I am suffering from the same condition tough I have not told them. And so that makes me feel bad because I feel like I might end up in the same position if I disclose to someone my status and then we get into a conflict then that person might also end up telling other people about my status.

**M: Have you seen that happening?**

R: Yes, in the estates.

**M: What happened?**

R: I don’t know what happened but I remember someone coming to tell me about another person’s health status and I felt uncomfortable since I had not disclosed my status. And that made me really feel bad.

**M: Did you ask why they were treating that other person that way?**

R: I didn’t want to ask because I might get them too curious, since I was in the same boat but it was a very uncomfortable situation.

**M: So the biggest challenge is you don’t want to create that suspicion upon yourself?**

R: No, I don’t trust any of those friends and so I have to be very careful and take care of my drugs and not just put them anywhere in the house because there are those friends that when they visit they walk around the house and they might come across them and even Google and find out what they are for. So I have to be very careful even in the house.

**M: If you compare now that you have knowledge about your status to before when you didn’t know about your status which one is better?**

R: After knowing your status.

**M: Why?**

R: If you continue living without knowing your status, then your viral load goes up and then things might become worse than they would have been if you started your treatment earlier on. But if you know and start with the medication immediately then you can continue living a normal life.

**M: So, it’s better when you know so that you control your viral load?**

R: Yes,

**M: In your life are you aware of anyone dealing with the same things that you are dealing with currently, someone who influences or encourages you?**

R: None.

**M: There is no one who encourages you because they are in the same situation?**

R: No.

**M: How about your parents?**

R: My parents are just okay, but at times they don’t take it lightly when I fail to eat well because they are very strict about my diet and me taking the medicine..

**M: Are your parents HIV positive or negative?**

R: They are negative and everything to them is just normal.

**M: Are they on medication?**

R: I am not sure, they have never disclosed their status to me and I have never asked them.

**M: You have never asked them?**

R: Yes.

**M: Why not?**

R: Everyone is busy with their lives.

**M: But you live in the same house?**

R: Yes,

**M: Are you ever curious to find out if they do or not?**

R: I am carious.

**M: You would like to know?**

R: Yes.

**M: Why don’t you ask them?**

R: They are very huge, I don’t want any issues.

**M: I hope that I don’t make you scared as well.**

R: No.

**M: So their big size intimidates you?**

R: Yes.

**M: What do you think would be their response if you asked them?**

R: I am not sure but at this point I feel like everyone will just live their lives and mind their own business.

**M: Are there situations you find it hard to cope with your HIV status?**

R: Yes, in school.

**M: What are some of the things that act as stresses for you generally in life?**

R: Relationships,

**M: What about it are you afraid of disclosing your status or courting them?**

R: Ladies can be very cunning and then you find all your friends have girlfriends and maybe you are not able to approach one due to your status.

**M: You would like to have a girlfriend?**

R: Yes,

**M: Have you tried speaking to any?**

R: Not yet.

**M: Why not?**

R: You might open up to her and she tells people, because at some point your relationship you will stop keeping secrets and you might feel the need of sharing with her but how sure are you that she will not leave you or even tell other people? Or even blame you for not telling her initially during the relationship.

**M: And that is quite stressful disclosing your status?**

R: Yes.

**M: How about courting her?**

R: That is not difficult, the main issue is disclosing my status.

**M: Do you currently have a girlfriend?**

R: Yes.

**M: Have you disclosed your status to her?**

R: Not yet.

**M: Do you feel she suspects?**

R: I am not sure but with time she will suspect me.

**M: You feel she will eventually suspect?**

R: Yes, maybe if we end up together living in the same house or spending time in the same house I might not be too careful and one time she might find out. I might remove the label from the medicine but if she is well educated she will eventually find out on her own if she is curious. I might lie to her that they are medicine for my chest but if she is a smart girl then I might not keep the secret for long.

**M: That is something you feel might stress you in the future?**

R: Yes.

**M: How do you deal with your stress?**

R: I listen to music or practice my dancing.

**M: So for you music does the magic?**

R: Yes.

**M: Any other things you do?**

R: Nothing.

**M: Now when you need emotional support whom do you rely on?**

R: I don’t trust anyone in my community.

**M: So if you need emotional support you don’t speak to anyone?**

R: No, I don’t.

**M: How do you deal with that?**

R: I record myself on the phone and then I latent o it and answer myself then delete later on because someone might come across my phone and listen to my recordings.

**M: What questions will you ask yourself and how do you answer yourself?**

R: I cannot recall, I used to do that a long time ago.

**M: Is it that you don’t want to share with me?**

R: No, that is not the case.

**M: How do you answer yourself, do you assume the characters of two people or just one character?**

R: Two people.

**M: And you will be the one with the problem and the other character responding to your questions and advising you?**

R: Yes.

**M: Who would that person be in your mind?**

R: My girlfriend.

**M: But you don’t have the courage to ask her directly?**

R: Yes.

**M: That is interesting, so you answer yourself how you feel she might respond?**

R: Yes.

**M: Whom do you talk to when you need any other kind of support not necessarily emotional whom do you rely on?**

R: When I need any other support I talk to my big sister, but she doesn’t live at home she moved out.

**M: Does she support you?**

R: Yes, I talk to her when I just need to talk to someone.

**M: Any other support you need?**

R: None.

**M: Where do you get money to buy clothes?**

R: I am in a dance group and when I dance and we get paid then I use part of that money but not always, my parents also buy me clothes but there are times I need to buy certain types of clothes, so I have to look for the money and buy those clothes.

**M: Where do you get financial support?**

R: I get my own money most of the time.

**M: So you support yourself financially?**

R: Yes.

**M: Any other kind of support you need?**

R: None.

**M: Who bought you the mobile phone?**

R: I bought it with my own money.

**M: So you are financially capable?**

R: Yes, I had to buy this particular phone that I needed on my own, if I wait for them they will not buy what I need.

**M: Have they ever bought you any phone?**

R: Yes, they bought me the first phone that I owned but it was the feature phone with buttons and I wanted a touch screen phone.

**M: When you dance you get enough money that you can even use to buy a phone?**

R: Yes, I do.

**M: When you have issues to do with HIV whom do you talk to?**

R: Then I come here to the hospital and speak to the counselor, at times I attend support groups and everyone shares their experiences and we all learnt from that.

**M: Do you attend support groups?**

R: I only attended once.

**M: When was it?**

R: I think around 2016.

**M: Do other youths attend the support groups?**

R: Yes.

**M: How do you know that people come for the support groups?**

R: Its normally held on Saturdays during the last week just before we go back to school.

**M: Do you feel like back at home you get support?**

R: Yes.

**M: How do they support you?**

R: By ensuring that I take my medication and if I skip then I will be in big trouble and they ensure that I eat a balanced diet that was recommended at the hospital.

**M: How about friends at home and in school do you feel that you are open with them?**

R: Yes,

**M: Have you disclosed your status to your friends both at school and at home?**

R: No, I haven’t.

**M: Is there any other person you wish they would be supporting you but they currently do not support you?**

R: There is none.

**M: Have you ever experienced HIV stigma?**

R: No, I haven’t.

**M: Incase you feel stigmatized what would you do to deal with that stress?**

R:If that person is my age mate then we sort it out verbally by talking if they don’t then we find other means but if it’s someone bigger than me then I will find someone to talk to them, but I don’t think that would really happen it’s very unlikely.

**M: I would like us to talk about ARV’s and how does it feel taking them?**

R: It is very challenging because you cannot just go anywhere and decide to stay overnight without planning because you have to take that medicine daily, so if you are planning to go anywhere you have to carry medicine. If you are going camping then you have to carry that medicine and it can be quite a challenge at times and so normally what I do is carry the ones that I will just need for the days that I will be away.

**M: How many days do you go for camping?**

R: A week.

**M: What do you do in that case do you carry for the whole week?**

R: If we are going for long then I carry for a week but at times I just carry a few more for emergencies.

**M: What kind of emergencies?**

R: Some might get lost or get damaged.

**M: And that might be a challenge if you don’t have extras?**

R: Yes

**M: Do you face any difficulties with taking the ARV’s?**

R: No, I don’t.

**M: I spoke to some people and they mentioned that they face many challenges when it comes to taking ARV’s? Are there times you feel like not taking your medicine?**

R: The only thing that I struggle with is drinking the medicine with water

**M: What do you prefer, what would you do if you were a manufacturer?**

R: I would have a syrup.

**M: Why?**

R: Those tablets leave a powdery after taste on the tongue.

**M: And you don’t like that feeling?**

R: Yes.

**M: What are your reasons for you to continue with your ARV’s?**

R: The fact that I am not the only one and I see even younger children than me who are already taking them so that motivates me. And then there are other worse diseases that don’t even have a prevention or solution and those people are at risk, at least we have a solution to live a long life as long as you take the medicine as prescribed.

**M: Any other reasons that make you not want to take medicine?**

R: My girlfriend I feel that I will be healthy enough to be with her, even if it’s not physical but I will be there with her. If I don’t adhere to the medication then my health might deteriorate and I die and she ends up with someone else.

**M: She is really special?**

R: Yes, she is.

**M: Do you feel like you will grow together?**

R: I am not sure about the future but I pray for that to be.

**M: Does she know that you love her that much?**

R: Yes she does.

**M: Any other thing that motivates you to take medicine?**

R: There is nothing else.

**M: Are there times you don’t feel like taking the ARV’s?**

R: Yes, when I am sick and maybe I have to take other medicine in addition to the ARV’s that stresses me.

**M: Any other times you don’t feel like taking medicine?**

R: At times I am preoccupied and I delay in taking medicine.

**M: What happens when you delay does that discourage you?**

R: Then I have to take the following day.

**M: Those are the challenges you face with the ARV’s?**

R: Yes.

**M: Any other challenges you face taking ARV’s, there is skipping, and then carrying them when you go to the camp?**

R: Yes, or at times I go for dancing practice then I come back late and that is also another challenge. At times leaving before 6:00 is hard especially if everyone is practicing they might think that I am neglecting them.

**M: You just want to hang around them and leave together?**

R: Yes, and I am supposed to take the medicine at a specific time so that is a challenge.

**M: What do you do then in that situation do you take the medicine late?**

R: Or I just fail going for practice.

**M: Because you might come late?**

R: Yes.

**M: Why not carry the medicine?**

R: That is hard.

**M: How many pills do you take in a day?**

R: Two in the morning and one in the evening.

**M: Then why not just carry one?**

R: I can but I have to drink it and relax for a while, I cannot take it during practice or if I have to move around.

**M: So that is a challenge?**

R: Yes.

**M: What should be done to make it easier for you to continue taking your drugs?**

R: I always wish that I would be open with one of my friends that we practice with so that I can just tell them about my condition and then I don’t have to hide when I need to take medicine that would have really made me so happy. But you just never know once you tell one person what they might do with that information.

**M: It would have been good if you could only tell one person that would understand what you are going through?**

R: Yes.

**M: Then you mentioned that you are not sure if anyone in your family is taking the ARV’s?**

R: No, I am not but all my siblings are negative.

**M: I want you to tell me what are your thoughts about the future?**

R: I keep worrying how I will be able to disclose my status to my future girlfriend or partner.

**M: Anything else?**

R: That is the main issue.

**M: How do you feel about joining the adult’s clinic when the time comes in the future when you turn 18 years?**

R: I have never thought about it but the youth clinic would be okay with me.

**M: But you don’t feel comfortable with the adult clinic?**

R: Yes.

**M: Any concerns about joining the adult’s clinic, what challenges do you foresee?**

R: Some of these adults can be so nosy and when you walk in everyone will be looking at you and judging you and so forth and I don’t like that.

**M: Where do you see yourself 10 years from now health wise?**

R: My viral load would have reduced.

**M: Are there goals you want to achieve in the next 10 years?**

R: Yes.

**M: What are your future goals?**

R: To get a good job and a good partner in future, not one that will give me too much stress and then on top of that I have to deal with my parents stress as well.

**M: That is a lot of stress?**

R: Yes, especially if you don’t have a good job and you look presentable then no lady will be attracted to you. And I have to support my family as well.

**M: And there is a lady expecting you to support them so you need to have a well-paying job?**

R: Yes.

**M: Any other dream apart from getting a well-paying job?**

R: Maybe travelling abroad.

**M: Do you feel like your status might affect or stop you from achieving your goals if maybe you don’t get a good job or you fail to go abroad?**

R: That will not affect me at all.

**M: Then would you like to have a family in the future?**

R: Yes, I would like to.

**M: Thinking about the family what are your future concerns?**

R: My children being infected and then they have to keep taking medicine daily and then at times they have to skip school due to their health

**M: Any other concerns?**

R: That one mainly.

**M: Why do you feel that way?**

R: When the children keep missing school then they lose a lot.

**M: And you would not want your children to miss school due to their status?**

R: Yes.

**M: Do you feel that your children will be HIV positive?**

R: No, I want them to be HIV negative.

**M: Why?**

R: That is what I hope for, some might be negative and others positive like in my family but I wish that they will all be negative like my sisters are HIV negative.

**M: When you need advice on relationships whom would you talk to? First of all do you need such information?**

R: Yes, I listen to Dr. Love on Kiss FM.

**M: Anywhere else you need advice?**

R: I don’t need any other information.

**M: There is no where you look for information in order for you to be a better boyfriend?**

R: No, I don’t.

**M: Any other place you can get information about relationships?**

R: From church.

**M: Do you go to the church to ask some questions or seek advice whenever you have issues?**

R: No, I just listen to the pastor during the sermon.

**M: You don’t approach them directly?**

R: No, I don’t.

**M: Why not?**

R: I don’t have a specific reason but that is what I prefer.

**M: As we wind up, are there areas you feel like you need support through support groups from the facility or clinic staff, or areas that you feel they should focus more on that are very important?**

R: Getting more medicine especially when I have traveled to a different facility or region because it’s entirely another longer process.

**M: It would be good if you could get a refill from any other facility?**

R: Yes, it would especially when you are not planning to go back to your original facility within a specific time and your clinic is due, I wish it was networked so that you can receive assistance from wherever you are, or if they had an option whereby someone can bring them to you.

**M: So what happens when your clinic is due you have to travel back?**

R: Yes, you have to travel back even if you were in Migori

**M: What if there was an open session so that people discuss and share information that is useful, would you be interested to attend?**

R: Yes, that would be a good idea.

**M: So you would be interested to attend those discussions?**

R: Yes, I would attend.

**M: How often should you have those sessions if we had them during holidays like April, August and December?**

R: I would prefer them during December.

**M: Which days of the week between Monday to Sunday?**

R: It should be held after Christmas.

**M: But during the week which are the best days?**

R: Tuesdays, Mondays are normally bad days so Tuesdays would be okay.

**M: Would you attend if it was held maybe on a Tuesday in April?**

R: Yes, I would attend.

**M: Any challenges you foresee that might stop you from attending?**

R: If you have it in December on Saturdays because I have places and things that I normally do so it might be difficult for me to attend.

**M: Any challenges you foresee?**

R: Distance, if I have to use bus fare then it might be a challenge for me.

**M: Any other thing?**

R: There is none.

**M: Do you have any other questions or comments?**

R: No.

**M: Okay thank you.**

R: Welcome.
