# Supplementary material for: Psychosocial needs among older perinatally infected adolescents living with HIV and transitioning to adult care in Kenya
Source: PLoS One. 2020 Jul 29;15(7):e0233451. doi: 10.1371/journal.pone.0233451 (PMC7390380; doi:10.1371/journal.pone.0233451)
Supplement: S1 File — (ZIP) [file pone.0233451.s002.zip › uploaded final PLOS/Reviewed Transcripts/IDI4M.doc]

**M: So maybe we can begin with a brief introduction, tell me your name, what you do, if you are in school and anything else you would like to add?**

R: My name is xx I study at xx High school.

**M: Which form?**

R: I will be joining form three.

**M: I would like us to discuss about your experience generally how has it been?**

R: At times when my viral load goes up I attend many seminars and that makes me feel pressured.

**M: Any other pressures?**

R: That is the main one.

**M: How long have you been coming here?**

R: Since 2007

**M: And before 2007 where did you go?**

R: xx.

**M: Where is it?**

R: Next to the xx.

**M: Along xxx road?**

R: Yes.

**M: Do you now understand the process when you come to the clinic and what happens?**

R: Yes, I understand the process i have been coming here for many years.

**M: Could you take me through the process, what happens when you get to the facility?**

R: I have to go for testing of my blood pressure first and then I get tested and then a brief meeting and then I get to see the doctor, then if there is any blood that has to be tested then the blood test is done and then I book the next appointment and then I take medicine and go back home.

**M: Do you attend meetings?**

R: Yes.

**M: What are those meetings for, you said part of the process is attending meetings?**

R: They are not really meetings but counseling sessions.

**M: You go for counseling?**

R: Yes, there was another seminar I attended when I turned 17.

**M: Was it here at the hospital?**

R: Yes, it was.

**M: Could you take me through the experience with the counselor the first time when you knew about your HIV status, how was it? How did you know about your status?**

R: My aunt told me.

**M: What triggered her to tell you and how was the whole process?**

R: I had just come from one church one Sunday and she called me to her bedroom and told me about my status.

**M: How did she begin, I want to feel like I was there during that time?**

R: It was just like a story because she told me about when my parents God married and I was conceived but during birth I contracted HIV.

**M: How did you feel at that time?**

R: When she told me I didn’t feel anything but a day later it really weighed on me and I started questioning if that meant that I will not marry and have a family.

**M: You started questioning if you will get married?**

R: Yes,

**M: Any other questions you had?**

R: If that meant that I was going to die soon?

**M: Any other questions you had?**

R: If everyone was going to know that now I was suffering from HIV.

**M: Any other questions you had?**

R: And how my family will react and treat me because I was suffering from HIV.

**M: You currently live with your aunt?**

R: Yes.

**M: How about your parents?**

R: My mum died and my dad is in the states.

**M: By family you meant how your cousins were going to respond?**

R: Yes.

**M: Do they know about your status?**

R: Yes, they do.

**M: Have you ever discussed with anyone about these questions or you just have them at heart?**

R: I just have them at heart.

**M: Have you ever received any responses to these questions in any way?**

R: I will only ask the doctor when I am mature and ready to get married.

**M: You don’t want to ask now?**

R: Not yet, I don’t think it’s necessary to get that information now.

**M: You would rather keep it at heart till you plan to get married?**

R: Yes.

**M: When do you plan to get married?**

R: In 10 years.

**M: You will keep those questions in mind for 10 years?**

R: I will probably ask my cousin when I complete high school.

**M: You are free with your cousin?**

R: Yes, I am.

**M: Do you feel your cousin will have that information?**

R: Yes,

**M: How was your experience the first time you spoke to the counselor about your HIV status?**

R: I was young but I remember her telling my aunt to take good care of me, and she should ensure that I eat well and she reminds me to take my medication at a specific time, and she gave my aunt a list indicating the meals she should prepare for me.

**M: How did you feel about that?**

R: I felt pressured and I realized that she was really treating me well and I didn’t want to be treated “too good” like I am too delicate because I had HIV. I just wanted to be treated normally.

**M: Being treated too good because you are HIV positive keeps you pressured?**

R: Yes.

**M: Were you treated differently at home because you were HIV positive?**

R: No, I was being treated normally.

**M: But you felt like the counselor wanted you to be treated differently?**

R: Yes,

**M: Did you speak to your aunt about that, about the treatment?**

R: Yes, I asked her to treat me normally.

**M: What did she say?**

R: She was okay about it.

**M: Okay, and did you ask the counselor any questions at that time?**

R: I cannot remember the questions, but I asked her some questions.

**M: Can you remember even one questions?**

R: No, I cannot remember the questions.

**M: So, the time maybe you knew about your HIV status, was there anything you wished would have been addressed that day?**

R: We were taught in school that while in the uterus your blood cannot mix with your mums unless there is an accident and in my case I contracted the HIV virus as a result of an accident and I keep wishing that accident would not have occurred because my brother is not HIV positive.

**M: That is one thing you wished for?**

R: Yes.

**M: And how did that make you feel?**

R: It really made me feel bad.

**M: It made you feel bad?**

R: Yes.

**M: How have you been copying emotionally since that time you knew about your HIV status?**

R: At times I feel sad and at times I feel happy.

**M: What triggers that sadness?**

R: There was a day when some visitors came to my aunties house and during the introduction she introduced everyone and forgot to introduce me and I remember that day I felt really sad, I just went to the bedroom and locked myself in the bedroom and I kept asking myself why she forgot about me. And I started feeling sad that maybe she didn’t introduce me because I was HIV positive.

**M: Did you walk out?**

R: Yes, it was during the day and I went to my bedroom and slept till the next day.

**M: Nobody came to check on you?**

R: No, the next day my aunt came to the bedroom and asked where I had been and I told her I was in the bedroom.

**M: Did she come to ask why you were sitting alone in the bedroom?**

R: No, she didn’t and she has never asked me again.

**M: Have you ever discussed with anyone about that incident?**

R: No, I haven’t.

**M: This is the first time you have talked about it?**

R: Yes.

**M: How do you feel about it, now that you have talked about it?**

R: I feel bad.

**M: Has something similar happened to you and it made you feel bad?**

R: Its mainly during the introductions, when visitors come home she keeps forgetting to introduce me most of the times.

**M: How does that make you feel?**

R: Like I am not part of the family because I am HIV positive.

**M: You don’t feel she does that because you are not the biological son?**

R: That could be another reason.

**M: How do you feel when that happens and what do you do?**

R: I just sleep.

**M: What questions run through your head or what do you wish for before sleeping?**

R: I just want to work hard in school and then later on in life, I would like to have my own family.

**M: Do you speak to your dad?**

R: Yes.

**M: How often?**

R: Once a while during holidays.

**M: Because you are in boarding school?**

R: Yes.

**M: How do you feel that you are not in constant communication with your father?**

R: Nothing, I got used to that life.

**M: When did she travel to the states?**

R: Long time ago, when I was in class 7.

**M: You don’t wish to join him?**

R: He told me that I will join him later but I will be visiting him this year August. I will be processing my passport later on today before going to school.

**M: And you said that when you feel bad you go to bed and when you sleep that makes you feel good?**

R: Yes, but at times I feel nauseas and really hungry and when I eat I don’t get satisfied.

**M: You eat and you still feel hungry?**

R: Yes, then I feel dizzy.

**M: Does that happen all the time or when you are stressed?**

R: When I am stressed.

**M: What helps you cope with your status?**

R: My future dreams motivate me.

**M: What are some of your aspirations?**

R: I want to be a journalist or a doctor.

**M: And you are working hard in school?**

R: Yes.

**M: When did your aunt reveal your status to you, how old were you?**

R: 12 years.

**M: If you compare now that you have knowledge about your status to before when you didn’t know about your status which one is better?**

R: Now that I am aware of my status.

**M: Why?**

R: Before I knew about my status I would just swallow medicine but I didn’t understand why I kept on swallowing medicine and I never recovered but my aunt kept telling me that I was taking medicine because I was sick and at times I would miss because I didn’t realize the importance. But now that I am aware of the importance I don’t miss because my life relies on it.

**M: You kept wondering why your aunt told you were sick and you were not recovering?**

R: Yes.

**M: Why did you used to miss medicine?**

R: I didn’t understand the value, because I didn’t feel sick and I didn’t see the benefit of that medicine. My aunt kept telling me that I was sick but I didn’t feel sick, so I would ignore intentionally.

**M: And is that aunt your mothers or father sister?**

R: My father’s sister.

**M: In your life are you aware of anyone dealing with the same things that you are dealing with currently, someone who influences or encourages you?**

R: No.

**M: What are some of the things that act as stresses for you generally?**

R: At times when my grades drop in school that stresses me up.

**M: Any other times in life not necessarily about academics?**

R: After counseling due to my viral load going high.

**M: What about counseling should be done differently to reduce that pressure?**

R: The counseling should be interactive the way we are talking with your right now.

**M: The counseling should be interactive?**

R: Yes.

**M: How is it normally done?**

R: You just sit down there and listen to them talking and talking and you have no say.

**M: So it should be conversational?**

R: Yes.

**M: Do you feel that if they do that, the pressure will reduce?**

R: Yes.

**M: And academically when your grades go down how do you cope with that stress?**

R: I have to study hard during the holidays and also taking walks to reduce the stress.

**M: Any other thing you do to reduce stress?**

R: Singing and going for church retreats.

**M: When the viral load goes up after counseling, how do you cope with that stress?**

R: I just have to be keen and take my medicine as I should.

**M: What causes your viral load to go up?**

R: When I am at home it goes down because I take my medicine well but at times in school I don’t there are a lot of interruptions, there are school trips and activities that at times we have to attend very early and at times I end up not taking my medication.

**M: So at times when in school you miss taking the medication?**

R: Yes.

**M: Now when you need emotional support whom do you rely on?**

R: It was my cousin, but she got married.

**M: So you cannot talk to her because she got married?**

R: Not as much, not unless I go for a sleep over at her place

**M: When did she get married?**

R: May 2018.

**M: Any other person apart from your cousin?**

R: She is the only one.

**M: What if you need any other kind of support whom do you rely on?**

R: If its financial then I talk to my aunt and she calls my dad.

**M: When you have issues to do with HIV whom do you talk to comfortably?**

R: There is no one.

**M: What do you do then?**

R: I wait till my appointment date and speak to my doctor.

**M: Do you feel like your family is supporting you?**

R: The only supportive people in my family are my aunt and my cousin and the rest are just normal. They know I suffer from HIV but they treat me normally but my cousin really supports me.

**M: Your cousin who got married and your aunt?**

R: Yes.

**M: How do they support you?**

R: They generally encourage me and remind me to take my medication.

**M: How about friends?**

R: I have not told any friend about my HIV status.

**M: You are free with your friends but you are not free to share with them your HIV status?**

R: Yes.

**M: Why not?**

R: I feel that they might start behaving differently around me and I don’t want that.

**M: What is it about your friends that makes you feel that if they know your HIV status they will behave differently about you?**

R: I am not quite sure but I don’t just want to share with them my status.

**M: Is there anyone you wish they would support you more but they don’t support you more?**

R: My aunties family members.

**M: Why don’t they support you?**

R: Most of them are busy, they even forget that I take medicine. Like after a long time if he sees me taking medicine he would be like , oh, so you are still taking those medicines?

**M: And you live in the same house?**

R: Yes, we do and then it’s not like I take my medicine openly, I just take them in the bedroom.

**M: Do you feel free to speak to them freely about your HIV status?**

R: No.

**M: What makes it difficult to speak to them about your HIV status?**

R: They don’t take it seriously.

**M: Have you ever experienced HIV stigma due to your status by anyone?**

R: Yes, I have like during those introductions and I am left out of the introductions, but I would not attribute that 100% to my status because of my HIV status, so I would not describe it as stigma.

**M: Any other time you have experienced stigma apart from that time?**

R: No.

**M: How has your experience been using ARV’s you had briefly mentioned how you used to skip when started taking them because you didn’t understand the benefits or you would forget taking them especially when you went out for funkies?**

R: Yes.

**M: Any other experiences using ARV’s?**

R: The side effects at times while in class , the main ones are nausea and dizziness, at times I feel sleepy and at times some teachers send me out of the class, because I am sleeping and it’s because I have not told them about my status.

**M: That was my next question nobody knows about your status in school?**

R: The deputy principal knows because I report to him most of the time.

**M: Why do you report to him?**

R: When I have been sent out of class and he talks to the teacher and I go back to class.

**M: Do you find it helpful when there is someone in school who knows about your status?**

R: Yes, I find it helpful.

**M: Would it be better if more people knew about your HIV status?**

R: I don’t think that would be helpful because there are some people who might tell other students and that might cause more stigma to me so I cannot share with everyone.

**M: The deputy principal has dealt well with it?**

R: Yes.

**M: Are you aware if there are other students who are HIV positive in your school?**

R: Yes, the deputy principal told me that I am not alone and there are other students who are HIV positive but he didn’t tell me their identities which is good.

**M: Would you like to know who these students are?**

R: No, he has kept my identity private, so let him also keep the identity of the other students privately.

**M: Don’t you think it would be good if you knew at least one person who was HIV positive so that you can openly and freely share about your HIV status?**

R: It would have been good but the deputy principal wouldn’t tell me who they are maybe because they don’t want to be known, but when I go back to school I might try asking again.

**M: What has motivated you to continue with your ARV’s?**

R: I have dreams that I need to achieve.

**M: Are there times you feel like not taking your medicine?**

R: Yes, when I have not performed well so that my aunty forgets about my results and focuses on my health.

**M: Do you just skip the medicine or it crossed my mind?**

R: It is hard to ignore because my life depends on it but I have thought of that so that my aunt doesn’t lecture me about my academic grades.

**M: Is your aunt harsh and does she get angry at you when you don’t perform well in school?**

R: She used to be very harsh when I was in primary but she has relaxed and is not very harsh since I joined high school.

**M: She doesn’t ask you when you drop in grades?**

R: She tells me to work hard and improve on the grades and encourages me but I still fear her due to the fear she instilled in me since I was in primary, that fear hasn’t gone away and I fear she might get me off guard at one point.

**M: What should be done to make it easier for you to continue taking your drugs?**

R: There are those rotators that I had in primary school and they used to help me remember taking my medicine but I lost them when I had gone for a retreat for two weeks and those rotators used to help me remember when it was time to take medicine. It was fun because I would open them for instance and see that today is Tuesday, and it would remind me when I needed to take the medication. And it would even help me remember the days I had not taken the medicine.

**M: They are called mem scarps?**

R: Yes, if there are some in this hospital you can help me with a few.

**M: I will ask, I am not sure if they have them but they were helpful to you?**

R: Yes and fun.

**M: That opening and being able to know the days of the week like today is Monday, Tuesday?**

R: Yes.

**M: Anything else that would make life better and easier while taking your medication?**

R: There is nothing else.

**M: Do you have someone else maybe a friend, family member or in your home who takes the medicine?**

R: Yes, my dad.

**M: In your opinion what motivates him to continue taking the medication?**

R: I am not sure, since he is not around.

**M: And you mentioned that you think about the future goals, family?**

R: Yes.

**M: Have you ever thought about joining the adult clinic once you clear high school?**

R: I have always known that there is just one clinic, I didn’t know that there is an adults clinic and a children’s clinic. I would just come to the clinic and then receive counseling and go home.

**M: You never knew there is an adult and adolescent clinic?**

R: Yes, so it has never crossed my mind.

**M: Where do you see yourself 10 years from now health wise?**

R: My health will have improved, if I continue taking medicine.

**M: Are there goals you want to achieve in the next 10 years?**

R: I want to be a doctor and journalist.

**M: Do you feel like your status might affect or stop you from achieving your goals?**

R: Not really, maybe slightly.

**M: Why, what might affect you?**

R: The side effects, though they are rare and mostly come while I am in school but that might stop me if they recur.

**M: Do you know any prominent, successful person who is HIV positive?**

R: Yes, I do.

**M: Who is that person?**

R: I know Tony Upton and Nelson on TV and they are progressing well.

**M: Then you mentioned that you would like to have a family in the future?**

R: Yes, I would like to but I am not sure I will have, because at that point I might have to disclose my HIV status to my partner and I don’t know how that might evolve from that moment henceforth. I feel that obviously she might refuse my proposal.

**M: Obviously?**

R: Yes, because she might not want to be infected with the HIV virus.

**M: So you feel like you might never have a family?**

R: I will try but if she declines my offer then I will let it go.

**M: The main obstacle for you is the disclosure, disclosing your status to your girlfriend or wife to be?**

R: Yes.

**M: Any other future obstacles?**

R: That is the main one.

**M: Why do you feel that your wife to be might not accept your marriage proposal once she knows your status?**

R: I am sure there is so much information out there and we heard a lot about it, in primary as well during those poems like “ukimi inaua” and even in high school and other sources and so some people don’t understand it well and some people feel that it’s very dangerous and so obviously she might refuse because she doesn’t want to contract that virus.

**M: Because they have been taught that?**

R: Yes, that is what they know that it’s a deadly disease.

**M: So there is a lot of misinformation at primary level?**

R: Yes, especially with those poems like the one I mentioned above.

**M: If you were a teacher how would you do it, if you had to pass the message?**

R: I would not portray it as a deadly diseases because that increases the stigma. There is a difference between ukimwi and HIV and I would explain to them.

**M: The way that message is currently portrayed, how does that affect the children who are already HIV positive? For instance if that poem of “ukimi inaua” and there are children who are already HIV positive in that class maybe two or three how do they feel when that poem “ukimi inaua” is narrated?**

R: They feel bad.

**M: So you feel that those girls have already been warned since they were in primary and so when it comes to marriage and you disclose your status they will not accept to get married to you?**

R: No, they will not but I will try.

**M: Do you currently have a girlfriend?**

R: I can have a girlfriend right now if I want to because she wouldn’t know my status but I don’t have.

**M: If you have a girlfriend you will not tell them about your status?**

R: No, I will not.

**M: Why not?**

R: If I disclose my status, she will start acting differently. It’s just the same way I would not tell my friends about my status because I feel like they will start acting differently.

**M: So if you have a girlfriend you will just keep it from them?**

R: Yes.

**M: Will you engage in sex?**

R: I will not engage in sex, not only due to my HIV status but also because of what we have learnt in church, that we should avoid fornication.

**M: Do you feel like you need any information or advise on how to deal with certain issues in relationships?**

R: Yes, issues regarding girlfriends and the relationships with girls.

**M: Any other information?**

R: (Silence)

**M: When you need advice on relationships whom would you talk to?**

R: A friend.

**M: Any other place you can get information about relationships?**

R: Mostly friends and if it’s about a girlfriend then I would ask my girlfriends friend.

**M: Have you found it useful being able to speak to your friends about relationships?**

R: Yes, it’s useful.

**M: As we wind up, are there areas you feel like you need support through support groups from the facility or clinic staff, areas that you feel they should focus more on that are very important?**

R: About relationships and the family, if you can still get married even when you have HIV.

**M: Do you attend youth support groups here at the hospital?**

R: I have never attended; I only attended one in 2017 when I was in form one.

**M: What information do you feel is important to receive?**

R: It was mainly repetitive but they focused mainly on adhering and STI’s, which I already know about but they kept repeating the same information.

**M: Anything you feel you need to know more about to be able to stay healthy, now for you as an individual?**

R: I already know the information that I need.

**M: You are busy in church and that might stop you from coming?**

R: Yes.

**M: Okay thank you.**

R: Welcome.
