# Supplementary material for: Psychosocial needs among older perinatally infected adolescents living with HIV and transitioning to adult care in Kenya
Source: PLoS One. 2020 Jul 29;15(7):e0233451. doi: 10.1371/journal.pone.0233451 (PMC7390380; doi:10.1371/journal.pone.0233451)
Supplement: S1 File — (ZIP) [file pone.0233451.s002.zip › uploaded final PLOS/Reviewed Transcripts/IDI6B.doc]

**M: We can begin with the introductions and then you can tell me what you do during your free time?**

R: My name is XX I enjoy playing football, PS during my free time.

**M: I would like us to discuss about your experience in this clinic, how was it?**

R: It was good, everyone is friendly and they handled me well.

**M: How long have you been coming here?**

R: 2019 will be my second year.

**M: Before 2019 where did you used to go?**

R: XXX

**M: And then you were transferred here?**

R: Yes.

**M: When you say that everyone is good, what does that mean?**

R: They treat you well, no discrimination, and they don’t discourage you in any way.

**M: What else do you like about this facility?**

R: I have seen my age mates and even some people younger than me.

**M: And that helps you a lot?**

R: Yes, it does.

**M: Anything else?**

R: There is nothing else.

**M: And currently you come along to the clinic?**

R: Yes, I do come alone and if my appointments fall on the same date as my mum then we come together.

**M: At times your appointments fall on the same dates?**

R: Yes.

**M: But does she accompany you when your appointments are not on the same dates?**

R: No, only when she is coming for her appointments.

**M: meaning you understand the whole process that goes on here at the facility when you come?**

R: Yes, I do.

**M: Could you take me through the process, what happens when you get to the facility?**

R: I have to give out my card and they ask me several questions, challenges, or if I was affected by any diseases, then my weight is measured and I go to the doctor.

**M: What happens after seeing the doctor?**

R: I take my medication then I go home.

**M: So, when was the first time you spoke to a counsellor about your HIV status?**

R: 2016.

**M: Could you take me through the experience, how was it?**

R: It was very difficult because I knew about my HIV status that I was HIV positive when I was in form one as I became really sickly towards the end of third term and I was very sick for about a month in November and then my mum took me to Mama Lucy Hospital. And then I was tested and it was confirmed that I was HIV positive.

**M: So you had not been tested throughout?**

R: No, I hadn’t.

**M: What was ailing you?**

R: I just felt sick and I would vomit a lot and I had headaches.

**M: How did you feel when your HIV status was confirmed to you?**

R: I was really hurt because I was told that I had it since I was born but I only got to know about it while in form one and yet I would have started treatment earlier. Because even in church I saw some members who were infected and they were already on medication and they were just living a normal life.

**M: You saw some people and you couldn’t believe that they were infected and you could also be one of them?**

R: Yes,

**M: Did you have any questions in mind?**

R: Yes.

**M: What questions did you have in mind?**

R: I didn’t want to judge her as my parent at that time.

**M: Any other questions because of course when such news is delivered to you, of course you have to have some questions when you are alone somewhere?**

R: I remember thinking that there were times when I was younger I would go to my mum’s wardrobe and there was a bag containing many tablets but when I asked her she lied to me about what she was suffering from, and I never bothered to follow up but I was still questioning what she was suffering from that made her to have so many drugs. But later on in life she confirmed to me that she was HIV positive as well as she could not hide it from me.

**M: Do you have any other siblings?**

R: Yes, older than me because I am the youngest.

**M: What is their HIV status?**

R: They are negative.

**M: And once you understood her status you knew the reason why she had the medicine?**

R: Yes, I did.

**M: What questions did you have in your head at that point?**

R: I was just wondering why she didn’t tell me about my status earlier on.

**M: Was she aware of your condition at that point?**

R: I am not sure if she was aware, but again she could have known because she told me that I was born HIV positive.

**M: But you had never been sickly before?**

R: Not for one month straight on.

**M: You were sick for an entire month?**

R: Yes.

**M: Were you admitted?**

R: No, but I was just in the house.

**M: Did you ask any questions to the counsellor?**

R: Yes, I did especially how to deal with the curious school mates because when they ask me I tell them that they are medicine for my chest and they keep asking me why I never recover from the chest infection so that is wearing me down.

**M: What questions did you ask for the counsellor?**

R: How I should go with those drugs to school because when we get to school we are searched and I cannot hide those medicines and so I keep telling them it’s for my chest but I have been taking those drugs since form one.

**M: Whom have you been telling that to?**

R: The prefects because they are the ones who check us when we report to school.

**M: Do you feel like they don’t trust you?**

R: Yes, they might go and find out more about my drugs if I leave them in the packet and that is why I remove them from the original package and put them in another paper, but my concern is if they take one tablet and go research about it and then they tell other people in the school.

**M: Do you feel like maybe they know about your status?**

R: I feel that they don’t know because I keep telling them that those drugs are for my chest. And it’s even easier because I have another friend who had a chest infection and constantly takes drugs so that helps me a lot as well.

**M: Any other questions you had to the counsellor?**

R: (Silence)

**M: Where was your status disclosed was it at the hospital or through your mum?**

R: I had gone to the hospital with my mum and the doctor talked to both me and my mum and my mum explained further to me.

**M: The first information about your status was from the doctor and then your mum explained it further to you?**

R: Yes.

**M: That was at the hospital?**

R: Yes.

**M: How was your journey back home, did you talk to her or were you very upset with her?**

R: I was very upset with her at first and then later on I decided to take control of my life and health because I am the victim here and life has to go on.

**M: Did she figure out that you were upset with her?**

R: Yes, she did and she always knows when I am upset with her.

**M: Did she make any efforts to console you?**

R: Yes, and later on I just took it as normal because she told me that she was infected when she was 22 years old.

**M: How have you been copying emotionally since that time?**

R: I have been trying to treat it as normal. I don’t want to judge myself or my mum but I just take it as a normal thing and I don’t want to feel like since I am HIV positive then I cannot do whatever I want to do in life and live a normal life. I interact with other people well.

**M: Are there times you feel down emotionally?**

R: No, I don’t.

**M: What helps you cope with your status?**

R: My parents, she makes me feel that life has to go on. And even my dad was born HIV positive and he lived for long with it so I feel that if I take my medication diligently then I can equally live a long life because he is still alive and he will be turning 48 years soon.

**M: How about your siblings?**

R: I told my sister because we are very close and then the other one is already married so we are not very close.

**M: How did she react when you told her?**

R: She took everything as normal and she treats me very normal nothing is weird within us.

**M: If you compare now that you have knowledge about your status to before when you didn’t know about your status which one is better?**

R: I would say now because I have learnt a lot of things and I have attended lots of youth’s meetings and I have vast knowledge of my status and what I should do. And I was even encouraged because there are people who are in worse situations than mine so I decided to keep living a normal life.

**M: And you felt encouraged?**

R: Yes,

**M: So you prefer now that u have knowledge about your status to before when you didn’t know about your status?**

R: Yes.

**M: And you get encouraged because you have seen your mum and dad dealing with it?**

R: Yes

**M: What do they do that encourages you?**

R: Taking their medicine they take it diligently and on time and I have followed the same routine and I am also taking my medicine at a specific time even though I am in school.

**M: Have they influenced you?**

R: Yes, and now I feel that taking the medicine is part of my routine and every day at a specific time I have to take that medicine and if I fail to take then I feel like something is just not right about my routine.

**M: I would like us to talk about stress and what stresses you generally in life?**

R: I would say the nagging from my dad when I do something wrong. I would rather someone punishes me or beats me rather than the nagging.

**M: Why does that stress you?**

R: I don’t know but that really stresses me I would rather someone beats me and we end that issue once and for all and not the constant reminder of the wrong things that I did.

**M: What do you do to relieve your stress?**

R: I get out of the house to somewhere I can be alone, or hang out with friends or even take my phone and go chat with my friends but basically I remove myself from that scene that was stressful to me and then I go back home later.

**M: Do they look for you, maybe call you?**

R: No, they don’t because I always go back after some time when I have cooled down. I even changed my number nobody knows it now because at times I don’t want anyone calling me.

**M: What if anything happens to you?**

R: Then I will call them but I don’t want them to call me.

**M: What about your sister?**

R: She has it and they can get me through my sister.

**M: When you walk out of the house you want to be left alone?**

R: Yes.

**M: Other ways you relieve stress?**

R: By playing PS.

**M: Whom do you rely on for emotional support?**

R: My mum.

**M: Why?**

R: She is always ready to talk to me and she is more available than my dad because he travels a lot.

**M: What if you need any other kind of support whom do you rely on?**

R: My sister, she is the only other family member who knows my status.

**M: Would that be support related to your status?**

R: I talk to my mum more when I need support about my status and then my sister comes second in that order.

**M: How about financial support?**

R: Then I call my dad and he sends me money.

**M: What if you need to visit friends whom do you talk to? And whom do you talk to so that they can give you permission to go wherever you need to go?**

R: I talk to my dad or my mum but normally I prefer informing them in advance, but mostly my mum.

**M: Have you ever asked for permission to go anywhere and they denied you?**

R: They have never denied me that permission since I joined form one.

**M: How supportive is your family?**

R: We are very close and any time I need support they always support me.

**M: How do they support you?**

R: They encourage me about my future plans and also when I see that my dad was born HIV positive and he is still alive at due to the drugs that makes me feel that if I continue taking my drugs then I have a chance to live a long healthy life.

**M: Are you open to any friends at home or in school about your status?**

R: I would say one in school because I support he is also taking the same drugs but there is none at home.

**M: Why do you suspect him?**

R: He takes his medicine at a specific time and he takes them daily, we are in the same cube and I see him taking medicine daily at a specific time.

**M: You told him your status but did he tell you his status?**

R: Not immediately but later on when we became close he opened up to me because initially he thought that I was tricking him so that he could open up about his status. But later on he confided in me.

**M: How did it feel confiding in someone who was not a family member?**

R: It was a bit nerve racking at that moment because I didn’t know how he would respond to me.

**M: What made you open up to him?**

R: We have been cube mates since form one and his routine and medication was almost similar to mine so I suspected we were in the same boat and so I just wanted someone to talk to.

**M: How did he reacts when you told him?**

R: He didn’t think that anyone would figure it out but I had been monitoring him for a while and later on we talked.

**M: At least you have someone you can talk to at school?**

R: Yes.

**M: Did that makes you even closer in school?**

R: Yes, we were friends even before but became closer.

**M: How about at home have you told any friends?**

R: I have not told any friends.

**M: Do you wish there was someone who would have supported you but they currently are not supporting you?**

R: I am good. I have my sister and she really supports me at home and then I have that friend in school and then my parents.

**M: How about teachers?**

R: I wouldn’t even want then to know, they will not keep my secret and I suspect they might discuss with all the other teachers, and look at me in a certain way in class and I don’t want my business out in public.

**M: Do you have any examples of why you feel that way?**

R: Yes, there is one student who is asthmatic and he shared that with the teacher and the teacher treats him like he is very delicate and I don’t want to be treated that way. When washing dormitories, you find that he is not allowed to clean and so everyone feel that he is very delicate.

**M: Because he uses an inhaler?**

R: Yes.

**M: If you are treated differently by the teachers the other students will be suspicious?**

R: Yes.

**M: Have you ever experienced stigma due to your status?**

R: No, I haven’t.

**M: What should those who have experienced stigma deal with it in order to cope with the stigma due to their status?**

R: They should be strong; like what I did. The first time I was told that I was HIV positive I took it head on because I knew that I wanted to live. And we had already been taught about it in school and I always used to ponder what I would do if infected. But once I confirmed that I was HIV positive then I had to either life or give up. So I would urge them to take care of themselves and avoid the negativity of what the other people say, this is normal and many people are HIV positive and their life still goes on normally.

**M: How has your experience been using ARV’s?**

R: By the way I feel like my appetite has increased than before, I used to eat few times but nowadays I want to eat up to four times in a day.

**M: What then do you do while in school?**

R: It becomes really tricky, but I try to carry enough snacks that I can top up with while in school

**M: So it has increased your appetite?**

R: Yes.

**M: Any other experiences using ARV’s?**

R: At times I would feel really weak before taking the medication and now that I am dutifully taking the medicine I have become stronger.

**M: Any other experiences using ARV’s?**

R: I feel that I can relate with other people and treat myself just like the other people. Honestly speaking I have never had a reason to feel sorry for myself and not enjoy living my life.

**M: Have you faced any difficulty maybe in taking the ARV’s?**

R: No, it’s not hard, I take them while in school at a specific time and at home my sister knows about my condition so I don’t feel uncomfortable taking them in her presence.

**M: There is no challenge you face?**

R: No, there is no challenge.

**M: What has motivated you to continue with your medication?**

R: The fact that I need to continue living and I am aware that if I don’t take my medication then my health will deteriorate and I hate being sick so that motivates me.

**M: Are there times you feel like not taking your medicine?**

R: No,

**M: Not even once?**

R: Maybe at the beginning it was slightly difficult and I just didn’t want to take medicine but my mum kept reminding me to take medicine and now I am used to and I don’t need anyone to remind me.

**M: What made you feel that way?**

R: The constant interruption of my program in school whereby I had to leave what I was doing and go to the dormitory to take the medicine.

**M: Any other reasons?**

R: At times we have inter school competitions and people are having fun and I have to seclude myself to go and take the medicine and then at times the dormitory is closed and you cannot access that medicine and take it in private.

**M: What should be done to make it easier?**

R: I was advised that I should walk with the medicine that I need to take in the evening for instance and then for the morning I take them early morning before going for the morning preps

**M: So learning that trick of taking the medicine with you so that it’s easier to take it at that specific time?**

R: Yes,

**M: Any other thing that can make it easier for you?**

R: Its only that.

**M: In your opinion how have your parents managed to take their medication, what has helped them to consistently take their medication?**

R: I think it’s just the commitment because she takes every day at 9:00p.m as the news begins, so that commitment.

**M: The news helps her, what if there are no lights?**

R: She checks the time on her phone.

**M: Any other thing that has helped her manage?**

R: It’s just that.

**M: What about your dad?**

R: The phone and his watch he uses either of the two.

**M: How about you, do you need reminders?**

R: I needed to at the beginning because naturally I am not someone who really likes taking medicine but I have now adjusted and I don’t need any reminders.

**M: And thinking about the future now that you are about to clear high school how do you feel about joining the adult’s clinic?**

R: I feel that privilege now that I am mature and age is coming up and now I will soon be with adults.

**M: Is it something you look forward to?**

R: Yes.

**M: Any concerns about joining the adult’s clinic?**

R: The questions, like the questions on the viral load and what you need to do to reduce your viral load, and then you might be required to motivate other people yet you have not even disclosed your status to many people so that becomes a challenge. I remember being asked if I can motivate other youths and I was like what do I even tell them?

**M: How old was that person you were to motivate?**

R: He was an adult, in college and if I compare myself to that person I feel that the person has more experience with this status than myself.

**M: Where do you see yourself 10 years from now health wise?**

R: My health will have improved, because it has continued to improve, my weight has improved.

**M: Are there goals you want to achieve in the next 10 years?**

R: I want to be a prominent person in the society, a photographer so working with the camera and being a successful Government employee as well.

**M: Do you feel like your status might affect or stop you from achieving your goals?**

R: I don’t think so.

**M: Do you know any prominent, successful person who is HIV positive?**

R: Yes, I would say my dad. I was surprised to find out that my dad was HIV positive because he doesn’t even show it by looking at him. Even my mum I was suspicious because I saw the medicine in her bedroom otherwise I would not even have known her condition.

**M: That is how you knew about your mum?**

R: Yes, but I couldn’t believe my dad because he was just living his life and he has achieved a lot in his life despite his status.

**M: Would you like to have a family in the future?**

R: Yes, a wife and children.

**M: When you think about the future having a family, a wife and kids do you have any fears, I know we all have fears in future, so what is your fear?**

R: Having to tell them how I got infected, like I got infected because their grandmother now my mother was HIV infected, as a result I was infected and probably they might also be infected because of that, that really scares me. I see many people that I wish to open up to them but I am afraid of doing that.

**M: Do you feel that your children will be HIV positive?**

R: I don’t know what can be done to stop them from being HIV positive.

**M: That will be a challenge telling your children about their HIV status?**

R: Yes.

**M: Do you see a possibility of having HIV negative children?**

R: Yes, that is possible.

**M: Are you feeling tired?**

R: Yes,

**M: I am almost done.**

R: Okay.

**M: I would like us to discuss about information or advise and what type of information would you like to receive regarding relationships?**

R: I would like to receive information on prevention so that I can avoid spreading it to other people and then I also fear telling my mum that I have a girlfriend because she will start telling me about safety and all that.

**M: You fear telling her that you have a girlfriend?**

R: I think she knows about one but I don’t know how she would feel if she knew that I had other girlfriends and what I am doing to protect them.

**M: You have several girlfriends?**

R: Yes, I do.

**M: What do you do to prevent infection?**

R: There is nothing I do.

**M: Are you engaging in sex?**

R: We are not engaging in sex yet.

**M: Do you feel like you would openly tell your girlfriend about your status?**

R: I am not sure because I don’t know how she would react. I would only confide in her if I see it becoming a serious committed relationship that might lead to marriage

**M: What do you fear she would do if you tell her at this stage?**

R: She might tell her girlfriends and I don’t know whom they might tell.

**M: So you prefer keeping it to yourself?**

R: Yes.

**M: What information would you like to receive that will protect your partner?**

R: How I can prevent them from contacting the virus.

**M: That is the main information you need?**

R: Yes.

**M: If you wanted to take your relationship to another level where would you seek that information?**

R: I would speak to someone who is more informed and get the facts right before taking to her because if I just go there unprepared then that might affect or even cause our relationship to

**M: Where do you seek for information about anything or advise?**

R: If I need information I go to my girlfriend’s best friend and befriend her so that she can tell me more about her.

**M: How about for yourself where do you get information or advise on anything?**

R: I get it from my mum as I feel confident with her and she is the one who disclosed my status to me and has continued to support me.

**M: Has the information she has been sharing with you bene useful?**

R: Yes, it has bene useful.

**M: As we wind up, there are support groups, but are there areas you feel like you need support through support groups from the facility or clinic staff, that you feel they should focus more on a certain kind of information that will really benefit you?**

R: I always deal with this lady called xxx and if I have any issues then I would go to her and speak to her comfortably and maybe she can help us discuss about the issue when we meet as youth groups.

**M: So that is what you do, you talk to her as individuals then you get together and discuss as youth groups?**

R: Yes.

**M: What information or topics would you like her to focus more on?**

R: How to prevent re-infection.

**M: What if there was an open session so that people discuss and share information that is useful, would you be interested to attend?**

R: Yes, I would be interested and my mum has been encouraging me a lot to join such sessions, and she is even the one who told me about this meeting yesterday.

**M: There will be such sessions during the holidays but what challenges do you think would stop you from attending such sessions?**

R: Tuition or maybe when I have to remain in school for a given period because now that I am joining form four I might be required to stay an extra two weeks and therefore my holiday will be shorter.

**M: Any other challenges you think would stop you from attending such sessions?**

R: That would be the main challenge because when I am at home then nothing would stop me from attending such sessions.

**M: How about time, and what day is suitable to have such sessions, how often should you have those sessions?**

R: At least four times a month during holidays that would be ideal.

**M: What day of the week would be the best?**

R: Weekends because weekdays at times I go to town to have some fun, meet up some friends so it would be difficult.

**M: You do go to town to have fun?**

R: Yes, I do.

**M: Is there anything else you would like to add to the session?**

R: No.

**M: Okay, thank you.**

R: Thank you too.
