# Supplementary material for: Psychosocial needs among older perinatally infected adolescents living with HIV and transitioning to adult care in Kenya
Source: PLoS One. 2020 Jul 29;15(7):e0233451. doi: 10.1371/journal.pone.0233451 (PMC7390380; doi:10.1371/journal.pone.0233451)
Supplement: S1 File — (ZIP) [file pone.0233451.s002.zip › uploaded final PLOS/Reviewed Transcripts/IDI8M.doc]

**M: Okay (name) so tell me a little bit about yourself and what you enjoy doing, do you like music, football, and tell me something you like doing?**

R: I am artist and i enjoy working with computers.

**M: You do pencil drawing?**

R: Yes,

**M: What do you do on the computer, graphic design?**

R: I can do a lot of things, even hacking Facebook, Instagram accounts.

**M: I am now afraid, I don’t think can accept your friend request. You can hack my account.**

R: I knew it long time.

**M: How did you learn how to do that?**

R: I had a friend whose family had a computer and I would visit him every day and in the process I learnt how to do that and even break the IPhone codes.

**M: How does that benefit you, have you ever helped any IPhone user to break the codes?**

R: Yes, and then they paid me.

**M: Do you also break the codes for the stolen phones?**

R: If the passwords are blocked I can help them unblock.

**M: I don’t have an IPhone yet but when I do and I need help I know whom to contact.**

R: Sure.

**M: I am just wondering how long have you been coming to this clinic, how old were you went you started coming here if you can remember?**

R: I was about 5 years.

**M: You were in nursery?**

R: Yes.

**M: Do you come on your own now?**

R: Yes, I do come on my own and other times I come with my aunt.

**M: How old are you?**

R: 19 years.

**M: Are you in school?**

R: I am in form four.

**M: Are you in boarding or day school?**

R: I am in day school.

**M: You have not gone to school today?**

R: I will be reporting on Monday.

**M: Where do you go to school?**

R: Kobutho.

**M: Where is your home?**

R: I was living with a friend the last couple of days but my family lives in High-rise.

**M: Why do you stay with your friend and not family?**

R: At times my aunt scolds me a lot and to get away from her I go live with my friend for a while.

**M: She is too harsh?**

R: Yes, and I keep distance abit.

**M: Who pays your fees, is it your friend?**

R: No, my aunts pay my fees.

**M: Have you been able to get used to the clinic and the way it works, coming, registering, getting the medicine or do you struggle?**

R: Yes, I know what to do. I have been coming here since I was 5 years and now I know what to do.

**M: When did you start coming alone to the hospital?**

R: When I was around 15, 16 years.

**M: You mentioned that your auntie’s pay your fees meaning its more than one?**

R: Yes.

**M: My assumption is your parents are not alive?**

R: Yes.

**M: Both of them?**

R: Yes, both of them.

**M: Do you have a brother or sister?**

R: I have a sister.

**M: How old is she?**

R: She just cleared university and she will be starting her internship soon.

**M: You are just the two of you?**

R: Yes, we are just two.

**M: There is no big age gap?**

R: Yes.

**M: Where does your sister live?**

R: She had to relocate to Nakuru, because that is where her internship is.

**M: But while she was in campus and school whom did she live with?**

R: I lived with her, infact we were recently together in campus.

**M: You all lived with the same aunt while here in Nairobi?**

R: Yes, both here in Nairobi and then at times we go to our rural home especially during the holidays like in December.

**M: Do you choose to travel to the rural areas to avoid your aunty or just a change?**

R: I just decide to go there for a break.

**M: Whom do you stay with when you go to your rural home, your grandmother?**

R: Another aunt lives in Bomet.

**M: You came to the hospital when you were 5 years but how did you know your HIV positive status?**

R: I used to take medicine before but I never understood why, but one time during one of the clinic visits I was informed that I was HIV positive.

**M: Who informed you?**

R: One of the clinicians Lumumba and Lillian.

**M: How old were you?**

R: Around 15 years.

**M: You understood what they were talking about?**

R: Yes.

**M: How was your reaction, were you shocked, did you ask any questions?**

R: I didn’t know about my status by then.

**M: M: How was your reaction?**

R: I didn’t like it because I didn’t choose to be in this position.

**M: I understand you felt bad?**

R: Yes, really bad.

**M: How long did you have that feeling?**

R: I still feel bad, that feeling has never stopped.

**M:Has it reduced anyhow?**

R: At times I think about it and it really hurts me. That feeling cannot go.

**M: Did you get a chance to speak to your sister and aunt about it?**

R: I am closer to my sister and I tell her everything about me.

**M: Does she advise you?**

R: Yes, she does.

**M: Is she also on medication?**

R: No, I am the only one.

**M: Were you upset that you had not been informed earlier?**

R: Just knowing about it really stressed me.

**M: Did you ask the counselors any questions or they just talked to you and you listened and walked out?**

R: They explained to me and told me that my mum didn’t know about my status before she died.

**M: How old were you when he died?**

R: I was very young.

**M: You have not grown up with her?**

R: No, it’s my sister who knows more about her.

**M: How about your dad is he alive?**

R: He is not alive.

**M: Do you wish that there is something that they would told earlier that is both Lillian and Lumumba or they would have told you in a better way than they did when they did it at 15 years?**

R: I don’t think there is anything else that they would have told me, I think they did what they had to do at that point.

**M: Did you ask any questions or are there questions you wanted to ask but you never asked?**

R: There is no question that I wanted to ask.

**M: And you also mentioned that knowing your status still makes you sad to date?**

R: Yes.

**M: Is there anything that makes it difficult for you to cope with your status? Other than what you told me about your aunty?**

R: When they find me talking to a girl they really freak out, they don’t entertain me having girlfriends.

**M: That is your aunties?**

R: Even my uncles, when they find me talking to a girl they really freak out and start lecturing me.

**M: So you like girls?**

R: Yes.

**M: But there is no young man who doesn’t like girls and you are in that age group of 19 years.**

R: Yes.

**M: Tell me how has it been you trying to get that girlfriend, and have you ever met a girlfriend you became close and you wanted to tell them about your status but you decided to tell them or not? How has it been do you tell them about your status?**

R: Actually, I have never told any of them. And the few that I met and told them that we should come to the VCT majority of them don’t want to come.

**M: They don’t want to be tested?**

R: No, they don’t want.

**M: But you tried to bring them so that they can know about your status when you came to the facility?**

R: Not exactly, but I fear that if I insist she might tell other ladies and get them suspicious.

**M: Are you in day or boarding school?**

R: Day school.

**M: Do you attend the same school with your girlfriend?**

R: Yes.

**M: That is a big challenge most people have fear of disclosing their status to friends because they fear that they might go tell the others.**

R: Yes, they would.

**M: How about when you want to have sex do you use condoms. I heard some women do not like using condoms? Have you ever heard of that?**

R: I use condoms.

**M: Have you ever come across a girlfriend that didn’t want to use condoms?**

R: No, I haven’t.

**M: Are you that careful?**

R: I cannot engage in sex without protection.

**M: What pushes you to use protection?**

R: I care about that lady and I don’t want her to be in the same condition that I am. I don’t want her to hate herself the way I do hate myself.

**M: And that is the second time you have said that?**

R: Yes, I have.

**M: Why do you hate yourself yet you were not the cause you contracted it during delivery. It’s like diabetes, its hereditary and if your parents were diabetic one time you might go to the hospital and find that you are diabetic as well?**

R: I think diabetes is better.

**M: But those people have to inject themselves daily would you want to do that? But you feel diabetes is better.**

R: I feel that its slightly better.

**M: Because you can share.**

R: It is better because you cannot be discriminated because of diabetes.

**M: Have you ever faced discrimination either from family, friends, anything?**

R: I feel that way at times.

**M: You feel like you are not treated the same and you feel it is because of that story?**

R: Yes.

**M: Would you like to share any experiences of discrimination that you remember?**

R: I will let you know once I remember.

**M: Now when you need emotional support whom do you rely on?**

R: My sister.

**M: What else do you do for emotional support?**

R: I party with friends or drink.

**M: Do you feel that your stress is relieved when you drink alcohol?**

R: Yes.

**M: What else makes for you to cope with your stress, does your art release stress or not?**

R: I draw when I am more calm, not when I am stressed.

**M: When you need emotional support and your sister is not around do you have anyone that you talk to?**

R: I don’t have anyone to talk to other than my sister. I either call her or wait until she comes back then we talk about what was bothering me.

**M: Do you know anyone that is HIV positive that has managed and coped with it apart from the people who come here to the clinic, any friends?**

R: It is not easy to know anyone who is positive.

**M: How do you manage stigma, or have you told anyone to their faces to stop discriminating you?**

R: I have not told anyone.

**M: How do you cope when someone stigmatizes you?**

R: I just keep quiet, I don’t tell them anything.

**M: What do you do?**

R: I just let them finish talking then ask them what they really want from me.

**M: Are you serious?**

R: Yes.

**M: You are harsh.**

R: Not really.

**M: How has your experience been using ARV’s? Do you feel like not taking them at times?**

R: I don’t think I can start getting tired now, because I have been taking them for 19 years now and I know that my life depends on them. And if I don’t take them then I will be sick the way my mum got sick.

**M: Your mum never used to take her medication?**

R: Yes, and I have an example, like a proof of what can happen to me if I don’t take my medicines.

**M: I know you are struggling abit and I know your viral load is high, why do you think its high right now? Was there a time you didn’t take your medication or what happened for the viral load to go up?**

R: There was a time I didn’t take medicine.

**M: Why not, were you partying a lot?**

R: I used to forget.

**M: Why, what time do you take your medication for you to forget?**

R: I used to take at 6:00am and 6:00p.m and I used to forget and now it was changed to 9:30p.m once a day and that has really helped me.

**M: When did you start taking this one for once?**

R: Once.

**M: How was your viral load before?**

R: When I was on first line the viral load was high because I would forget taking medicine, at time I would be far from home around 6:00p.m and I would not be able to take medicine. Other times I had to rush home to take the medicine.

**M: Do you carry medicine that you can take if you are away from home, or that is not your thing?**

R: I used to forget all that time, when I was in boarding school but now I am in a day school.

**M: When were you in boarding school?**

R: Around form 2, 3

**M: How come you left when you were joining form 4, were you facing discrimination? Or did someone find out?**

R: It is very difficult to take your medicine while in boarding school.

**M: Why?**

R: At times you share things with your friends including your suit case and then you have to keep the medicine there and here you are trying to hide it from them, it can really stress you out. And when they see you with so many medicine they start questioning so many things about you.

**M: Did he ask you?**

R: Yes, and I told him they were for a chest infection but I am sure he would still wonder what all that medicine is for.

**M: Was it hard to take medicine when your friends were around?**

R: Yes, it was really difficult.

**M: Is it easier now to take medicine now that you are in a day school?**

R: Yes, it’s much easier, I leave home in the morning then I take my medicine at night without worrying about anyone.

**M: I want you to tell me what are your thoughts about the future and don’t tell me a professional hacker?**

R: No, I want to be behind the camera.

**M: Like a camera man or producers?**

R: Yes.

**M: You are interested in arts?**

R: Yes, I see a bright future and I talked with my sister and she is willing to support me, so I am good.

**M: You will go to multimedia or a school that does media?**

R: Yes.

**M: That is ticked and it will happened?**

R: Yes

**M: How do you see yourself 10 years from now health wise?**

R: I will be much better.

**M: Is there something you have been doing right now that is not very good and you hope that by then it will have improved? And you can be honest, I am not here to judge I am here to listen, to your challenges and other people as well, what do you hope to do different compared to what you are doing now?**

R: Most of the youths just want to have fun but I feel that with time I will be bored of doing all these kiddish things and I will be more focused.

**M: You will get tired of drinking and partying?**

R: Yes.

**M: Even sex mode?**

R: Yes, I have heard people talk about it, that you eventually get bored of these things.

**M: You are waiting to see if you will get bored as well?**

R: I am not waiting to see I know that I will get bored.

**M: Do you feel like your HIV positive status might affect or stop you from achieving your goals of working behind the camera?**

R: I don’t see how it will affect my career.

**M: Do you know any prominent, successful person who is HIV positive?**

R: I don’t know anyone else other than my mum.

**M: Do you meet any friends here, age mates that are HIV positive?**

R: It is very hard to know just by looking at them. It is not very easy to find people just opening up about their status.

**M: Okay.**

R: It is hard to trust each other because you feel like if you tell this person they might tell other people.

**M: Would you like to have a family in the future?**

R: Yes, I would.

**M: Any fears about that?**

R: No fears.

**M: You think getting a family is possible?**

R: Yes, it is possible.

**M: And status of your family as (name) when you are 29 years, 10 years down the road or you plan to get married when you are 50? Or however the time would be.**

R: I will find a suitable time.

**M: So do you have fears about the family, how it will work out. Do you feel like you will get someone to marry?**

R: Yes, I would like to get someone understanding and who will understand my status and reveal to me their status, if they do that we will get far.

**M: Do you mind getting a HIV positive or negative status partner?**

R: I don’t mind as long as we get along. And it should not be someone taking advantage of me and just wants to have fun, it should be someone willing to settle.

**M: Do you feel that these days many ladies like having fun?**

R: Yes, most of them don’t like settling down. It’s hard to find a lady who is serious about life and you can share openly about life and settling.

**M: Do you want to have children?**

R: Yes I want to have children and I want them to have a good life and not live a life similar to mine in that they have to rely on medicine daily.

**M: They should be negative?**

R: Yes, I don’t want them to hate themselves.

**M: Do you know how you can achieve that?**

R: I heard that it’s possible to have HIV negative children even if the parents are HIV positive.

**M: Yes, it’s true. Do you know how it can happen?**

R: I don’t know how that work out.

**M: Would you like to know how that happens?**

R: Yes, I would really love to know.

**M: Anything else you would like to know at your age now in terms of sexually, relationship, future, HIV? If we were planning to have health talks what topics would you like to be covered.**

R: How to get a HIV negative baby.

**M: I would like us to discuss about information or advise and where do you seek for information about anything or advise?**

R: I would prefer to get it from Google because at times when you Google you don’t get the correct information.

**M: You would like to hear from someone who has had that experience?**

R: Yes.

**M: Do you feel like if you had a role model?**

R: A role model who I also positive that would help.

**M: Have you ever attended a support group, especially if your viral load is high normally people are invited?**

R: I have never come.

**M: But you have been invited?**

R: Yes, I have.

**M: Do you feel that those sessions are beneficial or what can they do differently?**

R: I have never attended any, so I don’t know.

**M: You never attended even last year?**

R: I have never attended any of the session.

**M: At the moment would you say your viral load has increased or not?**

R: It was high when I was taking the first line medication and now that I am on the second line it has gone down and my health has improved. I used to get constant colds and I was sickly but I have not been ill for a long time so I feel the viral load has gone down.

**M: And you drink at 9:30p.m so that has helped you a lot?**

R: Yes, it has.

**M: Do you ask me to touch on what you asked about being positive and having negative children?**

R: Yes.

**M: Okay.**
