# Supplementary material for: Psychosocial needs among older perinatally infected adolescents living with HIV and transitioning to adult care in Kenya
Source: PLoS One. 2020 Jul 29;15(7):e0233451. doi: 10.1371/journal.pone.0233451 (PMC7390380; doi:10.1371/journal.pone.0233451)
Supplement: S1 File — (ZIP) [file pone.0233451.s002.zip › uploaded final PLOS/Reviewed Transcripts/IDIF09.doc]

**M: Thank you for giving us the opportunity to have this discuss and I would like to understand about your experiences in this clinic, how long have you been coming to this clinic?**

R: Since 2004.

**M: Do you always come alone?**

R: When I was young I used to come with my parents but these days I come alone.

**M: When did you start coming alone?**

R: When I turned 16 years.

**M: Would you say you are familiar with the processes at the clinic right from the reception to the pharmacist?**

R: Yes.

**M: Could you take me through the process, what happens when you get to the facility?**

R: I give out my card and they record my name and number and then I wait so that they can take my Temperature , then the weights and then I come to the counselor who talks to me to find out how I am doing and if I have any challenge and if I am okay and the doctor.

**M: Could you take me through the experience with the counselor?**

R: Usually I go to the counselor when my viral load has gone high and the counselor speaks to me and if my viral load is high then the counselor advises me on what to do to avoid it being high and at times they get upset because they had already talked to us.

**M: Could you take me through your experience with the counselor the first how was it?**

R: I was very young and I didn’t understand what was going on and whenever I would come the counselor would speak to the mum and at times she would tell her that my viral load has gone up but I didn’t understand what that meant. And then eventually I was told that I had the virus and I was also wondering what that meant and then later on in life I was told that I had a virus that is not curable but the drugs will help so that the virus doesn’t attack my body.

**M: How did you feel when you were told that?**

R: I didn’t feel bad because I didn’t really understand much.

**M: The first time your status was revealed to you, you didn’t understand much about what you were told?**

R: Yes.

**M: But gradually after understanding did that motivate you to continue taking your ARV’s?**

R: Yes, it did.

**M: What questions did you have at that time?**

R: I was just wondering how I contracted the virus.

**M: Did you ask anyone or those were mental questions?**

R: Yes.

**M: Have you ever asked anyone about it?**

R: No, I haven’t.

**M: So you have never understood how you contracted the virus?**

R: No, I don’t.

**M: Are there any other questions you didn’t feel free to ask?**

R: No, those were the main ones.

**M: Had you heard about HIV before that?**

R: Yes I did in school.

**M: Before that what information did you have about HIV?**

R: I knew that it was a disease which had no cure and it can be shared trough sharing fluids and blood.

**M: And then you were told that you now had it?**

R: Yes.

**M: How did that make you feel?**

R: I felt bad.

**M: Did you tell anyone how you felt?**

R: No, I didn’t.

**M: Even your mum?**

R: No, I just kept quiet.

**M: What happened then when you went to sleep?**

R: I just closed my eyes and slept.

**M: And in your opinion that first day you went to the counselor and your status was revealed to you, do you feel that there is something which would have been revealed to you then that would have made you feel better?**

R: No, I don’t have any questions.

**M: But you would have wished to know how you contracted it?**

R: Yes.

**M: And then since knowing your** HIV **status how have you coped with your status?**

R: I have coped well and I have continued to take my medication as prescribed.

**M: Now thinking about your future, the fact that you have to take medicine daily, how does that make you feel?**

R:It becomes a challenge when I am in school and I have to take medicine so sometimes I have to excuse myself that I am going to the toilet then I go to take medicine because I don’t want everyone to know my status. But when it comes to eating I just eat normally like everyone else, there has not been any challenge with regards to that. Even when you find them talking about HIV you just contribute and don’t let people feel like you have something you are trying to hide.

**M: You feel that if you don’t contribute to these discussions some people might start to suspect you?**

R: Yes, they might suspect you and if they see you with many drugs and you keep taking drugs all the time, then they might suspect that you are HIV positive**.**

**M: Has anyone ever seen your medicine?**

R: No, the matron keeps all the drugs.

**M: So whenever you want to take you have to go to the matron?**

R: She gives me enough medicine for a week then I go back to see her weekly or even monthly.

**M: Why are you afraid of people knowing, what is your fear?**

R: I will be ashamed and lonely because some people do not understand about HIV and they might avoid me that I might spread it to them or they might start spreading malicious things about me. And it might even turn you suicidal if everyone suddenly avoids your company.

**M: Has that happened to anyone?**

R: No, but that is my fear.

**M: Do you know anyone in school who is HIV positive?**

R: No, I don’t know.

**M: Do you suspect that you are the only one who is HIV positive in your school?**

R: I was told that we are six but I don’t know the identity of the other five.

**M: Who told you?**

R: The deputy head teacher told me that I am not alone we are six and I should feel free and I should not fear anything.

**M: So the deputy knows?**

R: Yes.

**M: How many people know about your HIV status in school?**

R: The deputy and the matron/sister.

**M: You are in a catholic school?**

R: Yes.

**M: Are there situations that make you find it hard to cope with your current situations?**

R: Taking the medicine it’s really hard, at times during the holiday when I am trying to catch up on my sleep I have to be woken up to take medicine so that really upsets me but I have to wake up.

**M: What motivates you or drives you to keep on going?**

R: The prevention bit and I don’t want to get sickly and unhealthy.

**M: If you compare your life now that you have knowledge about your status to before when you didn’t know about your status which one is better?**

R: I am now coping better because I know my status and I didn’t know what was going on, before I didn’t know what was going on and what I needed to do to keep me going but now that i am aware it’s much better. I used to wonder why I was the only one taking medicine while my younger sister was not taking any medicine.

**M: You were the only one who was taking medicine?**

R: Yes, I was but my younger sister was not taking medicine.

**M: How many are you?**

R: We are two children.

**M: Does your mother also take the ARV’s?**

R: Yes, she does.

**M: Do you take them at the same time or different times?**

R: We all take at different times.

**M: So knowing your status has helped you cope with the taking of the medicine?**

R: Yes, it has.

**M: In your opinion have you seen anyone dealing with the same issues that you are dealing with maybe they encourage you when you see how they manage the disease?**

R: I don’t know anyone.

**M: Then generally what stresses you?**

R: My education, and my future knowing that I am HIV positive I know that it will not be easy for me to get married to a HIV negative partner. So I keep wondering who will accept me and I fear that I might be lonely in the future.

**M: That stresses you?**

R: Yes, especially when the teachers keep talking about it in class and I know that I fall under that category that stresses me the fear of knowing that I might be alone in the future,

**M: Any other issues that stress you generally in life?**

R: No.

**M: Any other issues it could be anything?**

R: Maybe when you disagree with someone or even fight about something but not often. I don’t like getting into a lot of trouble.

**M: How does that make you feel when the teacher talks about the future and the possibility of you never getting married, what do you do to relive that stress?**

R: I will go and do something that I like singing, drawing.

**M: What else do you do to release the stress?**

R: Eating, if that is your passion.

**M: Now when you need emotional support whom do you rely on?**

R: My friend but back at home, we are not in the same school.

**M: So if something stresses you in school you wait until you go for your holidays?**

R: Not really but I just wait till I get home.

**M: But when you get home you speak to that specific friend?**

R: Yes.

**M: Is it a boy or a girl?**

R: A girl.

**M: What do you talk about when you talk with her, and how free are you with her?**

R: I feel very free because we are in the same situation and it becomes easy to talk about us.

**M: You talk about your status?**

R: Yes.

**M: What else do you talk about?**

R: What happened in school, incase you faced any challenge you can talk about that as well.

**M: Is there any other person you talk to about your HIV status other than your friend?**

R: No, she is the only one.

**M: How about your mum do you talk to her about your status?**

R: No, we don’t.

**M: Do you receive any support at home?**

R: Yes, when my mum reminds me to take medication, because if I fail to take medicine then I might fall ill and my health might deteriorate.

**M: So she encourages you to take your medication?**

R: Yes.

**M: Any other emotional support, does she ask you about your boyfriend and such issues?**

R: No, she doesn’t.

**M: Why not?**

R: I am currently not focusing on boyfriends issues.

**M: Would you like to have such discussions with someone about relationships?**

R: Not at the moment.

**M: What if a certain boy is interested in you what would you do?**

R: I will talk with my close friend, basically tell her that a certain boy is interested in me but he doesn’t know my status and then my friend will advise me. But for now I have to wait until I meet someone and we date for a while and I gain that confidence in them over time before disclosing my status to them.

**M: When will that be?**

R: If they start demanding for sex and I don’t want to infect them and it would be hard to explain to them why I don’t want to engage in sex with them.

**M: You are waiting until they ask for sex?**

R: Yes, and I trust them over time because right now I might tell him and he will go and tell other people that I am HIV positive and warn everyone not to befriend me.

**M: What if you are under pressure and you also want to engage in sex what would you do?**

R: I just control my feelings, by abstaining.

**M: Do you feel that a time will come when you will really need to engage in sex?**

R: Yes

**M: What will you do at that point?**

R: I will have to disclose my status and if he still wants it then we can use a condom, and if that doesn’t work then maybe we might go to a counselor for further advise.

**M: You feel confident that when you get a serious partner and consult the counselor he or she will advise you then?**

R: Yes, I am.

**M: So only one friend is aware of your status?**

R: Yes.

**M: Do you feel that there is someone who is not supporting you, either in your life or in the family, someone you expect them to support you more but they are not supporting you?**

R: No.

**M: Have you ever experienced HIV stigma?**

R: No, I haven’t but at times it’s a challenge during opening day when the teachers search our suitcases and they see you with so many drugs and they start asking you what they are for, and I am unable to explain to her. At times he or she exposes them to the other teachers and students and that makes me uncomfortable because I don’t know who is watching, because they expect you to explain because some suspect that you might be having many drugs because you want to commit suicide. So that freaks me out when everyone is just questioning what you could be suffering from that you have to take all that medicine and even the color of those drugs is so bad that everyone wonders why you have to take them, so that reduces your self-esteem when everyone is looking at your drugs.

**M: Has that happened to you?**

R: Not yet but it happened to another student but she was not HIV, she was undergoing treatment for ulcers.

**M: Why do teachers suspect that the students might be having those drugs to commit suicide?**

R: Because in high school youths undergo many challenges and some might not be able to handle them and they might opt for suicide.

**M: Any other issues that might force these youths to commit suicide?**

R: Their academics if they are not performing well.

**M: Any other reasons?**

R: Pressure when someone is forcing you to do something that you are not willing to do.

**M: Like what?**

R: Lesbianism that might pressure you, and if you refuse to do what they want they might beat you up or even take your property and hide it or even damage your property.

**M: What do they force you to do?**

R: To go and sleep in their beds, and its worse because if the school admin gets to know about it then you can either be suspended or expelled over things that you didn’t even want to be involved with in the first place.

**M: Are many girls involved in this behavior?**

R: Yes, even in other schools I have heard of cases where the girls had to be expelled from school. It could be girls who do these sexual encounters during the holidays then when they get to school maybe it’s a girls school then they need to continue but they don’t have boys around.

**M: So the lady who doesn’t want to engage in lesbianism might be pushed to commit suicide?**

R: Yes, they would, because other girls use test tubes to conduct those acts and the test tubes might damage them.

**M: Then on stigma like you had mentioned on your questionnaire at times people talk about HIV and you just have to contribute so that you don’t look suspicious how do you cope with that?**

R: Mainly what I do is talk negatively about the drugs so that I can discourage that talk from going on like I would talk about how bad the color of the drugs look like or something similar to that.

**M: So the way you cope with the stress is participating in the stories?**

R: Yes,

**M: I would like us to talk about ARV’s and how was your experience taking them and does it feel taking them?**

R: It was not a bad experience because the pills are not many, if I had to take about five pills a day then that would have been overwhelming for me. But currently I take two in the morning and one in the evening so it’s not a big challenge.

**M: At what point did you feel any challenge?**

R: When I confirmed my status and now I realized the importance of taking those drugs without fail, initially that was quite stressful.

**M: At what point did you face any difficulties taking the ARV’s?**

R: Waking up at a specific time to take the medicine and hiding it from everyone especially in school that was very difficult. At times its time for you to take your medicine and the teacher is still in class and you cannot just take medicine and take in the teacher’s presence that is really difficult. And even the teacher might accuse you of sneaking in some drugs to school.

**M: Do you feel that if the teachers knew your status it might make things easier for you?**

R: I am not sure because some teachers cannot keep a secret and they might go telling the other teachers that I am HIV positive**.** And some teachers might start degrading you because they feel like you have no use in life.

**M: Some teachers might talk so they should not be involved?**

R: Yes.

**M: What are your reasons for you to continue with your ARV’s?**

R: I want to live a long life, achieve my dreams and become healthy.

**M: Are there times you don’t feel like taking the ARV’s?**

R: Yes, when I am very sleepy and it’s time to take medicine that really annoys me.

**M: Any other times you don’t feel like taking medicine?**

R: When I am tired and at schools when the meals are not well prepared but I still have to eat because I am on medication.

**M: What do you do in that case?**

R: At times I don’t take the medicine because it tastes bad and the medicine tastes bad and if I fail to eat and take medicine then I will feel pretty weak.

**M: At times the food can taste really bad until you decide not to eat?**

R: Yes, it does and then at times I just take the medicine without eating

**M: What should be done to make it easier for you to continue taking your drugs?**

R: I would like for the manufacturer to introduce a drug that I can take for 6 months and then I don’t need to take another one for another six months.

**M: Do you know anyone else who is taking ARV’s apart from your mum and your friend?**

R: No.

**M: How does your friend manage to take her ARV’s?**

R: She is also in school and the matron has her medicine and she takes the medicine in the morning and evening just like I do but she collects her medicine daily from the matron not like I do for the entire week or month.

**M: What does she do at home, what helps her manage?**

R: Just sticking to the routine so she wakes up and takes the medicine or someone in their house can wake her up when it’s time to take the medicine.

**M: And for your mum how does she manage?**

R: She wakes me up to take medicine and she also asks us to remind her to take her medicine.

**M: She might also forget to take her medicine?**

R: Yes.

**M: Then you had mentioned about your thoughts about the future?**

R: Yes.

**M: How do you feel about joining the adult’s clinic when the time comes?**

R: That will be a challenge because there are few people in the youth clinic but the adult clinic has many adults and some are too judgmental.

**M: You feel that will be challenging?**

R: Yes.

**M: What exactly will be challenging?**

R: Having those adults around, some talk too much, and that lowers my self-esteem.

**M: Any concerns about joining the adult’s clinic, what challenges do you foresee?**

R: None its mainly fear of interacting with those adults.

**M: But everyone will just be taking their medicine so what is so bad about that?**

R: Some adults might want to talk to you and ask you things that you don’t want to talk about

**M: So you don’t want that socialization?**

R: Yes, there are some that you can socialize with but then there are others that you don’t feel like socializing with them.

**M: Where do you see yourself 10 years from now health wise?**

R: I will be healthy and I will achieve my dreams.

**M: What are these dreams you want to achieve in the next 10 years?**

R: I want to do engineering.

**M: Do you feel like your status might affect or stop you from achieving your dreams/ goals?**

R: No.

**M: Do you know any prominent, successful person who is HIV positive?**

R: There is but I forgot the name.

**M: What does that person do?**

R: It is a famous Kenyan musician.

**M: And you have seen that person achieving their dreams?**

R: Yes.

**M: Then would you like to have a family in the future?**

R: Yes, I would like to have a family, because I sit next to some girls and I hear them talking about their boyfriends and their future and you start imagining how your future would have been if you had a boyfriend.

**M: When you hear friends who have boyfriends talking about their boyfriends and how they are copying you wish to have that?**

R: Yes, I do wish to have a boyfriend.

**M: Do you have any fears in life?**

R: Yes.

**M: What are your fears?**

R: If my boyfriend in future that I really love learns about my status and leaves me for another girl.

**M: Any other fears?**

R: That boyfriend might get you pregnant and ignore responsibility or even say that they are not responsible for that pregnancy and maybe you have not finished school.

**M: Any other fears that you might face in the future?**

R: No.

**M: Do you feel like you need any information or advise on how to deal with certain issues in relationships?**

R: I would like to get information on dating a HIV negative person and not infecting them and those HIV positive mothers who give birth to children what should be done so that their children do not end up being HIV positive.

**M: Any other information?**

R: If you are HIV positive and your partner is HIV negative, then how you should your HIV status to them so that they know earlier on in your relationship and how you can help each other to avoid hatred and blaming each other in the future.

**M: When you need advice on relationships whom would you talk to?**

R: I would talk to the counselor at the facility.

**M: Any other place you can get information about relationships?**

R: There is none.

**M: Do you find the information that the counselor gives you useful?**

R: Yes, it is usually very useful.

**M: As we wind up, are there areas you feel like you need support through support groups from the facility or clinic staff, areas that you feel they should focus more on that are very important?**

R: There are seminars that invite people affected and they encourage them to live positively those were very good seminars that used to be there, thought these days they are no longer there.

**M: Did you attend those seminars?**

R: Yes.

**M: What were you thought during those seminars?**

R: How to cope being HIV positive especially in schools**, how** to cope with other people if you are HIV positive and HIV negative.

**M: You need more of that information?**

R: Yes.

**M: Any other information that will help you?**

R: No.

**M: Do you have any other comments or questions something you feel is important but we didn’t talk about it?**

R: No.
