# Supplementary material for: Psychosocial needs among older perinatally infected adolescents living with HIV and transitioning to adult care in Kenya
Source: PLoS One. 2020 Jul 29;15(7):e0233451. doi: 10.1371/journal.pone.0233451 (PMC7390380; doi:10.1371/journal.pone.0233451)
Supplement: S1 File — (ZIP) [file pone.0233451.s002.zip › uploaded final PLOS/Reviewed Transcripts/IDIF5.doc]

**M: Maybe we start by discussing. I want us to talk about your experiences at the clinic, how long have you been coming to this clinic?**

R: It has been long actually but I cannot remember how long exactly, but it’s been long since I was in class 6.

**M: That is a while back.**

R: Yes.

**M: So you come on your own always or?**

R: Yes, but there was a time I reached I think class 8 and I started coming by my own.

**M: Before that?**

R: I used to have an aunty, I was living with her and so I was taken to live with another aunt and she would bring me to the clinic.

**M: Your aunty used to bring you but when you reached class 8 you started coming by yourself?**

R: Yes. I think it was in class 8.

**M: Were you staying far from the facility?**

R: We currently live inxx,

**M: And it was easy coming all this way?**

R: Yes.

**M: Have you been able to understand the whole process in the clinic like from the reception, picking up your drugs do you know the whole process?**

R: Yes. I do.

**M: How is it?**

R: It is okay and it’s also nice. They are fair and it’s okay.

**M: And now I would like to ask your experience when the counselor talked to you about your HIV status the first time, kindly share that experience so that I can feel like I was there. When was it, how was it done?**

R: By that time I didn’t understand I was HIV positive and I used to come to the hospital with my other aunt that I used to live with before. And so we would come to the hospital and I would be given medication and then I got curious and decided to research more about it and I realized it was for HIV.

**M: You saw at the facility?**

R: Yes and also based on the questions that the counselor and doctor would ask me. And I would tell them what I heard about HIV.

**M: Did you figure out on your own?**

R: I used to look at my file and as I continued growing I would see the doctor’s notes like no status and so forth and so I think I knew on my own.

**M: And thinking of the day you met the counselor for the first time, how would you describe your experience?**

R: It was good.

**M: Why were you sent to see the counselor? Could you take me through the experience, how was it?**

R: I came when I was sick, I was very ill and then I went to speak to the counselor. I cannot recall all the details because I was very young.

**M: I understand that can be something really big in your life, being told about you HIV status, and there could be a few details that you might be remembering, what are some of these details?**

R: I honestly cannot recall.

**M: What questions did you have in mind when you were told you were HIV positive?**

R: Actually I have always wanted to ask my dad what happened and how come my mum died as a result of HIV.

**M: Yes.**

R: I had many questions but my second aunt, the one I currently liv with explained everything to me and so I let it go.

**M: Why did you want to know that?**

R: I was just curious how come my dad has never talked to me about it. And so I just let it go.

**M: And you kept asking yourself those questions?**

R: Yes, but now I stopped asking about them.

**M: I am sorry. Can we continue?**

R: Yes you can.

**M: You felt that your dad should have told you something?**

R: Yes.

**M: And your mum did you get the answers eventually what happened to your mum?**

R: Yes, I got answers from my aunt. My aunt told me that she died as a result of HIV.

**M: Was she telling you or was she talking to someone else?**

R: We just started chatting one day and so in the process she told me. And then she also told me that during that time very people had access to HIV medication and I just said it is okay.

**M: So your mum died when you were young?**

R: Yes, in 2003.

**M: And your dad is still there?**

R: Yes.

**M: The questions you had when you were your HIV status was disclosed to you was how you contracted it and also if that is what affected your mum?**

R: Yes.

**M: Any other questions you had in mind?**

R: That is all I wanted. Just to have an interaction with my dad so that he could explain everything to me. But after my aunt explained to me and I understood everything I decided with the help of my aunty to let it go and continue living my life.

**M: Okay, why were you not free to ask your dad directly?**

R: I don’t know. I love my dad and…

**M: You were unable to ask him?**

R: Not that but my aunt told me that it’s okay he didn’t tell me but if I go asking him now at this point in his life, it might trigger other emotions, because he might also have contacted it through a bad experience. And so we decided not to ask him.

**M: And you are now okay with that decision you made?**

R: Yes I am.

**M: And since that time, how have you been copying emotionally with your status?**

R: Since I have been getting support I am okay. I have accepted my status and its okay.

**M: You are okay?**

R: Yes.

**M: Now when you need emotional support whom do you rely on, other than your aunty?**

R: My aunty gives me support and another matron in school also supports me.

**M: Whom else do you rely on for emotional support?**

R: I love my parents and my dad and my sister and they also give me support by making me strong.

**M: So at least you have family support?**

R: Yes.

**M: Are there situations that make it hard for you to cope with your status?**

R: Not at the moment.

**M: But in the past?**

R: Not even in the past.

**M: There is no time you ever felt that I wish I didn’t have this status?**

R: No I believe that in this world many things happen and this is what God thought I could handle and that is my reasoning for this.

**M: You encourage yourself by saying that?**

R: Yes.

**M: What helps you cope with your HIV status?**

R: My dad because I love him so much.

**M: He helps you cope?**

R: Yes.

**M: If you compare now that you know your status and before when you didn’t know about your status, are you able to cope better now that you know your status or before you knew your status, which hone is better?**

R: Knowing my status. Because once you know your status you have to work towards making sure you are okay and you are healthy and so that you can be strong to move on.

**M: You prefer knowing your status?**

R: Yes.

**M: If you compare life now and before knowing your status, which hone is better?**

R: Now after knowing my status. You might stay without knowing and maybe something is happening to your body and you don’t know and maybe you would have stopped it from getting worse. It’s like a diabetic person, its better they know that they are diabetic and start treatment early than waiting for it to become worse.

**M: Are you aware of anyone dealing with the same things that you are currently dealing with that has influenced you or encouraged you as a result of how they are dealing with?**

R: There is a friend in school who is in form four. I shared with her my status and she told me it is okay and we have continued being friends. At times she even reminds me to take my medicine and she even takes me to the matron to take the medicine and ensures that I take the medicine. And in school you have to take the medicine infront of the matron so that you don’t throw away the medicine. There are some students who are afraid of medicine and who hide it and don’t complete the dose. But she told me that she will make sure that i take all my medication. And so I told her and she was very good to me and she is still my friend.

**M: Wao, she would take you to the matron?**

R: Yes, she would take me to the matron to ensure that I have taken my medicine without fail.

**M: Do you know anyone who is HIV that is copying well and you see them as your role models?**

R: I would say my dad because even though he has not told me he is HIV but I suspect he is HIV positive. And I also heard that he takes medicine, so he is my role model.

**M: You love your dad?**

R: So much.

**M: Does he know that you love him that much?**

R: Yes he does.

**M: But you wish that you had more contact with him?**

R: Yes.

**M: Okay let us continue. You would prefer to be talking to him more?**

R: Yes.

**M: What has created that barrier that you are unable to speak to him more?**

R: He lives far, and I live with my aunt and we meet once a while. So we don’t have much time to talk.

**M: But you miss him?**

R: Yes, I do miss him.

**M: Is your sister taking medicine?**

R: No, she is not using.

**M: Is she your big sister or younger sister?**

R: Big sister.

**M: Does she support you?**

R: Yes, she supports me a lot.

**M: do you stay with her?**

R: No, she stays in Ngara.

**M: I would like us to talk about stress and what are the things that act as a source of stress to you, generally in your life?**

R: Stress?

**M: Yes, nothing stresses you, not even one thing?**

R: Maybe thinking about my future, because I am not sure of the future because currently it is my aunty paying for my school fees. And I worry if they will continue having money and be able to pay for my tuition.

**M: Okay.**

R: But thanks to God, so far so good they have been able to pay my fees.

**M: Do they have other children?**

R: Yes they do.

**M: At least that stresses you once a while?**

R: Yes, but they always come through.

**M: And you mentioned your future?**

R: Yes.

**M: What stresses you about the future?**

R: Yes, of course I am just curious, if I will be successful in life with my condition.

**M: Yes.**

R: But I believe that I will be successful.

**M: When you think about such things they stress you?**

R: Well not really stressed but they are just concerns.

**M: When you feel stressed or discouraged what do you do to relieve stress?**

R: I listen to music, or I go chatting with my friends and my cousins, I laugh and then I forget about it.

**M: That is how you cope, music, stories…**

R: And keeping myself busy like helping my aunt with household chores, cooking, and such things.

**M: And then emotional support, whom do you rely on mostly for emotional support?**

R: My aunt since I currently live with her and she tells me to keep on fighting. At times when there are HIV programs on TV she calls me to sit and listen to their experiences.

**M: She encourages you?**

R: Yes she does.

**M: When you need emotional support, not necessarily about HIV but generally whom would you talk to?**

R: Your dad.

**M: You call him or you chat?**

R: I can call him or we chat.

**M: What about when you need any other kind of support not necessarily emotional whom do you talk to?**

R: My sister, and my aunt, matron in school and even my friend in school.

**M: Do you live in the same neighborhood with your school friend?**

R: No, she stays far. She is a Kalenjin and they stay somewhere in Nandi, those areas where the Nandis live I am not sure of the exact place. But we communicated on phone and she asked me how I was doing.

**M: And now you have cleared form four?**

R: Right.

**M: Do you feel like you will continue to communicate with her?**

R: Yes we will continue to communicate.

**M: When it comes to issues to do with HIV whom do you tend to talk to most?**

R: My aunt and also my friend, she is really nice.

**M: You will talk to your friend or aunty?**

R: Yes, but mostly my friend.

**M: To what extent would you say your family is supportive?**

R: They have very supportive, I think 100%. They are very nice.

**M: How do they support you?**

R: Encouraging me, and then when someone encourages you and shows you that you matter and you exist it feels so nice.

**M: How open are you with your friends about your status?**

R: Not all my friends know my status. It is only two friends, and one was while we were in school but I am close to one of them. But I am okay with it.

**M: What made you disclose your status to your friend?**

R: I was very sick in school and we were very close in school and I just decided to tell her maybe she might help me in school incase I need help. And so I told her and she accepted me the way I am.

**M: That is nice.**

R: Yes.

**M: And what about the other friend how did you tell her?**

R: I think she knew because the uncle was using similar medication and she knew them and so she asked me and I explained to her when we were the two of us with my other friend.

**M: How did she take it?**

R: She was accepting but I am closer to the other girl in Nandi.

**M: In your opinion if all your friends knew your status, would that affect you in any way?**

R: It doesn’t bother me because I feel that if you are truly a friend to someone you will accept them regardless of their status. So, I feel like they should just accept me the way I am and we just be happy and life goes on.

**M: You don’t feel you have a problem disclosing to your friends?**

R: I don’t think I have a problem because they are my friends in deed they will accept me the way I am and we will still be friends.

**M: Is there anyone who doesn’t support you but you wish they would support you?**

R: There is no one.

**M: Have you ever experienced HIV stigma?**

R: No. I have not personally been affected but I have seen it through other people and I feel bad also because it might not be their fault how they contracted it. It could be through an accident and some through birth it’s not their fault. So I think what some people do is so wrong because you might not understand how they contracted it and how they ended up in that situation. But personally I have not encountered it but I have seen people encountering stigma. Even in school I see some being treated badly.

**M: How are they treated?**

R: There are some teachers who cannot keep their mouths shut once they know your status and they will tell other teachers, who tell other teachers and so it spreads. And so if you don’t get support and people talk negatively it affects you and I think they should consider how other people feel because it is not their fault that they are in this situation maybe it’s not their fault that they contracted this disease.

**M: So in school it’s mainly the teachers discussing about your status?**

R: Yes they do. And even some friends in school in some instances.

**M: Has it ever happened to anyone in school?**

R: I have not experienced it directly but you hear friends talking about other students, and then there are rumors. Like in school you would hear people spreading rumors that certain students were HIV positive, it’s like it’s a really big deal while it’s not, maybe they just got themselves in that situation. And when the HIV positive gets to hear that, at times they might feel bad because everyone is talking about their HIV status and that might affect their self-esteem but generally that is what I experienced while in school.

**M: How do you feel when you hear other students talking about the other students in that manner?**

R: I avoid them and find something to do or I would tell them that I am very tired and I need to go and relax so that I can just get away from them. Because the more I stay there with them the more I feel bad.

**M: Okay.**

R: So I just move away but I do it in a way that they don’t suspect that I am not happy with what they are doing. Because some might accuse me of supporting the other student or I am not happy with them because others might be suspicious of why I am supporting the other student.

**M: Yes.**

R: And you also move away to avoid quarrels with people.

**M: What are some of the things you think you can do to cope with stress? That you feel because another person’s stigma can still affect you so how do you cope? You said you leave the crowd, what else do you do?**

R: If I was the one who had been stigmatized I would have stood up for myself and confirmed that yes I have it and its mine but at least I have confirmed my status. But I am better knowing my status, as you might leave here and go to the hospital and find that you are HIV positive. I will stand strong because as long as your family has accepted you, the rest don’t matter.

**M: You stand strong?**

R: Yes.

**M: I would now like us to talk about ARV’s and tell me your experience how it was when you started taking ARV’s? How has your experience been using ARV’s? What was it like when you started taking them?**

R:I have been taking them for long and I am just feeling okay.

**M: You didn’t face any difficulties when you started taking them?**

R: No, I didn’t.

**M: What has motivated you to continue with your ARV’s?**

R: No, it was not difficult.

**M: You know the fact that you have to take them daily how was it, and did you face any challenges?**

R: At first I used to wonder how I was going to be taking this medicine for the rest of my life and then I realized in order for me to live I have to take the medicine. So basically trying, my best to be like someone else because in this world you must make yourself happy, and be beautiful.

**M: But initially you would wonder if you were really going to be taking this medicine for the rest of your life?**

R: Yes, but I got used to taking them.

**M: Any other questions you have because I am trying to understand how people felt at first?**

R: I was young and so I used just to take.

**M: Were you taking them on your own or was someone in charge of making sure you take them?**

R: I used to be reminded to take my medication that was before I started coming to the hospital on my own. And when I started coming on my own I would remind myself.

**M: When you had a meeting with the counselor, and when you were told about your status did you also discuss about ARV’s?**

R: Yes, we did.

**M: How was that experience?**

R: It was good and I felt encouraged and so to me I would say it was good.

**M: Did you have any questions that you wanted to ask?**

R: Yes, I asked whatever questions I wanted to ask and now it’s okay.

**M: Are there times you feel like not taking your medicine?**

R: Yes, when I was in school. I don’t know what happened to me but after completing school my focus has been to get my health back to where it was.

**M: What was the issue in school?**

R: The time, at times the teacher would be in class and you are unable to go out and take medicine.

**M: So at times it would be time to take medicine and you are in class?**

R: Yes , and you know some teachers like extending the lessons. And at times the matron doesn’t come for me, because by then she didn’t know my status. And then there are some teachers who don’t allow you to leave class when they are teaching so that was also another reason for that.

**M: Okay.**

R: And then once the matron knew my status things were different because she would even come for you wherever you were. Because the matron would come to class for me and the teacher cannot refuel me to go out when the matron asks for me.

**M: So the matron really helped you a lot?**

R: Yes.

**M: Before the matron started to help you what would you do when the classes delayed, would you take the medicine late or you would take the following day?**

R: I would just wait and take medicine the following day. But now I understand that even if I am unable to take at the specific time, I should not skip taking my medication its better late than never and that is information I didn’t have then.

**M: What challenges do student taking ARV’s face?**

R: At times stigma, stress from parents if they don’t support them.

**M: Any other thing?**

R: Mainly that.

**M: What should have been done to make it easier for you to continue taking your drugs during that time you were struggling?**

R: I am really not sure but maybe I should have told the matron earlier, because the matron can come for you when you fail to take your medication.

**M: Were you the one who disclosed your status to the matron?**

R: No, but I used to have constant pneumonia attacks and I would spend too much time in the hospital, so my dad came and explained to the deputy principal and matron about my status and so I had to keep the medicine with the matron.

**M: You used to get pneumonia attacks while in school?**

R: Yes.

**M: Then after the matron knew and started following up with you regarding your medication did the pneumonia attacks reduce?**

R: Yes they reduced actually. But actually she came to know about it in form four , because form four is crucial time normally in school and your health has to be really good. And so she knew about my status while I was in form four.

**M: What has motivated you to continue with your ARV’s?**

R: I think it’s for me to be strong, accomplish my missions, visions in life. Because everyone has a purpose in life and there are goals they want to achieve and I want to achieve mine. Maybe I was brought into this world to help other people and so that is the reason I continue taking my medicine. And also for the love I have for my sister and my dad.

**M: And you mentioned that your dad takes ARV’s?**

R: Yes.

**M: Is there anyone else who takes ARV’s?**

R: I just heard that there is a certain aunt of mine who lives in Migori that is also taking ARV’s,

**M: But it’s just stories?**

R: Yes.

**M: How do they manage to take medicine as required in your own view?**

R: I have never seen them actually but I just heard about it. But I would say they are managing well because they are healthy.

**M: And now that you have cleared form four, how do you feel about joining the adult’s clinic?**

R: I am okay since I am old enough.

**M: Do you feel ready?**

R: Yes, I am ready.

**M: Any concerns about joining the adult’s clinic, moving from the adolescent’s clinic, what challenges do you foresee?**

R: I am also an adult so I don’t mind it’s okay.

**M: You don’t mind moving from the adolescent clinic or have you already left that clinic?**

R: I think I did because these days I come on Fridays and not Wednesdays the way I used to come.

**M: You are okay?**

R: Yes, I am.

**M: Where do you see yourself 10 years from now health wise?**

R: I will be doing very great, maybe supporting other people so that they don’t go through what I did. And when you get support it really makes you happy and everyone deserves that support.

**M: Thinking of your personal experience, is there any experience that you would not like anyone to go through?**

R: That moment when I was sick and I had to go to the hospital, the medication, the injections, loss of appetite, I felt weak and I couldn’t walk and the financial aspect because I needed medicine that had to be paid for and so I wouldn’t want anyone to go through a similar experience.

**M: Are there goals you want to achieve in the next 10 years?**

R: I want to be successful in future.

**M: You want to be successful in what areas?**

R: I want to have a good job and then be surrounded by people who encourage me and to be back with my sister and my father.

**M: Do you think about family, and marriage?**

R: I have not been thinking about that but obviously that would come up in future.

**M: Would you like to have a family in the future?**

R: Yes, I would like to have a family in future but before that I would like to be successful so that my children can have a good future. And when you have a good future at least you can manage yourself rather than being a dependent on other people.

**M: Do you feel that your HIV status might affect your future goals?**

R: I don’t think so because I choose to live my life and at this moment even if someone knows that I am HIV positive I will not mind. It’s like a normal thing, just like a diabetic or TB patient and I don’t think it can hinder me from achieving my goals.

**M: Do you know any prominent, successful person who is HIV positive and has achieved their goals?**

R:At times when we interact with the nurses they tell us there are a lot of successful people with HIV, though they don’t mention them but they tell us that and that also encourages me.

**M: Do you have any fears about the future in relation to your future family?**

R: No, I don’t have.

**M: And do you ever need in information or advise on how to deal with relationships?**

R: I am not currently in any relationship.

**M: It could be any relationship, with your parents, with your friends?**

R: We need encouragement, if you have a boyfriend or girlfriend and you are HIV positive. Because there might be chances whereby once people know your HIV status they might not be willing to have any relationship with you. But maybe you love them, so I think they should be educated because at times it’s not our fault that we are infected. It could be as a result of many other causes like through parents.

**M: But is there any information that you require? Because at times you might be having questions but you don’t know where you can get that information?**

R: Information about my career.

**M: But with relationships do you require any information?**

R: No. I don’t.

**M: Are you perfect?**

R: Not really but I don’t have any relationship questions at the moment.

**M: Incase you need information or advise about relationships where will you go?**

R: My friend and my sister.

**M: As we wind up are there areas you feel like you need support through support groups from the facility or clinic staff, that you feel they should focus more on a certain kind of information that will really benefit you?**

R: I don’t think there is anything so far. I think they touch on everything that I need, the most important issues. And the first time I came I realized that they touched on everything that I needed to know because we have parents who don’t have that courage to speak to their children about certain issues. And so I feel that they covered anything.

**M: Is there any specific topic that you have more interest in and you would like them to cover more, so that you get more information?**

R: Actually right now I don’t think there is anything.

**M: It’s okay and so if we were to have a couple of open session so that people discuss and share information that is useful, would you be interested to attend?**

R: Yes they are very nice, because when you see many people opening up it is very good. I think if I had been attending more support group sessions I would not have relaxed on taking my medication. If I had been coming maybe once or twice I would have been more encouraged and as a result I would have remembered to take medicine. So those groups are very nice.

**M: And you would be interested to attend such sessions?**

R: Yes.

**M: Incase we have such discussions during the holidays how often do you want them to be, every week, every month , every year?**

R: Every week because they give you support and its very nice. It also helps you to have morale.

**M: Anything else?**

R: No.

**M: Do you foresee any challenges you might get that would stop you from attending such sessions?**

R: Lack of fair, like I come from Ongata Rongai and its far and maybe there is no fare. Or maybe I might be invited to attend and I am somewhere else, maybe I travelled and I cannot attend on that day and time. Or maybe I am in school or in boarding school.

**M: Any other comment?**

R: It is nice, encouraging and I have learnt a lot.

**M: Thank you so much.**

R: Welcome.
